# Stereoselective Synthesis of the I–L Fragment of the Pacific Ciguatoxins

**DOI:** 10.3390/toxins12120740

**Published:** 2020-11-24

**Authors:** J. Stephen Clark, Michael Popadynec

**Affiliations:** School of Chemistry, Joseph Black Building, University of Glasgow, Glasgow G12 8QQ, UK; m.popadynec@live.co.uk

**Keywords:** pacific ciguatoxins, natural product, polycyclic ether, ring-closing metathesis, Tsuji-Trost allylation

## Abstract

The I–L ring system found in all the Pacific ciguatoxins has been prepared from a tricyclic precursor in a highly stereoselective manner. Subtle differences in the reactivity of the enones present in the seven- and eight-membered rings of the tricyclic ether starting material have been exploited to allow selective protection of the enone in the eight-membered ring. Subsequent distereoselective allylation of the seven-membered ring has been accomplished by a palladium-mediated Tsuji-Trost reaction. The K-ring methyl and hydroxyl groups have been installed in a highly stereoselective manner by sequential conjugate reduction and enolate oxidation reactions. Ring L has been constructed by a use of a novel relay ring-closing metathesis reaction to complete the tetracyclic framework, which possesses the functionality necessary for elaboration of rings I and L and the introduction of ring M.

## 1. Introduction

### 1.1. Structures and Bioactivities of the Ciguatoxins

**Figure 1:**
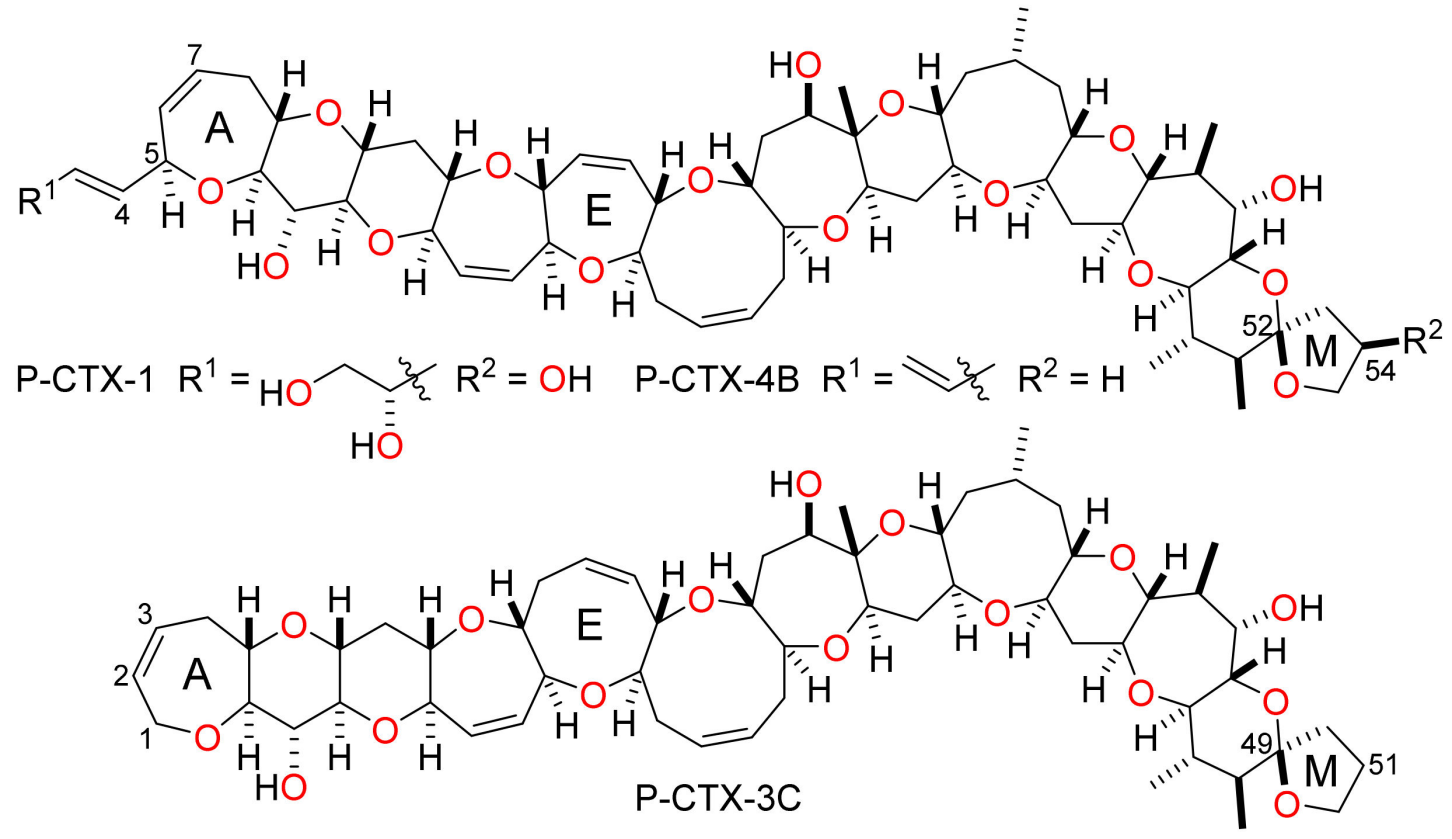
Representative Pacific ciguatoxins.

The ciguatoxins are a family of large and structurally complex fused polycyclic ether natural products of marine origin. Although the ciguatoxins had been implicated as the causative agents of ciguatera poisoning in humans since the 1960s, it was not until the late 1980s that the first structures of members of the ciguatoxin family—P-CTX-1 and P-CTX-4B—were fully elucidated by Yasumoto et al. (Figure 1) [1,2]. Subsequently, more than 30 other ciguatoxins have been isolated from marine dinoflagellates or from fish and marine organisms that accumulate the toxins by predation. The ciguatoxins can be grouped as Pacific, Caribbean or Indian according the ocean or sea where samples containing each toxin were first collected [3,4,5,6]. Pacific ciguatoxins are the most numerous and well-characterised of the ciguatoxins and this group accounts for more than three quarters of the ciguatoxins that are known [4,5,6]. Four Caribbean ciguatoxins have been characterized—C-CTX-1 and C-CTX-2 are equilibrating lactols and C-CTX-3 and C-CTX-4 are diastereomers that arise by reduction of C-CTX-1/C-CTX-2 [7,8]. A further eight Caribbean ciguatoxins have been detected by mass spectrometry but have yet to be characterised and the structures of the six ciguatoxins—I-CTX-1–6—isolated from samples collected in the Indian Ocean have yet to be elucidated [6,9,10].

The Pacific ciguatoxins can be divided into two distinct structural sub-groups. The first is exemplified by P-CTX-1 and P-CTX-4B, and the second by P-CTX-3C (Figure 1) [3,5]. Natural products in the first group have a four-carbon chain attached to the A-ring (C-5) and a seven-membered E-ring. This group comprises more than a dozen congeners that have a variety of structural modifications such as a hydroxyl substituent at C-54, inverted configuration at C-52, a seco M-ring or oxidation at the C-4 and/or C-7 positions. The second and larger group is based on a P-CTX-3C skeleton. Compounds in this group have an expanded (eight-membered) E-ring and lack the A-ring side chain found in P-CTX-1 and related ciguatoxins. Variations on the P-CTX-3C structure include A-ring and M-ring seco compounds, a C-49-epi compound (P-CTX-3B) and oxidised analogues in which there is a hydroxyl group at the C-51 position, and/or the A-ring alkene is replaced by a C-2 carbonyl group, a C-2 hydroxyl group or a 1,2-diol.

The ciguatoxins are extremely potent neurotoxins and some of them have been shown to have LD_50_ values of 4 μg/kg or lower in mice. Ingestion of fish or seafood that is contaminated by ciguatoxins causes a type of food poisoning known as ciguatera that can result in the victim experiencing a myriad of unpleasant and sometimes severe neurological (both peripheral and central nervous system), cardiovascular and gastrointestinal symptoms [11]. It has been estimated that tens or even hundreds of thousands of people suffer from ciguatera poisoning each year. Symptoms can persist for many months or even years and, in extreme cases, ciguatera food poisoning can be fatal. The pharmacological effects of the ciguatoxins result primarily from their ability to cause persistent activation of voltage-gated sodium channels in nerve membranes and thereby prevent the nerve repolarisation that is essential for normal nerve function [12,13]. There is also evidence that the ciguatoxins can block voltage-gated potassium channels [14].

### 1.2. Total Syntheses of Pacific Ciguatoxins

The ciguatoxins are daunting targets for total synthesis because of their size, their stereochemical complexity, the large number of rings they possess and the high proportion of synthetically challenging medium-sized cyclic ethers in their trans-fused polycyclic structures. The synthetic challenges presented by the ciguatoxins combined with their potent neurotoxic activities makes them highly alluring synthetic targets and many research groups have performed methodological studies in which substantial fragments or smaller sub-units of various ciguatoxins have been constructed. However, despite a continuous stream of highly innovative synthetic work spanning more than two decades, the ciguatoxins have proved to be very difficult to synthesise. The only successes in this area have been achieved by Hirama et al., who reported the first total synthesis of a ciguatoxin—P-CTX-3C—in 2001 followed by the total syntheses of 51-hydroxy-P-CTX-3C and P-CTX-1 in 2006 [15,16,17], and by Isobe et al., who completed a total synthesis of P-CTX-1 in 2009 [18]. These elegant and impressive syntheses are the only complete total syntheses of members of the ciguatoxin family of natural products to have been reported.

### 1.3. Proposed Synthetic Strategy and Previous Synthetic Work Concerning the Total Synthesis of P-CTX-3C and Related Ciguatoxins

We have been engaged in the development of new reactions, tactics and strategies for the rapid and efficient synthesis of fused polycyclic ether arrays for many years [19]. As part of this research program, we have invented and explored a variety of iterative and bidirectional strategies to assemble large fragments of fused polycyclic ether natural products of marine origin such as the brevetoxins, gambieric acids and ciguatoxins [20,21,22,23]. In our previous studies concerning the synthesis of P-CTX-3C, the A–E fragment of the natural product was assembled by a route in which ring-closing metathesis (RCM) reactions were used to construct the rings in an iterative manner [18]. We have also reported a novel bidirectional RCM approach to the synthesis of the tricyclic I–K fragment of P-CTX3-C from a simple monocyclic precursor and now present the results of studies concerning its elaboration to give an I–L fragment that can serve as an advanced intermediate for the preparation of the terminal fragment of any of the Pacific ciguatoxins [24].

**Figure 2:**
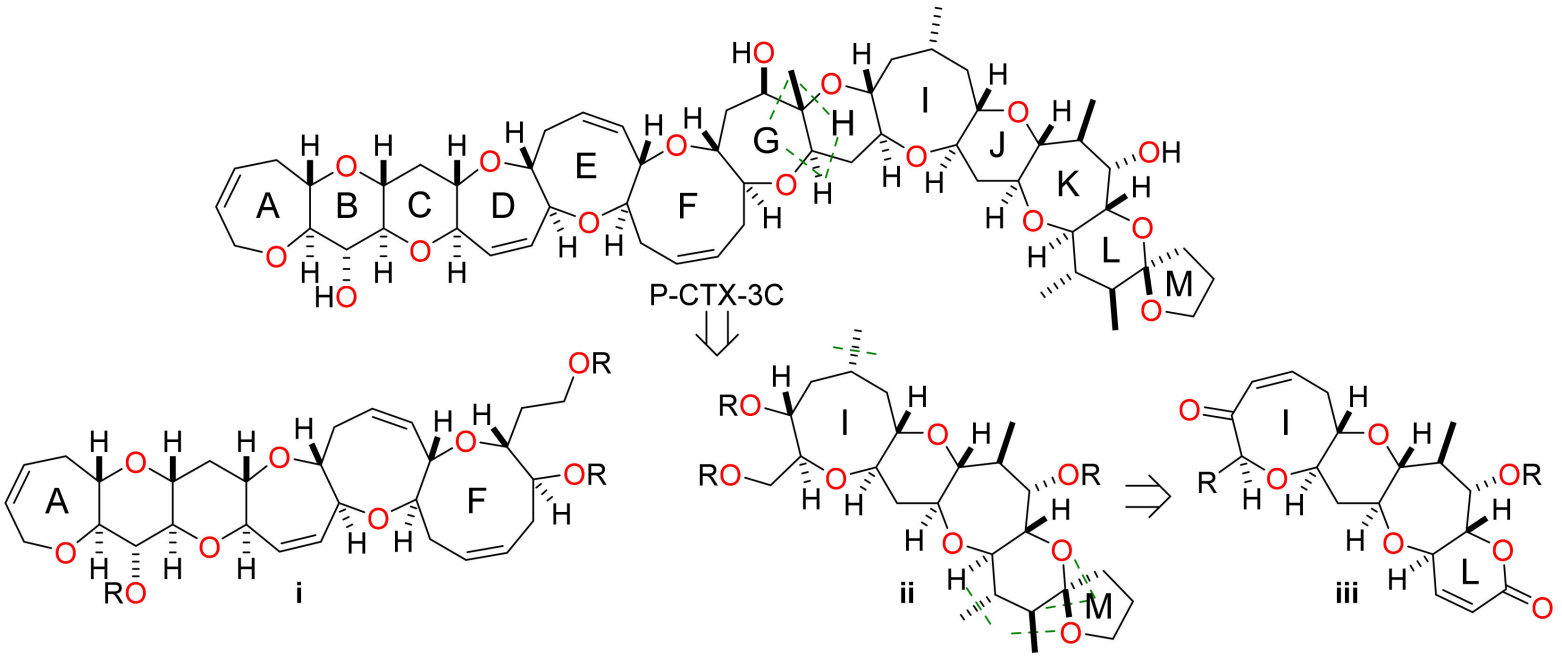
Retrosynthetic analysis of P-CTX-3C (**1**) to reveal the key A–F and I–L fragments.

Retrosynthesis of P-CTX-3C commences with disconnection through rings G and H to give two fragments—the hexacyclic A–F fragment **i** and the pentacyclic I–M fragment **ii**—that are of similar size and structural complexity (Figure 2). The focus of the synthetic work described herein was the I–M fragment **ii**. Disconnection of this pentacyclic system by removal of the methyl substituents in rings I and scission of the M-ring spiroacetal leads to the tetracyclic fragment **iii**. This intermediate contains an α,β-unsaturated ketone in ring I and an α,β-unsaturated lactone in ring L to enable introduction of the methyl substituents by conjugate addition in the case of ring I and sequential conjugate addition and enolate alkylation in the case of ring L.

**Scheme 1:**
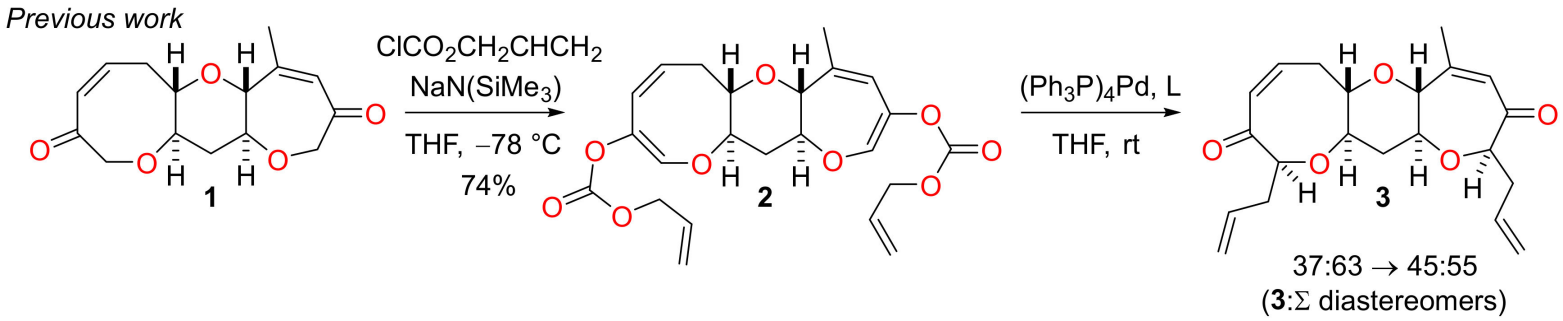
Functionalisation of the I–K fragment by bidirectional double Tsuji-Trost allylation.

In previous work concerning the synthesis of the I–M fragment of P-CTX-3C and related ciguatoxins, we have demonstrated that the tricyclic sub-unit **1** can be prepared from a simple tetrahydropyranyl precursor by double ring-closing metathesis (RCM) and that subsequent bidirectional functionalisation of the diketone **1** is possible by sequential double allyl enol carbonate formation and double palladium-mediated Tsuji-Trost allylation (Scheme 1) [24,25]. Although it was possible to direct the double Tsuji-Trost reaction of the substrate **2** to give just two of the four possible diastereomeric products by the choice of a suitable chiral ligand, we were unable to identify a catalyst that would deliver the required diketone **3** as the sole or even predominant isomer. Thus, it was necessary to revise the synthetic strategy in order to allow rings I and K to be differentiated and functionalised independently thereafter. The following discussion provides an account of construction of the I–L sub-unit by such an approach.

## 2. Results and Discussion

**Figure 3:**
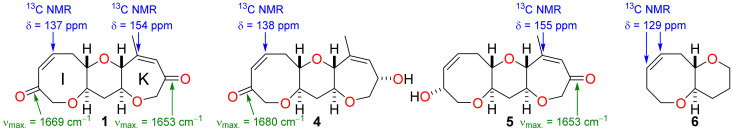
IR and ^13^C data for the bis-enone **1** and comparison to data for the related compounds **4**–**6**.

**Scheme 2:**
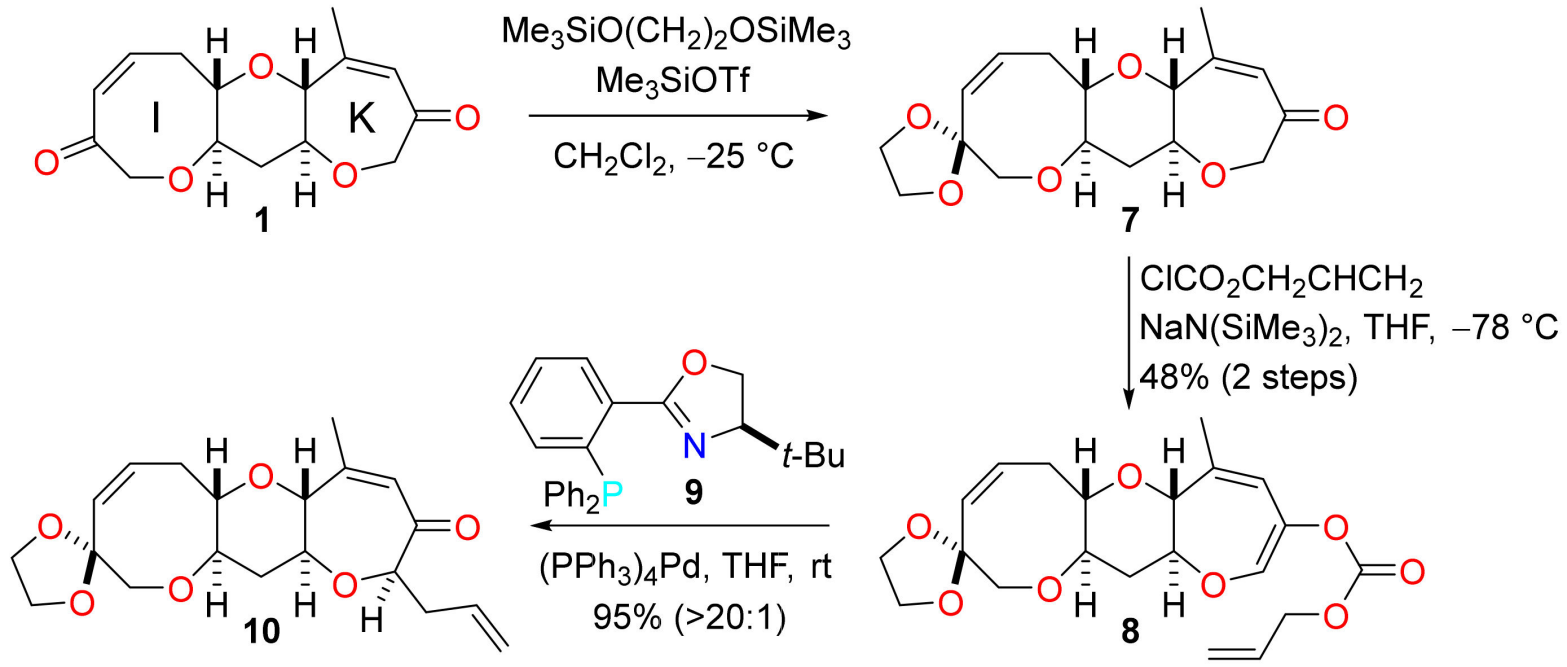
Selective protection of the I-ring carbonyl group and subsequent Tsuji-Trost allylation of ring K.

It was not possible to perform simultaneous and diastereoselective bidirectional functionalisation of the bis-enone **1** by double Tsuji-Trost allylation [24,25] and so it was necessary to elaborate rings I and K independently in order to extend the fused polyether framework (Scheme 2). The tricyclic bis-enone **1** is quasi-symmetric and so independent functionalisation of rings I and K by discrimination between reactive functional groups appears to be a challenging task at first glance. However, spectroscopic analysis of the bis-enone **1** reveals subtle structural differences between the enone functionality in rings I and K. In the IR spectrum of **1**, the carbonyl group in ring I has a stretching frequency of 1669 cm^−1^ whereas the carbonyl group in ring K has a stretching frequency of 1653 cm^−1^—the identities of the carbonyl peaks in the IR spectrum of **1** were established unambiguously by comparison to data for the hydroxy ketones **4** and **5**, produced by partial reduction of the diketone **1** (Figure 3). The IR data suggest that the degree of enone conjugation in the eight-membered I-ring is significantly lower than that in the seven-membered K-ring. This finding was further supported by ^13^C NMR spectroscopy where the β-carbon of the enone in ring I has a chemical shift (137 ppm) that is more typical of an unconjugated alkene at this position in an eight-membered cyclic ether, as can be seen by comparing the chemical shifts for the alkene carbons in the simple fused bicyclic ether **6** [26], whereas the corresponding carbon in ring K has a chemical shift (154 ppm) for the enone β-carbon that is consistent with a fully conjugated enone. The significant differences in the degree of conjugation in the enone groups suggested that it might be possible to functionalise them selectively by exploiting differences in their chemical reactivity. Indeed, there are pertinent examples of highly selective acid-mediated acetal formation in polycyclic substrates that contain both an enone and unconjugated ketone and so this approach was used to protect the I-ring carbonyl group selectively [27].

Completely selective protection of the I-ring carbonyl group as a dioxolane was accomplished by reaction of the bis-enone **1** with bis(trimethylsilyl)ethylene glycol in the presence of trimethylsilyl trifluoromethanesulfonate (Scheme 2) [28]. The enone **7** was then converted into the allyl enol carbonate **8** by deprotonation with sodium bis(trimethylsilyl)amide and enolate trapping with allyl chloroformate. Tsuji-Trost allylation was then performed thereafter by treatment of the allyl enol carbonate **8** with the complex generated from tetrakis(triphenylphosphine) palladium(0) and the (*R*)-*t*-butyl-PHOX ligand (**9**) in situ [24,25,29]. The reaction was highly stereoselective (>95:5 *dr*; minor isomer not detectable by ^1^H NMR analysis) and delivered the enone **10** in 95% yield (Scheme 2). The stereochemical outcome of this and subsequent diastereoselective reactions was confirmed by ^1^H NMR NOE analysis (for details, see Supplementary Materials).

**Scheme 3:**
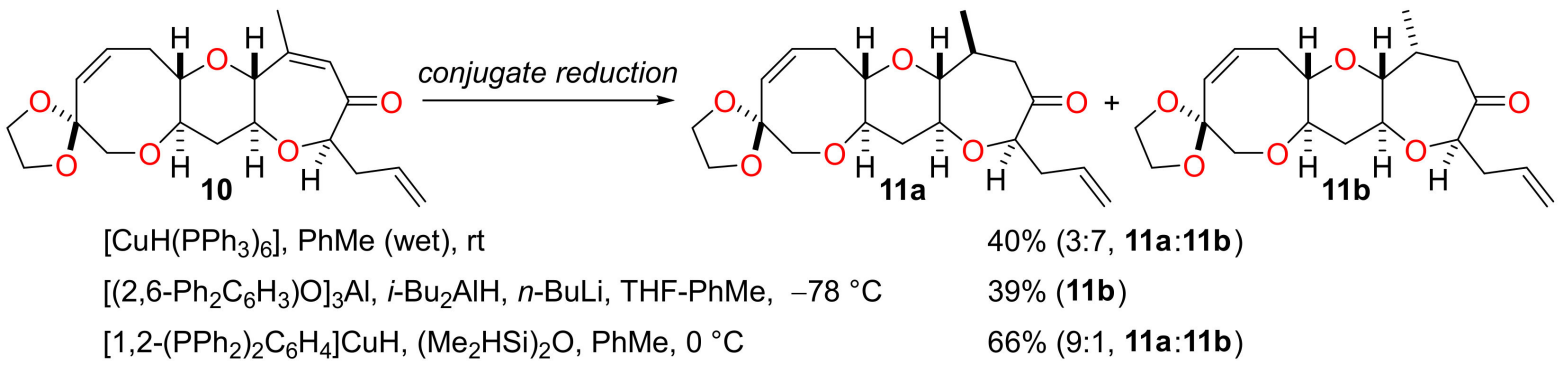
Conjugate reduction of the enone **10**.

Ring K was elaborated further by conjugate reduction of the enone (Scheme 3). Reduction of the enone **10** with Stryker’s reagent [30] resulted in a mixture of the diastereomeric ketones **11a** and **11b** (3:7 ratio) in which the required diastereomer **11a** was the minor component. This reaction also suffered from the problem that it did not proceed to completion, even when an excess of the reducing agent was used. A conjugate reduction protocol developed by Yamamoto et al. was deployed with the expectation that it would solve these reactivity and selectivity problems [31]. Coordination of the ketone to aluminium tris(2,6-diphenylphenoxide) followed by treatment with the reductant generated by the reaction of diisobutylaluminium hydride with *n*-butyllithium afforded the ketone **11b** in 39% yield and the diketone (13% yield) resulting from cleavage of the acetal protecting group of **11b**; none of the required diastereomer **11a** was obtained from the reaction. Stereocontrolled conjugate reduction of the enone **10** to give the required ketone was finally performed in good yield by reaction with the ‘hot’ Stryker’s reagent generated from 1,2-bis(diphenylphosphino)benzene, copper(II) acetate hydrate and 1,1,3,3-tetramethyldisiloxane as described by Lipshutz and co-workers [32]. Reduction of the enone **10** with this reagent at 0 °C in toluene afforded a diastereomeric mixture of the ketones **11a** and **11b** (combined yield of 66%; 9:1 ratio) in which the required isomer was the major product. The diastereomeric ketones **11a** and **11b** could not be separated by standard silica gel chromatography.

**Scheme 4:**
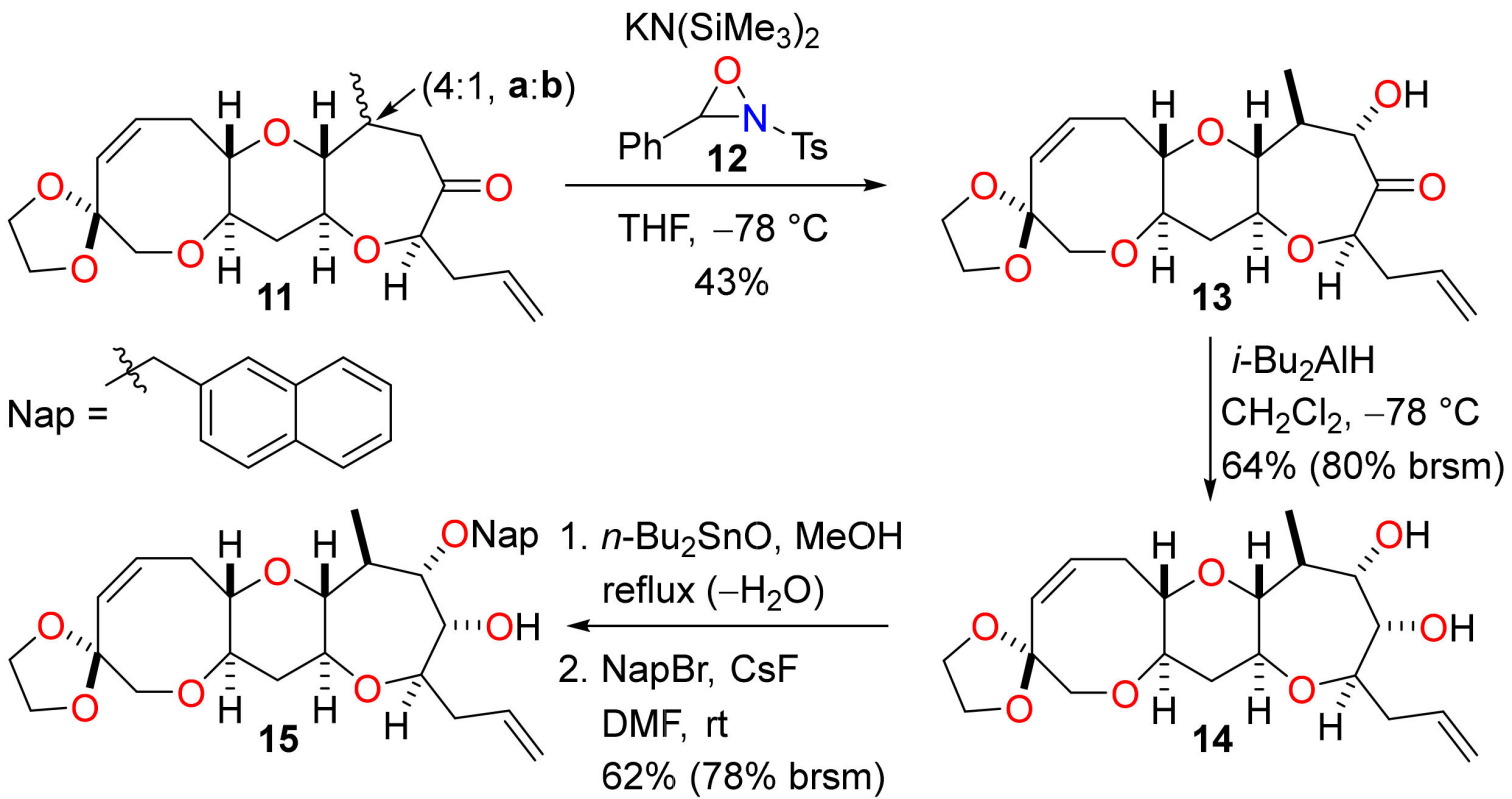
Hydroxylation and further elaboration of ring K.

Stereoselective conjugate reduction of the enone **10** enabled the requisite K-ring hydroxyl group to be installed by enolate oxidation (Scheme 4). Regioselective deprotonation of a mixture of ketones **11a** and **11b** (4:1 ratio) with potassium bis(trimethylsilyl)amide and reaction of the resulting enolate with the *N*-sulfonyl oxaziridine **12** [33,34] produced the α-hydroxy ketone **13** in 43% yield (54% when adjusted to account for the isomer ratio of starting material **11**) and with a very high level of diastereocontrol. The same transformation was performed by generation of the triethylsilyl enol ether and subsequent Rubottom oxidation with 3-chloroperoxybenzoic acid [35], but poor yields were obtained and so this sequence was not a viable alternative to direct enolate oxidation. The α-hydroxy ketone **13** was then subjected to stereoselective reduction with diisobutylaluminium hydride to give the diol **14** [36]. The stereochemical outcome of the reaction can be explained by a reaction mechanism in which complexation of the aluminium reagent to the hydroxyl and carbonyl groups produces a cyclic chelate and addition of diisobutylaluminium hydride occurs from the top face of the molecule (as drawn). At this stage, it was necessary to differentiate the hydroxyl groups of the *syn* diol **14** to enable construction of ring L. Selective protection of the hydroxyl group adjacent to the K-ring methyl substituent was accomplished by reaction of the diol **14** with dibutyltin oxide to give a cyclic dialkylstannylene acetal followed by regioselective fluoride-mediated alkylation with 2-bromomethylnaphthalene [37,38]. The Nap-protected alcohol **15** was obtained in reasonable yield and a small amount of unreacted diol **14** was recovered from the reaction.

**Scheme 5:**
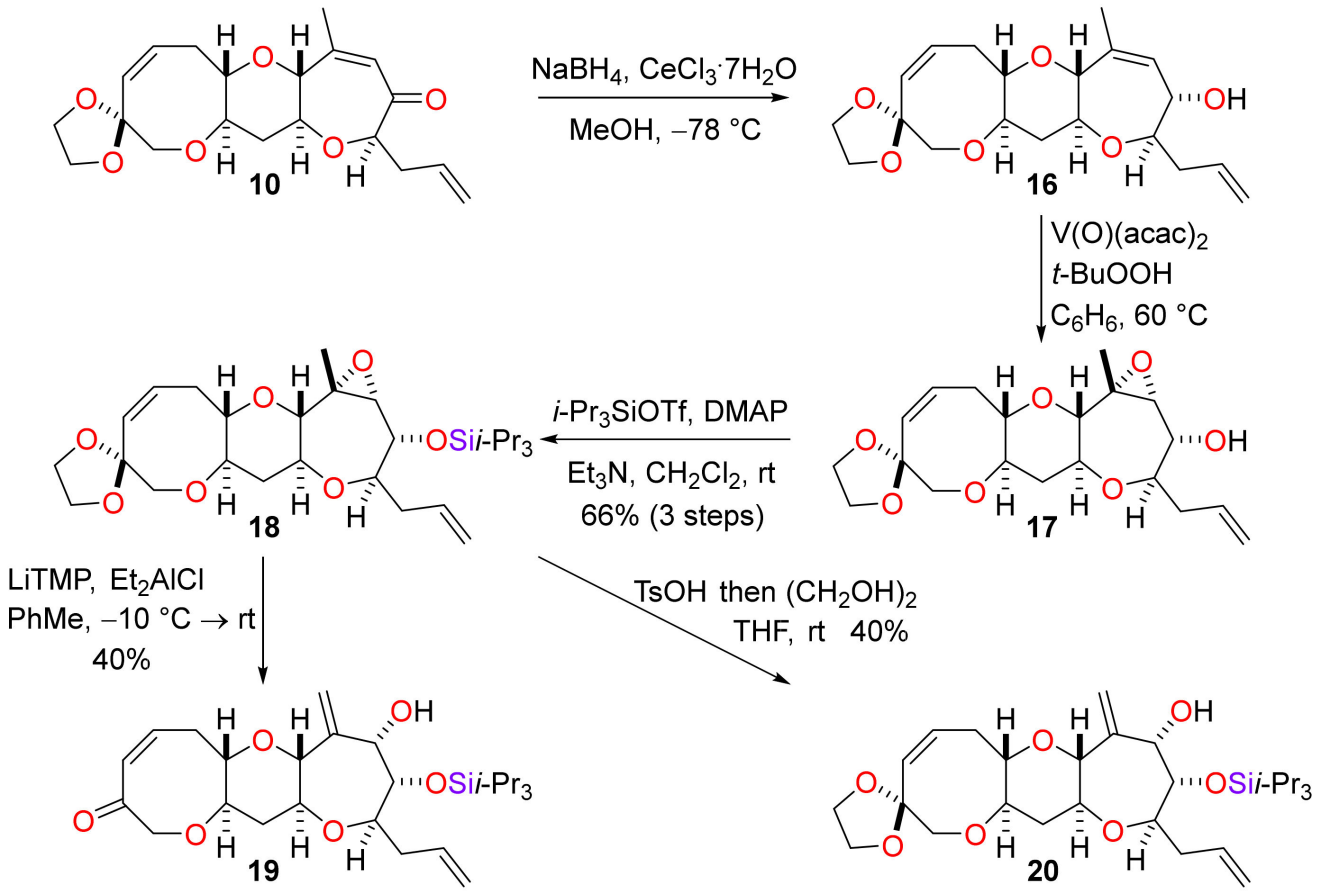
An alternative approach to the elaboration of ring K.

An alternative approach to functionalisation of ring K that did not involve sequential conjugate reduction and enolate oxidation was explored (Scheme 5). The tricyclic enone **10** was first subjected to Luche reduction which proceeded to give the allylic alcohol **16** in excellent yield and with a high level of diastereocontrol. Directed epoxidation of the allylic alcohol with *t*-butyl hydroperoxide mediated by vanadyl acetylacetonate produced the epoxide **17** in a highly stereoselective manner. Protection of the hydroxyl group as a triisopropylsilyl ether afforded the epoxide **18** in a yield of 66% from the enone **10**. It was now possible to rearrange the epoxide to give an allylic alcohol by reaction of the epoxide **18** with lithium 2,2,6,6-tetramethylpiperidide in the presence of the Lewis acid diethylaluminium chloride [39]. The allylic alcohol **19**, in which the acetal on ring I had been cleaved under the reaction conditions to reveal the enone, was obtained. Rearrangement of the epoxide **18** by treatment with *p*-toluenesulfonic acid was also successful; in this case ethylene glycol was added to the reaction mixture to ensure that the acetal protecting group was retained in product **20**. In both cases, the yield of the rearranged product was modest. Unfortunately, subsequent chemoselective and stereoselective hydrogenation of the exocyclic alkene to produce the K-ring methyl substituent proved to be difficult to accomplish. It was expected that selective directed hydrogenation of the allylic alcohol **20** would be possible by use of Crabtree’s catalyst [40]. However, the hydrogenation reaction did not take place when a catalyst loading of 1 mol % was used and decomposition of the substrate occurred when higher catalysts loadings (≥10 mol %) were employed. When Wilkinson’s catalyst was used to hydrogenate the alkoxide generated by deprotonation of the allylic alcohol **20** with sodium hydride in the presence of 15-crown-5 [41,42], the terminal alkene in the side chain was reduced instead of the exocyclic 1,1-disubstituted alkene in ring K.

**Scheme 6:**
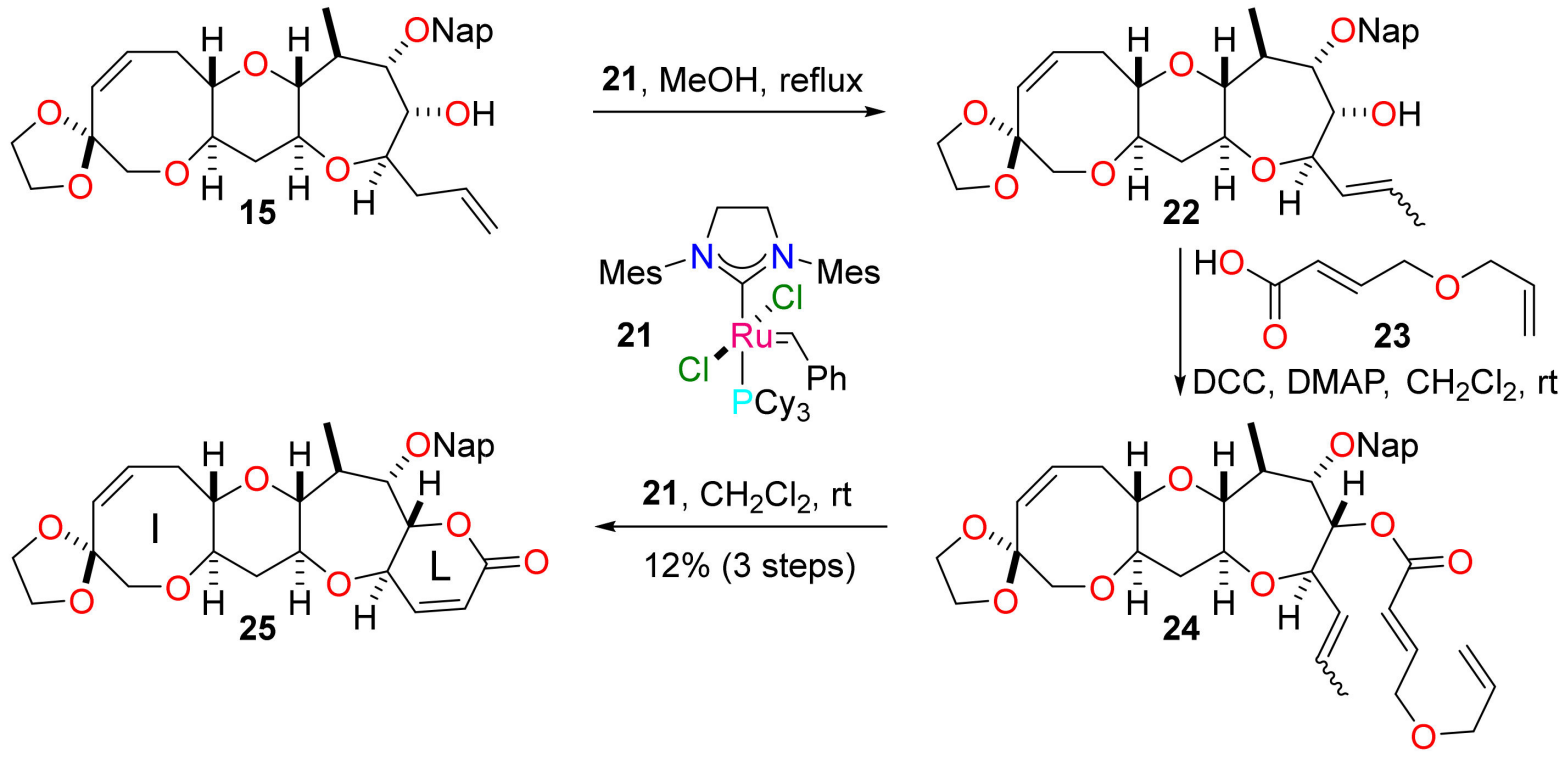
Use of relay RCM to construct ring L and form the I–L ring system of the Pacific ciguatoxins.

Construction of ring L to complete the tetracyclic ciguatoxin I–L core framework was accomplished as shown in Scheme 6. The allyl side chain in the alcohol **15** was subjected to isomerisation by treatment with the ruthenium complex **21** in methanol at reflux to give the alcohol **22** as a mixture of alkene isomers (*E*/*Z*) along with some side-products [43]. At this juncture, construction of ring L as a pentenolide by conversion of the alcohol **22** into a simple acrylate ester followed by direct RCM was considered. However, there were concerns regarding the rate of initiation of the metathesis reaction because the substrate would contain an acrylate and an internal alkene branched at the α position; consequently, a relay RCM reaction was employed as a precaution [44]. Subjection of the alcohol **22** to Steglich esterification with the known carboxylic acid **23** [45] delivered the relay metathesis precursor **24** and subsequent treatment with the ruthenium complex **21** resulted in relay RCM to give the lactone **25** corresponding to the tetracyclic framework found in the I–L ring system of the Pacific ciguatoxins. The modest yield for the three-step sequence implies an average of 50% per step; it can be accounted for by the scale on which the reactions were performed and by-product formation during isomerisation (**15** → **22**). The unsaturation in ring L would enable subsequent introduction of the requisite methyl substituents by conjugate addition with a suitable methyl nucleophile and trapping of the resulting enolate with a methyl electrophile [46].

## 3. Conclusions

The synthesis of the I–L ring system of the Pacific ciguatoxins has been accomplished in 10 steps starting from the tricyclic bis-enone **1**. Elaboration of ring K by stereoselective Tsuji-Trost allylation was accomplished after selective protection of the I-ring enone by acetal formation. The configuration at the methyl-bearing stereogenic centre in ring K was controlled during conjugate reduction of the enone and the hydroxyl substituent was installed in a highly stereoselective manner by enolate oxidation according to the Davis procedure. Stereoselective ketone reduction followed by selective protection of the resulting 1,2-diol then allowed the metathesis trigger to be attached to the K-ring by esterification of the hydroxyl group. Relay RCM, mediated by the Grubbs second generation ruthenium complex, resulted in formation of ring L and completed the tetracyclic framework with the functionality necessary for subsequent elaboration of ring I, installation of the methyl substituents in ring L and construction of ring M.

## 4. Materials and Methods

Air- and moisture-sensitive reactions were performed under an atmosphere of argon in flame-dried apparatus. Tetrahydrofuran (THF), toluene, dichloromethane and diethyl ether were purified using a Pure-SolvTM 500 Solvent Purification System. Other dry solvents and starting materials were obtained from commercial sources and used as received unless stated otherwise. Petroleum ether (pet. ether) used for column chromatography was the 40–60 °C fraction. Triethylamine and 2,2,6,6-tetramethylpiperidine were distilled and stored under argon prior to use. *n*-Butyllithium solutions were titrated against diphenylacetic acid to obtain accurate molarity. 4 Å molecular sieves were oven dried prior to use.

Reactions were monitored by thin layer chromatography (TLC) using Merck silica gel 60 covered aluminium-backed plates F254. TLC plates were visualised under UV light and stained using potassium permanganate solution or acidic ethanolic anisaldehyde solution. Flash column chromatography was performed with silica gel (Geduran Si 60 35–70 µm) as solid support.

IR spectra were recorded using a Shimadzu FT IR-8400S ATR instrument (Shimadzu UK, Milton Keynes, UK). The IR spectrum of each compound (solid or liquid) was acquired directly on a thin layer at ambient temperature.

NMR spectra were recorded on Bruker Avance III 400 MHz and 500 MHz spectrometers (Bruker UK, Coventry, UK) at ambient temperature. ^1^H NMR chemical shifts are reported in ppm relative to CHCl_3_ (7.26) or CDCl_2_H (5.32) on the δ scale followed by integration, multiplicity (s = singlet, d = doublet, t = triplet, q = quartet, m = multiplet, br. = broad, app. = apparent or a combination of these) and coupling constant(s) J (Hz). ^13^C NMR spectra were recorded at 101 MHz and 126 MHz and chemical shifts are reported in ppm relative to CDCl_3_ (77.16) or CD_2_Cl_2_ (54.00) on the δ scale.

High resolution mass spectra (HRMS) were recorded by the University of Glasgow mass spectrometry service using a JEOL MStation JMS-700 instrument [positive electron impact ionisation (EI^+^)] (JEOL Ltd., Tokyo, Japan) or a Bruker micrOTOF-Q instrument [positive ion electrospray (ESI^+^)] (Bruker UK, Coventry, UK).

Optical rotations were recorded with an error of ≤ ±0.1 using an automatic polarimeter Autopol V (Rudolph Research Analytical, Hackettstown, USA) and melting points were recorded with an Electrothermal IA 9100 apparatus (Cole-Parmer, Stone, UK).

*(1′S,3′R,9′S,11′R,15′Z)-4′-Methyl-2′,8′,12′-trioxaspiro(1,3-dioxolane-2,14′-tricyclo-[9.6.0.0^3,9^]heptadecane)-4′,15′-dien-6′-one* (**7**)

To a solution of the bis-enone **1** (870 mg, 3.13 mmol) in dichloromethane (63 mL) at −60 °C was added, 1,2-bis(trimethylsiloxy)ethane (0.81 mL, 3.3 mmol) and trimethylsilyl trifluoromethanesulfonate (0.06 mL, 0.3 mmol). The solution was allowed to warm to −25 °C and stirred for 16 h. The reaction mixture was diluted with diethyl ether (252 mL) and the mixture was washed with sat. aq. ammonium chloride (2 × 100 mL) and brine (100 mL). The organic phase was dried (MgSO_4_) and concentrated under reduced pressure to afford the acetal **7** (960 mg) as a brown oil. The unpurified acetal was used directly in the next reaction. For analytical purposes, a sample was purified by flash column chromatography (5–30% ethyl acetate in pet. ether) to afford **7** as a colourless solid. M.p. 145–146 °C; R*_f_* = 0.43 (60% ethyl acetate in pet. ether); [α]_D_ +22.6 (*c* = 0.50 in CHCl_3_, 29 °C); ν*_max_* 3028, 2953, 2932, 2884, 1655, 1443, 1348, 1115, 1086, 1074, 943, 893, 768 cm^−1^; ^1^H NMR (500 MHz, CDCl_3_) δ 5.90 (1H, dq, *J* = 1.2, 1.2 Hz), 5.82 (1H, ddd, *J* = 11.6, 9.5, 7.5 Hz), 5.63 (1H, d, *J* = 11.6 Hz), 4.23 (1H, d, *J* = 18.3 Hz), 4.13 (1H, d, *J* = 18.3 Hz), 3.94–3.83 (4H, m), 3.67 (1H, ddq, *J* = 9.0, 1.2, 1.2 Hz), 3.61 (1H, d, *J* = 12.7 Hz), 3.50 (1H, d, *J* = 12.7 Hz), 3.47 (1H, ddd, *J* = 11.7, 9.3, 4.4 Hz), 3.33 (1H, ddd, *J* = 9.3, 5.8, 4.0 Hz), 3.30 (1H, ddd, *J* = 11.7, 9.0, 4.7 Hz), 2.89 (1H, ddd, *J* = 13.5, 9.5, 4.0 Hz), 2.56 (1H, ddd, *J* = 13.5, 7.5, 5.8 Hz), 2.36 (1H, ddd, *J* = 12.1, 4.7, 4.4 Hz), 1.97 (3H, dd, *J* = 1.2, 1.2 Hz), 1.61 (1H, ddd, *J* = 12.1, 11.7, 11.7 Hz); ^13^C NMR (101 MHz, CDCl_3_) δ 201.4, 155.1, 133.9, 130.3, 127.1, 108.0, 82.8, 82.5, 78.1, 77.6, 77.4, 74.2, 64.8, 64.6, 38.3, 30.1, 22.9; HRMS (ESI^+^) [C_17_H_22_O_6_Na]^+^ found 345.1299, [M + Na]^+^ calcld. 345.1309.

*(1′S,3′R,9′S,11′R,15′Z)-4′-Methyl-2′,8′,12′-trioxaspiro(1,3-dioxolane-2,14′-tricyclo-[9.6.0.0^3,9^]heptadecane)-4′,6′,15′-trien-6′-yl prop-2-en-1-yl carbonate* (**8**)

To a solution of the acetal **7** in THF (57 mL) was added, allyl chloroformate (1.58 mL, 14.9 mmol). The resultant solution was cooled to −78 °C and sodium bis(trimethylsilyl)amide (3.0 mL of a 2.0 M solution in THF, 6.0 mmol) was added dropwise. The reaction mixture was stirred for 4 h, diluted with 5% aq. potassium dihydrogen phosphate (240 mL) and the mixture was extracted with diethyl ether (3 × 60 mL). The combined organic extracts were washed with brine (3 × 60 mL), dried (MgSO_4_) and concentrated under reduced pressure. The residue was dissolved in toluene (100 mL) and then concentrated under reduced pressure and the procedure was repeated. Residual material was purified by flash column chromatography (5–40% diethyl ether in pet. ether) to give the allylic carbonate **8** (610 mg, 48% over 2 steps) as a colourless oil. R*_f_* = 0.57 (60% ethyl acetate in pet. ether); [α]_D_ +96.1 (*c* = 1.00 in C_6_H_6_, 29 °C); ν*_max_* 2951, 2891, 1759, 1663, 1451, 1364, 1246, 1225, 1198, 1126, 1090, 1055, 995, 947, 770 cm^−1^; ^1^H NMR (500 MHz, CD_2_Cl_2_) δ 6.62 (1H, d, *J* = 1.6 Hz), 5.96 (1H, ddt, *J* = 17.3, 10.5, 5.7 Hz), 5.86 (1H, ddd, *J* = 11.5, 9.6, 7.5 Hz), 5.67 (1H, d, *J* = 11.5 Hz), 5.54 (1H, dqd, *J* = 1.6, 1.6, 1.3 Hz), 5.37 (1H, ddt, *J* = 17.3, 1.4, 1.4 Hz), 5.29 (1H, dddd, *J* = 10.5, 1.4, 1.4, 1.4 Hz), 4.64 (2H, ddd, *J* = 5.7, 1.4, 1.4 Hz), 3.95–3.87 (4H, m), 3.74 (1H, ddq, *J* = 7.9, 1.3, 1.3 Hz), 3.65 (1H, ddd, *J* = 11.4, 7.9, 4.9 Hz), 3.63 (1H, d, *J* = 12.8 Hz), 3.54 (1H, d, *J* = 12.8 Hz), 3.52 (1H, ddd, *J* = 11.8, 9.4, 4.4 Hz), 3.32 (1H, ddd, *J* = 9.4, 5.7, 4.1 Hz), 2.92 (1H, ddd, *J* = 13.4, 9.6, 4.1 Hz), 2.56 (1H, ddd, *J* = 13.4, 7.5, 5.7 Hz), 2.49 (1H, ddd, *J* = 12.1, 4.9, 4.4 Hz), 1.89 (3H, dd, *J* = 1.6, 1.3 Hz), 1.67 (1H, ddd, *J* = 12.1, 11.8, 11.4 Hz); ^13^C NMR (126 MHz, CD_2_Cl_2_) δ 155.0, 141.2, 138.5, 135.0, 134.2, 132.0, 130.7, 119.3, 118.8, 108.4, 81.8, 80.1, 78.0, 77.9, 74.6, 69.5, 65.1, 65.0, 38.7, 30.4, 21.9; HRMS (ESI^+^) [C_21_H_26_O_8_Na]^+^ found 429.1507, [M + Na]^+^ calcld. 529.1520.

*(1′S,3′R,7′R,9′S,11′R,15′Z)-4′-Methyl-7′-(prop-2-en-1-yl)-2′,8′,12′-trioxaspiro(1,3-dioxolane-2,14′-tricyclo[9.6.0.0^3,9^]heptadecane)-4′,15′-dien-6′-one* (**10**)

A mixture of tetrakis(triphenylphosphine)palladium(0) (67 mg, 0.058 mmol, 5 mol%) and (4*R*)-*t*-butyl-2-[2-(diphenylphosphino)phenyl]-4,5-dihydroxazole (**9**) (56 mg, 0.14 mmol, 13 mol%) was dissolved in THF (20 mL). The solution was stirred for 45 min and a solution of the allylic carbonate **8** (471 mg, 1.16 mmol) in THF (38 mL) was added. The mixture was stirred at rt for a further 3.5 h and then adsorbed onto Celite. The crude material was dry loaded on to a column of silica gel and purified by flash column chromatography (5–20% ethyl acetate in pet. ether) to afford the enone **10** (398 mg, 95%, >20:1 *dr*) as a pale yellow oil. R*_f_* = 0.57 (60% ethyl acetate in pet. ether); [α]_D_ +15.0 (*c* = 0.50 in CHCl_3_, 30 °C); ν*_max_* 2949, 2888, 2855, 1657, 1443, 1279, 1111, 1088, 999, 949, 918 cm^−1^; ^1^H NMR (500 MHz, CDCl_3_) δ 5.89 (1H, dq, *J* = 1.1, 1.1 Hz), 5.87 (1H, ddd, *J* = 11.5, 9.6, 7.5 Hz), 5.78 (1H, dddd, *J* = 17.0, 10.2, 6.8, 6.8 Hz), 5.70 (1H, d, *J* = 11.5 Hz), 5.12–5.00 (2H, m), 4.17 (1H, dd, *J* = 7.7, 4.4 Hz), 3.98–3.89 (4H, m), 3.68 (1H, ddq, *J* = 8.9, 1.1, 1.1 Hz), 3.67 (1H, d, *J* = 12.7 Hz), 3.55 (1H, d, *J* = 12.7 Hz), 3.50 (1H, ddd, *J* = 11.7, 9.3, 4.4 Hz), 3.40 (1H, ddd, *J* = 9.3, 5.5, 4.1 Hz), 3.33 (1H, ddd, *J* = 11.3, 8.9, 4.7 Hz), 2.97 (1H, ddd, *J* = 13.6, 9.6, 4.1 Hz), 2.59 (1H, ddd, *J* = 13.6, 7.5, 5.5 Hz), 2.49 (1H, ddd, *J* = 14.3, 6.8, 4.4 Hz), 2.41 (1H, ddd, *J* = 12.2, 4.7, 4.4 Hz), 2.36 (1H, ddd, *J* = 14.3, 7.7, 6.8 Hz), 2.01 (3H, dd, *J* = 1.1, 1.1 Hz), 1.70 (1H, ddd, *J* = 12.2, 11.7, 11.3 Hz); ^13^C NMR (126 MHz, CDCl_3_) δ 203.6, 153.7, 134.0, 133.5, 130.4, 126.7, 117.7, 107.9, 86.5, 82.5, 82.5, 77.6, 77.0, 74.2, 64.8, 64.6, 38.2, 38.1, 30.1, 22.7; HRMS (ESI^+^) [C_20_H_26_O_6_Na]^+^ found 385.1606, [M + Na]^+^ calcld. 385.1622.

*(1′S,3′R,4′S,7′R,9′S,11′R,15′Z)-4′-Methyl-7′-(prop-2-en-1-yl)-2′,8′,12′-trioxaspiro(1,3-dioxolane-2,14′-tricyclo[9.6.0.0^3^^,^⁹]heptadecan)-15′-en-6′-one* (**11a**) and *(1′S,3′R,4′R,7′R,9′S,11′R,15′Z)-4′-Methyl-7′-(prop-2-en-1-yl)-2′,8′,12′-trioxaspiro(1,3-dioxolane-2,14′-tricyclo[9.6.0.0^3,9^]heptadecan)-15′-en-6′-one* (**11b**)

(*a*) *Conjugate reduction of* **10** *with of ‘hot’ Stryker’s reagent*

To 1,2-bis(diphenylphosphino)benzene (491 mg, 1.10 mmol) and copper(II) acetate hydrate (220 mg, 1.10 mmol) was added degassed toluene (19 mL). The resultant mixture was stirred vigorously and sparged continuously with argon for 15 min. The mixture was then sonicated for a further 30 min, at which point a pale blue suspension had formed. Tetramethyldisiloxane (0.80 mL, 4.4 mmol) was added to the mixture and it was stirred vigorously for a further 30 min, at which point the suspension developed a green colour. After refrigeration for 16 h, a red stock solution (0.055 M) had formed.

To the enone **10** (18 mg, 50 µmol) was added ‘hot’ Stryker’s reagent (2.0 mL of a 0.055 M solution in toluene, 0.11 mmol). The solution was stirred at 0 °C for 48 h and then concentrated under reduced pressure. The residue was dissolved in THF (5 mL) and sat. aq. potassium fluoride (2 mL) was added. The biphasic mixture was stirred vigorously for 16 h and then diluted with water (10 mL). The mixture was extracted with diethyl ether (3 × 5 mL) and the combined organic extracts were dried (MgSO_4_) and concentrated under reduced pressure. The residue was purified by flash column chromatography (10–20% ethyl acetate in pet. ether) to afford a diastereomeric mixture of the ketones **11a** and **11b** (12 mg, 66%, 9:1 **a**:**b**) as a colourless film.

(*b*) *Conjugate reduction of* **10** *with lithium n-butyl(diisobutyl)aluminium hydride*

To a solution of 2,6-diphenylphenol (380 mg, 1.50 mmol) in toluene (2 mL) at 0 °C was added dropwise trimethylaluminium (0.25 mL of a 2.0 M solution in toluene, 0.50 mmol) and the mixture was stirred for 10 min. After gas evolution had subsided, a solution of the enone **10** (145 mg, 0.400 mmol) in toluene (1 mL) was added and the resultant solution cooled to −78 °C and stirred for 10 min.

To a solution of diisobutylaluminium hydride (0.60 mL of a 1.0 M solution in heptane, 0.60 mmol) in THF (2 mL) at 0 °C was added dropwise *n*-butyllithium (0.26 mL of a 2.33 M solution in hexane, 0.60 mmol) and solution was stirred for 10 min. The solution was added to the aforementioned toluene solution of the enone **10** and aluminium tris(2,6-diphenylphenoxide) at −78 °C and the mixture was stirred for 2 h at this temperature. The mixture was then diluted with diethyl ether (50 mL) and sat. aq. Rochelle salt (50 mL) was added and the mixture was stirred vigorously for 16 h. The phases were separated and the organic phase was washed with water (25 mL) and brine (25 mL), then dried (MgSO_4_) and concentrated under reduced pressure. The residue was purified by flash column chromatography (2.5–20% ethyl acetate in pentane) to afford the ketone **11b** (57 mg, 39%) as a colourless solid and the diketone (17 mg, 12%), resulting from deacetalation of the ketone **11b**, as a colourless film. **11a** R*_f_* = 0.20 (20% ethyl acetate in pet. ether); [α]_D_ +117.5 (*c* = 1.00 in CHCl_3_, 27 °C); ν*_max_* 2080, 3028, 2959, 2934, 2916, 2876, 1713, 1643, 1260, 1088, 1059, 949, 912, 891, 802, 769 cm^−1^; ^1^H NMR (500 MHz, CDCl_3_) δ 5.88 (1H, ddd, *J* = 11.6, 9.5, 7.5 Hz), 5.79 (1H, dddd, *J* = 17.1, 10.3, 6.9, 6.9 Hz), 5.67 (1H, d, *J* = 11.6 Hz), 5.11–5.04 (2H, m), 3.98–3.89 (4H, m), 3.80 (1H, dd, *J* = 7.9, 5.0 Hz), 3.66 (1H, d, *J* = 12.7 Hz), 3.54 (1H, d, *J* = 12.7 Hz), 3.46 (1H, ddd, *J* = 11.6, 9.3, 4.4 Hz), 3.28 (1H, ddd, *J* = 9.3, 5.8, 4.1 Hz), 2.96 (1H, ddd, *J* = 11.1, 9.3, 4.6 Hz), 2.89 (1H, ddd, *J* = 13.4, 9.5, 4.1 Hz), 2.88–2.78 (2H, m), 2.57 (1H, ddd, *J* = 13.4, 7.5, 5.8 Hz), 2.41–2.25 (3H, m), 2.12 (1H, dd, *J* = 11.5, 1.9 Hz), 1.69–1.57 (1H, m), 1.63 (1H, ddd, *J* = 12.4, 11.6, 11.1 Hz), 1.13 (3H, d, *J* = 6.6 Hz); ^13^C NMR (126 MHz, CDCl_3_) δ 215.2, 133.6, 133.1, 130.7, 118.0, 108.0, 86.5, 85.7, 81.6, 79.8, 77.9, 74.2, 64.8, 64.5, 45.1, 38.8, 37.2, 35.7, 30.1, 20.3; HRMS (ESI^+^) [C_20_H_28_O_7_Na]^+^ found 387.1766, [M + Na]^+^ calcld. 387.1778. **11b** M.p. 108–110 °C; R*_f_* = 0.25 (20% ethyl acetate in pet. ether); [α]_D_ +87.1 (*c* = 2.00 in CHCl_3_, 29 °C); ν*_max_* 2957, 2936, 2884, 2855, 1711, 1641, 1288, 1090, 1061, 1042, 995, 949, 920, 770 cm^−1^; ^1^H NMR (500 MHz, CDCl_3_) δ 5.87 (1H, ddd, *J* = 11.5, 9.6, 7.4 Hz), 5.80 (1H, dddd, *J* = 17.1, 10.2, 6.9, 6.9 Hz), 5.70 (1H, d, *J* = 11.5 Hz), 5.11–5.02 (2H, m), 3.98–3.90 (4H, m), 3.71 (1H, dd, *J* = 8.3, 4.2 Hz), 3.67 (1H, d, *J* = 12.7 Hz), 3.54 (1H, d, *J* = 12.7 Hz), 3.41–3.34 (3H, m), 3.20 (1H, ddd, *J* = 11.1, 9.6, 5.0 Hz), 3.03–2.94 (1H, m), 3.00 (1H, dd, *J* = 11.7, 2.2 Hz), 2.50 (1H, ddd, *J* = 13.5, 7.4, 4.4 Hz), 2.42 (1H, ddddd, *J* = 13.4, 6.9, 4.2, 1.3, 1.3 Hz), 2.37 (1H, ddd, *J* = 12.1, 5.0, 4.2 Hz), 2.34–2.25 (3H, m), 1.65 (1H, ddd, *J* = 12.1, 11.3, 11.1 Hz), 0.86 (3H, d, *J* = 7.0 Hz); ^13^C NMR (126 MHz, CDCl_3_) δ 215.5, 133.9, 133.6, 130.9, 117.8, 107.8, 87.1, 83.1, 81.7, 77.7, 74.9, 74.4, 64.8, 64.6, 44.3, 38.5, 37.5, 31.9, 30.1, 12.3; HRMS (ESI^+^) [C_20_H_28_O_6_Na]^+^ found 387.1765, [M + Na]^+^ calcld. 387.1778.

*(1′S,3′R,4′S,5′S,7′R,9′S,11′R,15′Z)-5′-Hydroxy-4′-methyl-7′-(prop-2-en-1-yl)-2′,8′,12′-trioxaspiro(1,3-dioxolane-2,14′-tricyclo[9.6.0.0^3,9^]heptadecan)-15′-en-6′-one* (**13**)

To a solution of the ketones **11a** and **11b** (18 mg of a 4:1 mixture, 50 µmol) in THF (2.9 mL) at −78 °C, a solution of potassium bis(trimethylsilyl)amide (0.10 mL of a 0.50 M solution in toluene, 50 µmol) was added and the mixture was stirred for 1 h. A solution of the oxaziridine **12** (14 mg, 50 µmol) in THF (1 mL) was then added to the mixture dropwise and the resulting mixture was stirred for 1 h. The reaction mixture was diluted with sat. aq. sodium thiosulfate (20 mL) and the extracted with diethyl ether (3 × 10 mL). The combined organic extracts were washed with sat. aq. ammonium chloride (10 mL) and brine (2 × 10 mL), dried (MgSO_4_) and concentrated under reduced pressure. The residue was purified by flash column chromatography (10–30% diethyl ether in pet. ether) to give the α-hydroxyketone **13** (8 mg, 43%) and recovered starting material **11a** (2 mg, 11%) as colourless oils. R*_f_* = 0.73 (60% ethyl acetate in pet. ether); [α]_D_ +75.8 (*c* = 0.60 in CHCl_3_, 26 °C); ν*_max_* 3474, 2926, 2874, 2855, 1713, 1643, 1283, 1096, 1047, 949, 922, 770 cm^−1^; ^1^H NMR (400 MHz, CDCl_3_) δ 5.88 (1H, ddd, *J* = 11.5, 9.5, 7.5 Hz), 5.78 (1H, ddd, *J* = 16.9, 9.8, 7.0 Hz), 5.67 (1H, d, *J* = 11.5 Hz), 5.13–5.06 (2H, m), 4.30 (1H, dd, *J* = 11.2, 6.4 Hz), 4.08 (1H, dd, *J* = 7.7, 5.0 Hz), 3.97–3.91 (4H, m), 3.66 (1H, d, *J* = 12.7 Hz), 3.54 (1H, d, *J* = 12.7 Hz), 3.46 (1H, ddd, *J* = 11.7, 9.3, 4.5 Hz), 3.33 (1H, d, *J* = 6.4 Hz), 3.28 (1H, ddd, *J* = 9.3, 5.8, 4.2 Hz), 3.01–2.94 (2H, m), 2.90 (1H, ddd, *J* = 13.5, 9.5, 4.2 Hz), 2.58 (1H, ddd, *J* = 13.5, 7.5, 5.8 Hz), 2.48–2.32 (3H, m), 1.63 (1H, app. q, *J* = 11.7 Hz), 1.54–1.44 (1H, m), 1.28 (3H, d, *J* = 6.3 Hz); ^13^C NMR (101 MHz, CDCl_3_) δ 215.5, 133.6, 132.4, 130.6, 118.7, 108.1, 85.5, 83.6, 81.7, 79.8, 77.7, 75.1, 74.2, 64.8, 64.5, 43.2, 38.6, 37.9, 30.1, 15.3; HRMS (ESI^+^) [C_20_H_28_O_7_Na]^+^ found 403.1715, [M + Na]^+^ calcld. 403.1727.

*(1′S,3′R,4′S,5′S,6′S,7′R,9′S,11′R,15′Z)-4′-Methyl-7′-(prop-2-en-1-yl)-2′,8′,12′-trioxaspiro(1,3-dioxolane-2,14′-tricyclo[9.6.0.0^3,9^]heptadecan)-15′-ene-5′,6′-diol* (**14**)

To a solution of α-hydroxyketone **13** (31 mg, 81 µmol) in dichloromethane (2.9 mL) at −78°C, diisobutylaluminium hydride (0.32 mL of a 1.0 M solution in dichloromethane, 0.32 mmol) was added and the resultant solution stirred for 2 h. Further diisobutylaluminium hydride (0.32 mL of a 1.0 M solution in dichloromethane, 0.32 mmol) was added and the mixture was stirred for a further 2 h. The reaction mixture was diluted with sat. aq. Rochelle salt (20 mL) and ethyl acetate (20 mL) and stirred vigorously for 1 h. The phases were separated and the organic phase was washed with brine (2 × 10 mL), dried (MgSO_4_) and concentrated under reduced pressure. The residue was purified by flash column chromatography (20–100% diethyl ether in pet. ether) to afford the diol **14** (20 mg, 64%) and starting material **13** (6 mg, 19%) as colourless films. R*_f_* = 0.30 (60% ethyl acetate in pet. ether); [α]_D_ +18.4 (*c* = 0.50 in CHCl_3_, 27 °C); ν*_max_* 3447, 2926, 2874, 1647, 1456, 1285, 1090, 1042, 1013, 916, 770 cm^−1^; ^1^H NMR (400 MHz, CDCl_3_) δ 5.90 (1H, ddd, *J* = 11.4, 9.6, 7.6 Hz), 5.85 (1H, dddd, *J* = 17.1, 10.2, 6.9, 6.9 Hz), 5.67 (1H, d, *J* = 11.4 Hz), 5.17–5.04 (2H, m), 3.94 (4H, m), 3.86 (1H br s), 3.71 (1H, d, *J* = 9.4 Hz), 3.65 (1H, d, *J* = 12.7 Hz), 3.56 (1H, ddd, *J* = 7.6, 5.5, 5.5 Hz), 3.55 (1H, d, *J* = 12.7), 3.48 (1H, ddd, *J* = 11.6, 9.3, 4.4 Hz), 3.37 (1H, ddd, *J* = 11.4, 9.3, 4.6 Hz), 3.22 (1H, ddd, *J* = 9.3, 5.9, 4.1 Hz), 2.88 (1H, ddd, *J* = 13.6, 9.6, 4.1 Hz), 2.72 (1H, dd, *J* = 9.3, 9.0 Hz), 2.59 (1H, ddd, *J* = 13.6, 7.6, 5.9 Hz), 2.39–2.24 (3H, m), 2.21 (1H, br s), 2.12 (1H, ddq, 9.4, 9.0, 6.7 Hz), 1.94 (1H, br s), 1.48 (1H, ddd, *J* = 12.1, 11.6, 11.4 Hz), 1.15 (3H, d, *J* = 6.7 Hz); ^13^C NMR (101 MHz, CDCl_3_) δ 134.8, 133.4, 130.8, 117.5, 108.2, 84.8, 82.7, 81.7, 78.3, 78.2, 77.7, 74.1, 73.4, 64.8, 64.5, 39.4, 39.0, 38.0, 30.3, 15.4; HRMS (ESI^+^) [C_20_H_30_O_7_Na]^+^ found 405.1871, [M + Na]^+^ calcld. 405.1884.

*(1′S,3′R,4′S,5′S,6′R,7′R,9′S,11′R,15′Z)-4′-Methyl-5′-[(naphthalen-2-yl)methoxy]-7′-(prop-2-en-1-yl)-2′,8′,12′-trioxaspiro(1,3-dioxolane-2,14′-tricyclo[9.6.0.0^3,9^]heptadecane)-15′-en-6′-ol* (**15**)

To a solution of diol **14** (20 mg, 52 µmol) in methanol (5.2 mL), dibutyltin oxide (14 mg, 58 µmol) was added. The resultant suspension was stirred at reflux for 2 h to form a colourless solution. The mixture was concentrated under reduced pressure and dried by azeotropic distillation with toluene. The mixture was redissolved in DMF (5.2 mL) and 2-(bromomethyl)naphthalene (14 mg, 62 µmol) and caesium fluoride (9 mg, 0.06 mmol) were added. The reaction mixture was stirred for 16 h and then quenched with water carefully. The mixture was extracted with diethyl ether (3 × 10 mL) and the combined organic extracts were washed with brine (3 × 10 mL), dried (MgSO_4_) and concentrated under reduced pressure. The residue was purified by flash column chromatography (20–100% diethyl ether in pet. ether) to afford the naphthyl ether **15** (17 mg, 62%) and recovered diol **14** (4 mg, 20%) as colourless films. R*_f_* = 0.39 (80% diethyl ether in pet. ether); [α]_D_ +14.8 (*c* = 1.00 in CHCl_3_, 27 °C); ν*_max_* 3466, 2928, 2874, 2853, 1643, 1456, 1090, 949, 916, 1051, 818, 752 cm^−1^; ^1^H NMR (400 MHz, CDCl_3_) δ 7.87–7.80 (3H, m), 7.76 (1H, s), 7.52–7.44 (3H, m), 5.91 (1H, ddd, *J* = 11.5, 9.6, 7.5 Hz), 5.83 (1H, dddd, *J* = 17.2, 10.3, 6.6, 6.6 Hz), 5.69 (1H, d, *J* = 11.5 Hz), 5.13–5.02 (2H, m), 4.74 (1H, d, *J* = 11.3 Hz), 4.65 (1H, d, *J* = 11.3 Hz), 3.98–3.92 (4H, m), 3.90 (1H, dd, *J* = 5.0, 2.2 Hz), 3.66 (1H, d, *J* = 12.7 Hz), 3.59 (1H, ddd, *J* = 7.9, 5.0, 5.0 Hz), 3.55 (1H, d, *J* = 12.7), 3.53 (1H, dd, *J* = 8.8, 2.2 Hz), 3.50 (1H, ddd, *J* = 11.6, 9.5, 4.3 Hz), 3.49 (1H, ddd, *J* = 11.6, 9.4, 4.5 Hz), 3.24 (1H, ddd, *J* = 9.4, 5.7, 4.1 Hz), 2.92 (1H, ddd, *J* = 13.7, 9.6, 4.1 Hz), 2.78 (1H, dd, *J* = 9.5, 7.7 Hz), 2.59 (1H, ddd, *J* = 13.7, 7.5, 5.7 Hz), 2.39–2.19 (4H, m), 1.46 (1H, ddd, *J* = 11.6, 11.6, 11.6 Hz), 1.18 (3H, d, *J* = 6.9 Hz); ^13^C NMR (101 MHz, CDCl_3_) δ 135.4, 134.9, 133.5, 133.3, 133.2, 130.9, 128.4, 128.0, 127.9, 127.1, 126.4, 126.3, 126.2, 117.2, 108.1, 85.4, 82.0, 81.8, 81.8, 78.2, 77.3, 74.3, 74.1, 72.6, 64.8, 64.5, 39.0, 39.0, 36.8, 30.3, 16.4; HRMS (ESI^+^) [C_31_H_38_O_7_Na]^+^ found 545.2497, [M + Na]^+^ calcld. 545.2510.

*(1′S,3′R,6′S,7′R,9′S,11′R,15′Z)-4′-Methyl-7′-(prop-2-en-1-yl)-2′,8′,12′-trioxaspiro(1,3-dioxolane-2,14′-tricyclo[9.6.0.0^3,9^]heptadecane)-4′,15′-dien-6′-ol* (**16**)

To a solution of the enone **10** (292 mg, 0.806 mmol) in methanol (16 mL), cerium(III) chloride heptahydrate (361 mg, 0.969 mmol) was added. The solution was cooled to −78 °C and sodium borohydride (37 mg, 0.97 mmol) was added and the mixture was stirred for 2 h. The mixture was diluted with ethyl acetate (80 mL) and washed with sat. aq. ammonium chloride (3 × 40 mL). The organic phase was dried (MgSO_4_) and concentrated under reduced pressure to afford the crude allylic alcohol **16** (294 mg) as a colourless gum. The residue was used directly in the next reaction without purification. For analytical purposes, a sample of the product was purified by flash column chromatography (25–75% diethyl ether in pentane) to afford the allylic alcohol **16** as a colourless gum. R*_f_* = 0.68 (60% ethyl acetate in pet. ether); [α]_D_ +13.5 (*c* = 1.00 in CHCl_3_, 21 °C); ν*_max_* 2994, 2942, 2880, 1701, 1641, 1377, 1101, 1087, 984, 959, 854 cm^−1^; ^1^H NMR (400 MHz, CDCl_3_) δ 5.91 (1H, dddd, *J* = 17.1, 10.3, 6.9, 6.9 Hz), 5.88 (1H, ddd, *J* = 11.4, 9.6, 7.6 Hz), 5.69 (1H, d, *J* = 11.4 Hz), 5.47 (1H, ddq, *J* = 2.2, 1.6, 1.6 Hz), 5.17–5.00 (2H, m), 4.05 (1H, dd, *J* = 8.8, 2.2 Hz), 3.99–3.89 (4H, m), 3.66 (1H, ddq, *J* = 9.0, 1.6, 1.6 Hz), 3.66 (1H, d, *J* = 12.7 Hz), 3.55 (1H, d, *J* = 12.7 Hz), 3.45 (1H, ddd, *J* = 11.7, 9.2, 4.4 Hz), 3.35–3.23 (1H, m), 3.29 (1H, ddd, *J* = 11.2, 9.0, 4.7 Hz), 3.26 (1H, ddd, *J* = 9.2, 5.5, 4.1 Hz), 2.94 (1H, ddd, *J* = 13.6, 9.6, 4.1 Hz), 2.55 (1H, ddd, *J* = 13.6, 7.6, 5.5 Hz), 2.49 (1H, ddddd, *J* = 14.6, 6.9, 3.5, 1.3, 1.3 Hz), 2.30 (1H, ddd, *J* = 12.1, 4.7, 4.4 Hz), 2.20 (1H, ddddd, *J* = 14.6, 8.2, 6.9, 1.3, 1.3 Hz), 1.78 (3H, dd, *J* = 1.6, 1.6 Hz), 1.58 (1H, ddd, *J* = 12.1, 11.7, 11.2 Hz); ^13^C NMR (101 MHz, CDCl_3_) δ 137.7, 135.3, 133.6, 130.8, 129.0, 117.0, 108.0, 83.9, 82.2, 81.7, 78.2, 77.8, 74.2, 73.4, 64.7, 64.6, 38.9, 37.8, 30.1, 21.9; HRMS (ESI^+^) [C_20_H_28_O_6_Na]^+^ found 387.1771, [M + Na]^+^ calcld. 387.1778

*(1′S,3′S,4′R,6′R,7′R,8′R,10′S,12′R,16′Z)-4′-Methyl-8′-(prop-2-en-1-yl)-2′,5′,9′,13′-tetraoxaspiro(1,3-dioxolane-2,15′-tetracyclo[10.6.0.0^3,10^.0^4,6^]octadecane)-16′-en-7′-ol* (**17**)

To a stirred solution of allylic alcohol **16** (294 mg) in benzene (41 mL), vanadyl acetylacetonate (42 mg, 0.16 mmol, >20 mol%) was added. A teal solution formed after 10 min and then *t*-butyl hydroperoxide (0.32 mL of a 5.0 M in decane, 1.6 mmol) was added to form a red solution. The solution was stirred at 60 °C for 2 h during which time the solution turned yellow. The reaction mixture was diluted with diethyl ether (40 mL) and washed with sat. aq. sodium sulfite (2 × 40 mL), sat. aq. ammonium chloride / 10% aq. ammonium hydroxide (4:1, 2 × 40 mL) and brine (2 × 40 mL). The organic phase was dried (Na_2_SO_4_) and concentrated under reduced pressure to afford the epoxide **17** (290 mg) as a yellow oil. The residue was used directly in the next reaction without purification. For analytical purposes, a sample of the product was purified by flash column chromatography (25–75% diethyl ether in pentane) to afford the epoxide **17** as a colourless oil. R*_f_* = 0.39 (60% ethyl acetate in pet. ether); [α]_D_ +11.8 (*c* = 1.00 in CHCl_3_, 22 °C); ν*_max_* 3457, 2924, 2872, 2855, 1641, 1443, 1281, 1111, 1086, 1055, 1018, 882, 770 cm^−1^; ^1^H NMR (400 MHz, CD_2_Cl_2_) δ 5.93–5.81 (2H, m), 5.66 (1H, d, *J* = 11.5 Hz), 5.12–5.02 (2H, m), 3.95–3.86 (4H, m), 3.75 (1H, ddd, *J* = 8.9, 8.2, 1.0 Hz), 3.59 (1H, d, *J* = 12.7 Hz), 3.52 (1H, d, *J* = 12.7 Hz), 3.46 (1H, ddd, *J* = 11.6, 9.1, 4.5 Hz), 3.29 (1H, d, *J* = 9.1 Hz), 3.28–3.19 (2H, m), 3.15 (1H, ddd, *J* = 11.5, 9.1, 4.7 Hz), 3.08 (1H, d, *J* = 1.0 Hz), 2.86 (1H, ddd, *J* = 13.7, 9.6, 4.0 Hz), 2.60 (1H, ddd, *J* = 13.7, 6.9, 6.9 Hz), 2.53 (1H, ddd, *J* = 14.5, 6.9, 6.9 Hz), 2.26 (1H, ddd, *J* = 12.0, 4.7, 4.5 Hz), 2.12 (1H, ddd, *J* = 14.5, 7.7, 7.7 Hz), 2.02 (1H, d, *J* = 8.2 Hz), 1.42 (1H, ddd, *J* = 12.0, 11.6, 11.5 Hz), 1.40 (3H, s); ^13^C NMR (101 MHz, CD_2_Cl_2_) δ 135.8, 134.1, 130.7, 117.1, 108.5, 82.7, 82.2, 80.5, 78.0, 75.7, 74.4, 72.9, 67.2, 65.1, 64.9, 61.5, 38.9, 37.8, 30.5, 21.3; HRMS (EI^+^) [C_20_H_28_O_7_]^+^ found 380.1831, [M]^+^ calcld. 380.1835.

*[(1′S,3′S,4′R,6′R,7′R,8′R,10′S,12′R,16′Z)-4′-Methyl-8′-(prop-2-en-1-yl)-2′,5′,9′,13′-tetraoxaspiro(1,3-dioxolane-2,15′-tetracyclo[10.6.0.0^3,10^.0^4,6^]octadecane)-16′-en-7′-yloxy]tris(propan-2-yl)silane* (**18**)

The reaction was performed two batches. To a solution of the epoxy alcohol **17** (145 mg) in dichloromethane (20 mL), 4-dimethylaminopyridine (244 mg, 2.00 mmol), triethylamine (1.12 mL, 8.0 mmol) and triisopropylsilyl trifluoromethanesufonate (1.61 mL, 6.0 mmol) were added. The mixture was stirred for 16 h and diluted with diethyl ether (80 mL). The mixture was washed with sat. aq. ammonium chloride (3 × 50 mL), dried (Na_2_SO_4_) and concentrated under reduced pressure. The residue was purified by flash column chromatography (5–15% diethyl ether in pet. ether). The batches were combined to give the silyl ether **18** (284 mg, 66% over 3 steps) as a colourless oil. R*_f_* = 0.51 (20% ethyl acetate in pet. ether); [α]_D_ +39.2 (*c* = 0.50 in CHCl_3_, 27 °C); ν*_max_* 2943, 2893, 2866, 1641, 1464, 1109, 1089, 883, 912, 718 cm^−1^; ^1^H NMR (400 MHz, CD_2_Cl_2_) δ 5.89 (1H, ddd, *J* = 11.5, 9.6, 7.5 Hz), 5.86 (1H, dddd, *J* = 17.1, 10.3, 6.8, 6.8 Hz), 5.66 (1H, d, *J* = 11.5 Hz), 5.09–4.98 (2H, m), 3.97 (1H, dd, *J* = 9.2, 1.0 Hz), 3.94–3.86 (4H, m), 3.59 (1H, d, *J* = 12.7 Hz), 3.52 (1H, d, *J* = 12.7 Hz), 3.45 (1H, ddd, *J* = 11.6, 9.2, 4.4 Hz), 3.29–3.25 (1H, m), 3.27 (1H, d, *J* = 9.2 Hz), 3.21 (1H, ddd, *J* = 9.2, 6.0, 4.0 Hz), 3.14 (1H, ddd, *J* = 11.5, 9.2, 4.7 Hz), 3.01 (1H, d, *J* = 1.0 Hz), 2.87 (1H, ddd, *J* = 13.5, 9.6, 4.0 Hz), 2.65–2.53 (2H, m), 2.25 (1H, ddd, *J* = 11.9, 4.7, 4.4 Hz), 2.02 (1H, ddddd, *J* = 14.4, 10.1, 6.8, 1.3, 1.3 Hz), 1.40 (1H, ddd, *J* = 11.9, 11.6, 11.5 Hz), 1.38 (3H, s), 1.16–1.02 (21H, m); ^13^C NMR (101 MHz, CD_2_Cl_2_) δ 136.4, 134.1, 130.7, 116.7, 108.5, 82.8, 82.3, 81.2, 78.1, 75.6, 74.4, 74.4, 67.4, 65.1, 64.9, 61.2, 38.9, 37.8, 30.6, 21.5, 18.6, 18.5, 13.6; HRMS (ESI^+^) [C_29_H_48_O_7_SiNa]^+^ found 559.3047, [M + Na]^+^ calcld. 559.3062.

*(1S,3R,5S,6S,7R,9S,11R,15Z)-5-Hydroxy-4-methylidene-7-(prop-2-en-1-yl)-6-{[tris(propan-2-yl)silyl]oxy}-2,8,12-trioxatricyclo[9.6.0.0^3,9^]heptadec-15-en-14-one* (**19**)

To a solution of 2,2,6,6-tetramethylpiperidine (0.05 mL, 0.3 mmol) in toluene (0.45 mL) at −10 °C, a solution of *n*-BuLi (0.5 mL of a 0.6 M solution in toluene and hexane, 3:2, 0.3 mmol) was added dropwise. The mixture was stirred for 30 min to produce an orange solution. Diethylaluminium chloride (0.30 mL of a 1.0 M solution in hexane, 0.30 mmol) was added dropwise and the mixture was stirred for a further 30 min resulting in the formation of a colourless solution. To the resultant solution of the complex was added a solution of the epoxide **18** (27 mg, 50 µmol) in toluene (0.70 mL). The reaction mixture was allowed to reach rt over 16 h and then diluted with diethyl ether (8 mL). Sat. aq. Rochelle salt (10 mL) was added and the mixture was stirred vigorously for 2 h. The phases were separated and the organic phase was washed with sat. aq. ammonium chloride (10 mL) and brine (10 mL), then dried (MgSO_4_) and concentrated under reduced pressure. The residue was purified by flash column chromatography (10–40% diethyl ether in pentane) to afford the allylic alcohol **19** (10 mg, 40%) as a yellow oil. R*_f_* = 0.60 (60% ethyl acetate in pet. ether); [α]_D_ +5.8 (*c* = 0.50 in CHCl_3_, 27 °C); ν*_max_* 3476, 2891, 2866, 1676, 1643, 1464, 1098, 1016, 997, 918, 883 cm^−1^; ^1^H NMR (400 MHz, CDCl_3_) δ 6.47 (1H, ddd, *J* = 12.4, 8.8, 7.6 Hz), 5.87 (1H, dddd, *J* = 17.1, 10.2, 6.9, 6.6 Hz), 5.86 (1H, dddd, *J* = 12.4, 1.6, 1.6, 1.1 Hz), 5.45 (1H, ddd, *J* = 1.3, 1.3, 1.3 Hz), 5.39 (1H, ddd, *J* = 1.3, 1.3, 1.3 Hz), 5.13–5.04 (2H, m), 4.51 (1H, dd, *J* = 17.8, 1.1 Hz), 4.47 (1H, ddd, *J* = 2.5, 1.3, 1.3 Hz), 4.20 (1H, d, *J* = 17.8 Hz), 3.80 (1H, dd, *J* = 6.7, 2.5 Hz), 3.68 (1H, ddd, *J* = 11.2, 9.6, 4.3 Hz), 3.64 (1H, ddd, *J* = 9.6, 1.3, 1.3 Hz), 3.55 (1H, ddd, *J* = 9.7, 6.7, 3.0 Hz), 3.41 (1H, ddd, *J* = 11.4, 9.1, 4.2 Hz), 3.32 (1H, ddd, *J* = 9.1, 9.1, 1.6 Hz), 2.70 (1H, dddd, *J* = 14.9, 7.6, 1.6, 1.6 Hz), 2.64 (1H, dddd, *J* = 14.9, 9.1, 8.8, 1.6 Hz), 2.46 (1H, ddddd, *J* = 14.4, 6.9, 3.0, 1.3, 1.3 Hz), 2.34 (1H, ddd, *J* = 11.6, 4.3, 4.2 Hz), 2.16 (1H, ddddd, *J* = 14.4, 9.7, 6.6, 1.3, 1.3 Hz), 1.65 (1H, ddd, *J* = 11.6, 11.4, 11.2 Hz), 1.15–1.03 (21H, m); ^13^C NMR (101 MHz, CDCl_3_) δ 203.7, 145.1, 138.1, 135.3, 129.0, 117.7, 117.0, 85.3, 83.1, 82.0, 78.7, 78.6, 77.4, 77.0, 76.3, 38.3, 38.2, 35.1, 18.3, 13.0; HRMS (ESI^+^) [C_27_H_44_O_6_SiNa]^+^ found 515.2775, [M + Na]^+^ calcld. 515.2799.

*(1′S,3′R,5′S,6′S,7′R,9′S,11′R,15′Z)-4′-Methylidene-7′-(prop-2-en-1-yl)-6′-{[tris(propan-2-yl)silyl]oxy}-2′,8′,12′-trioxaspiro(1,3-dioxolane-2,14′-tricyclo[9.6.0.0^3,9^]heptadecane)-15′-en-5′-ol* (**20**)

To a solution of epoxide **18** (43 mg, 80 µmol) in THF (4 mL), *p*-toluenesulfonic acid (69 mg, 0.40 mmol) and 4 Å molecular sieves (3 g) were added. The resultant mixture was stirred for 16 h and dried (4 Å molecular sieves) ethylene glycol (1 mL) was added. The reaction mixture was stirred for a further 24 h and diluted with sat. aq. sodium bicarbonate (25 mL). The organic phase was extracted with diethyl ether (3 × 10 mL) and the combined organic extracts were washed with brine (3 × 10 mL), dried (MgSO_4_) and concentrated under reduced pressure. The residue was purified by flash column chromatography (10–30% diethyl ether in pet. ether) to afford allylic alcohol **20** (17 mg, 40%) and starting epoxide **18** (11 mg, 26%) as colourless films. R*_f_* = 0.63 (60% ethyl acetate in pet. ether); [α]_D_ +32.4 (*c* = 1.00 in CHCl_3_, 27 °C); ν*_max_* 3476, 2941, 2866, 1643, 1464, 1090, 1018, 901, 883 cm^−1^; ^1^H NMR (400 MHz, CDCl_3_) δ 5.92 (1H, ddd, *J* = 11.4, 9.5, 7.5 Hz), 5.87 (1H, dddd, *J* = 17.1, 10.3, 7.1, 6.5 Hz), 5.68 (1H, d, *J* = 11.4 Hz), 5.45 (1H, ddd, *J* = 1.4, 1.4, 1.4 Hz), 5.35 (1H, ddd, *J* = 1.4, 1.4, 1.4 Hz), 5.12–5.02 (2H, m), 4.44 (1H, dddd, *J* = 4.1, 2.7, 1.4, 1.4 Hz), 3.98–3.91 (4H, m), 3.79 (1H, dd, *J* = 6.9, 2.7 Hz), 3.66 (1H, d, *J* = 12.8 Hz), 3.61 (1H, ddd, *J* = 9.6, 1.4, 1.4 Hz), 3.56 (1H, d, *J* = 12.8 Hz), 3.54–3.46 (3H, m), 3.38 (1H, ddd, *J* = 9.3, 5.5, 4.1 Hz), 2.96 (1H, ddd, *J* = 13.4, 9.5, 4.1 Hz), 2.62 (1H, ddd, *J* = 13.4, 7.5, 5.5 Hz), 2.45 (1H, ddddd, *J* = 14.5, 7.1, 2.8, 1.3, 1.3 Hz), 2.31 (1H, d, *J* = 4.1 Hz), 2.30 (1H, ddd, *J* = 11.7, 4.4, 4.4 Hz), 2.15 (1H, ddddd, *J* = 14.5, 9.6, 6.5, 1.4, 1.4 Hz), 1.53 (1H, app. dt, *J* = 11.7, 11.4 Hz), 1.13–1.04 (21H, m); ^13^C NMR (101 MHz, CDCl_3_) δ 145.6, 135.4, 133.5, 130.9, 117.0, 116.9, 108.0, 83.2, 82.0, 82.0, 78.6, 78.2, 77.8, 76.1, 74.1, 64.8, 64.6, 38.7, 38.3, 30.3, 18.3, 13.1; HRMS (ESI^+^) [C_29_H_48_O_7_SiNa]^+^ found 559.3041, [M + Na]^+^ calcld. 559.3062.

*(1′R,3′S,5′Z,10′R,12′S,14′R,19′S,20′S,21′S)-21′-Methyl-20′-[(naphthalen-2-yl)methoxy]-2′,9′,13′,18′-tetraoxaspiro[1,3-dioxolane-2,7′-tetracyclo(10.9.0.0^3,10^.0^14,19^]henicosane)-5′,15′-dien-17′-one* (**25**)

To a solution of alcohol **15** (8 mg, 0.02 mmol) in methanol (0.64 mL), the ruthenium complex **21** (3 mg, 0.03 mmol, 20 mol%) was added. The solution was stirred at reflux for 1 h and concentrated under reduced pressure. The residue was purified by flash column chromatography to afford an *E* / *Z* mixture of alcohol **22** (4 mg) as a colourless film. The residue was used directly in the next reaction without purification.

To a solution of (2*E*)-4-(prop-2-en-1-yloxy)but-2-enoic acid **23** (2 mg, 0.02 mmol) in dichloromethane (0.8 mL), *N*,*N*′-dicyclohexylcarbodiimide (3 mg, 0.02 mmol) and 4-dimethylaminopyridine (0.2 mg, 0.02 mmol) were added. The resultant suspension was added to **22** (4 mg) and the mixture stirred for 16 h. The mixture was diluted with diethyl ether (4.2 mL) and filtered. The filtrate was concentrated under reduced pressure and the residue was purified by flash column chromatography (5–60% diethyl ether in pet. ether) to give an isomeric mixture of esters **24** (2 mg) as a colourless film. The residue was used directly in the next reaction without purification.

To a solution of ester **24** (2 mg) in dichloromethane (16 mL), the ruthenium complex **21** (1 mg, 1 µmol) was added. The solution was stirred for 6 h at reflux and concentrated under reduced pressure. The residue was purified by flash column chromatography (0–25% diethyl ether in dichloromethane) to deliver the tetracyclic lactone **25** (1 mg, 12% over 3 steps) as a colourless film. R*_f_* = 0.23 (80% diethyl ether in pet. ether); [α]_D_ +48 (*c* = 0.08 in CHCl_3_, 36 °C); ν*_max_* 2922, 2851, 1734, 1636, 1559, 1119, 1042, 818 cm^−1^; ^1^H NMR (500 MHz, CDCl_3_) δ 7.88–7.81 (3H, m), 7.80 (1H, s), 7.54–7.42 (3H, m), 6.82 (1H, dd, *J* = 9.9, 1.6 Hz), 5.93 (1H, dd, *J* = 9.9, 2.5 Hz), 5.91 (1H, ddd, *J* = 11.4, 9.7, 7.4 Hz), 5.73 (1H, d, *J* = 11.4 Hz), 4.87 (1H, d, *J* = 11.5 Hz), 4.86 (1H, ddd, *J* = 10.5, 2.5, 1.6 Hz), 4.81 (1H, d, *J* = 11.5 Hz), 4.26 (1H, dd, *J* = 10.5, 0.7 Hz), 3.96 (4H, m), 3.90 (1H, ddd, *J* = 11.0, 9.3, 5.5 Hz), 3.83 (1H, dd, *J* = 2.6, 0.7 Hz), 3.67 (1H, d, *J* = 12.7 Hz), 3.54 (1H, d, *J* = 12.7 Hz), 3.43 (1H, ddd, *J* = 12.4, 9.1, 4.3 Hz), 3.23 (1H, ddd, *J* = 9.1, 7.2, 5.5 Hz), 2.97 (1H, ddd, *J* = 13.5, 9.7, 7.2 Hz), 2.93 (1H, dd, *J* = 9.3, 5.7 Hz), 2.54 (1H, ddd, *J* = 13.5, 7.4, 5.5 Hz), 2.33 (1H, ddd, *J* = 11.8, 5.5, 4.3 Hz), 2.27 (1H, qdd, *J* = 7.4, 5.7, 2.6 Hz), 1.47 (1H, ddd, *J* = 12.4, 11.8, 11.0 Hz), 1.18 (3H, d, *J* = 7.4 Hz); ^13^C NMR (126 MHz, CDCl_3_) δ 163.4, 149.9, 135.6, 133.8, 133.3, 133.1, 130.8, 128.3, 128.1, 127.9, 126.8, 126.3, 126.1, 126.1, 119.4, 107.9, 86.9, 82.9, 81.8, 81.0, 77.7, 75.4, 74.4, 73.0, 69.8, 64.8, 64.7, 39.9, 39.0, 32.1, 19.9; HRMS (ESI^+^) [C_31_H_34_O_8_Na]^+^ found 557.2124, [M + Na]^+^ calcld. 557.2146.

## Supplementary Materials

The following are available online at https://www.mdpi.com/2072-6651/12/12/740/s1, ^1^H and ^13^C NMR spectra for key compounds, plus additional ^1^H NMR NOE data for selected intermediate compounds.
