# Supplementary material for: Stereoselective Synthesis of the I–L Fragment of the Pacific Ciguatoxins
Source: Toxins (Basel). 2020 Nov 24;12(12):740. doi: 10.3390/toxins12120740 (PMC7760828; doi:10.3390/toxins12120740)
Supplement: Supplementary file 1 [file toxins-12-00740-s001.pdf]

Supplementary Materials: Stereoselective Synthesis of the I–L Fragment of the Pacific Ciguatoxins

Michael Popadynec and J. Stephen Clark

^{1}H and ^{13}C NMR Spectra for Key Compounds

	Page
^{1}H NMR spectrum of **7**	2
^{13}C NMR spectrum of **7**	3
^{1}H NMR spectrum of **8**	4
^{13}C NMR spectrum of **8**	5
^{1}H NMR spectrum of **10**	6
^{13}C NMR spectrum of **10**	7
^{1}H NMR spectrum of **10** with NOE enhancements (irradiation at CH 7′)	8
^{1}H NMR spectrum of **10** with NOE enhancements (irradiation at CH 9′)	9
^{1}H NMR spectrum of **11a**	10
^{13}C NMR spectrum of **11a**	11
^{1}H NMR spectrum of **11a** with NOE enhancements (irradiation at CH3)	12
^{1}H NMR spectrum of **11a** with NOE enhancements (irradiation at CH 7′)	13
^{1}H NMR spectrum of **11a** with NOE enhancements (irradiation at CH 9′)	14
^{1}H NMR spectrum of **11b**	15
^{13}C NMR spectrum of **11b**	16
^{1}H NMR spectrum of **11b** with NOE enhancements (irradiation at CH3)	17
^{1}H NMR spectrum of **11b** with NOE enhancements (irradiation at CH 7′)	18
^{1}H NMR spectrum of **11b** with NOE enhancements (irradiation at CH 9′)	19
^{1}H NMR spectrum of **13**	20
^{13}C NMR spectrum of **13**	21
^{1}H NMR spectrum of **13** with NOE enhancements (irradiation at CH 3′)	22
^{1}H NMR spectrum of **13** with NOE enhancements (irradiation at CH3)	23
^{1}H NMR spectrum of **13** with NOE enhancements (irradiation at CH 5′)	24
^{1}H NMR spectrum of **13** with NOE enhancements (irradiation at CH 7′)	25
^{1}H NMR spectrum of **14**	26
^{13}C NMR spectrum of **14**	27
^{1}H NMR spectrum of **15**	28
^{13}C NMR spectrum of **15**	29
^{1}H NMR spectrum of **16**	30
^{13}C NMR spectrum of **16**	31
^{1}H NMR spectrum of **17**	32
^{13}C NMR spectrum of **17**	33
^{1}H NMR spectrum of **18**	34
^{13}C NMR spectrum of **18**	35
^{1}H NMR spectrum of **19**	36
^{13}C NMR spectrum of **19**	37
^{1}H NMR spectrum of **20**	38
^{13}C NMR spectrum of **20**	39
^{1}H NMR spectrum of **25**	40
^{13}C NMR spectrum of **25**	41

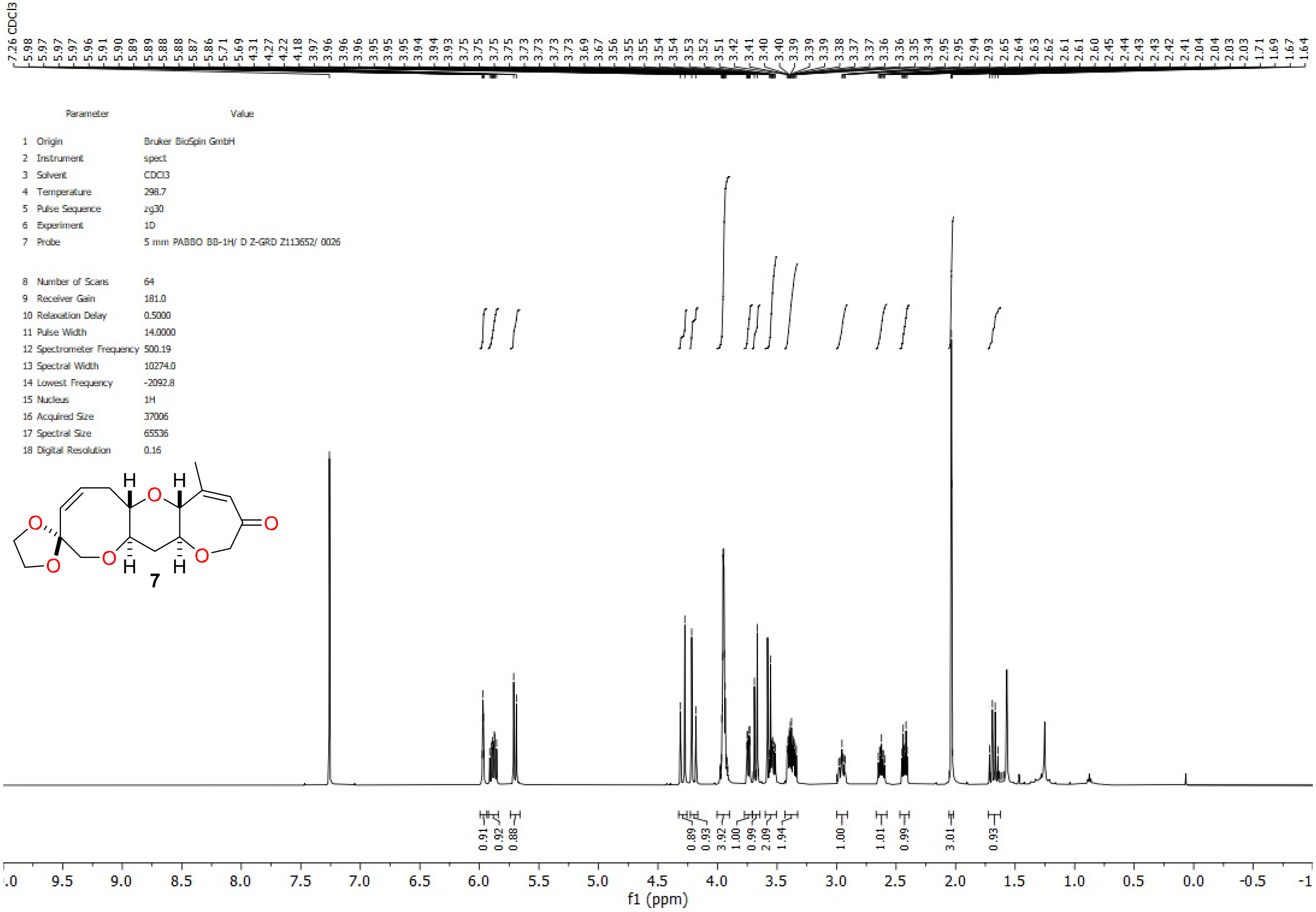
7.26 CDCl3
Parameter Value
1 Origin Bruker BioSpin GmbH
2 Instrument spect
3 Solvent CDCl3
4 Temperature 298.7
5 Pulse Sequence zg30
6 Experiment 1D
7 Probe 5 mm PABBO BB-1H/ D Z-GRD Z113652/ 0026
8 Number of Scans 64
9 Receiver Gain 181.0
10 Relaxation Delay 0.5000
11 Pulse Width 14.0000
12 Spectrometer Frequency 500.19
13 Spectral Width 10274.0
14 Lowest Frequency -2092.8
15 Nucleus 1H
16 Acquired Size 37006
17 Spectral Size 65536
18 Digital Resolution 0.16
7
0.91 0.92 0.88 0.89 0.93 3.92 1.00 0.99 2.09 1.94 1.00 1.01 0.99 3.01 0.93
f1 (ppm)

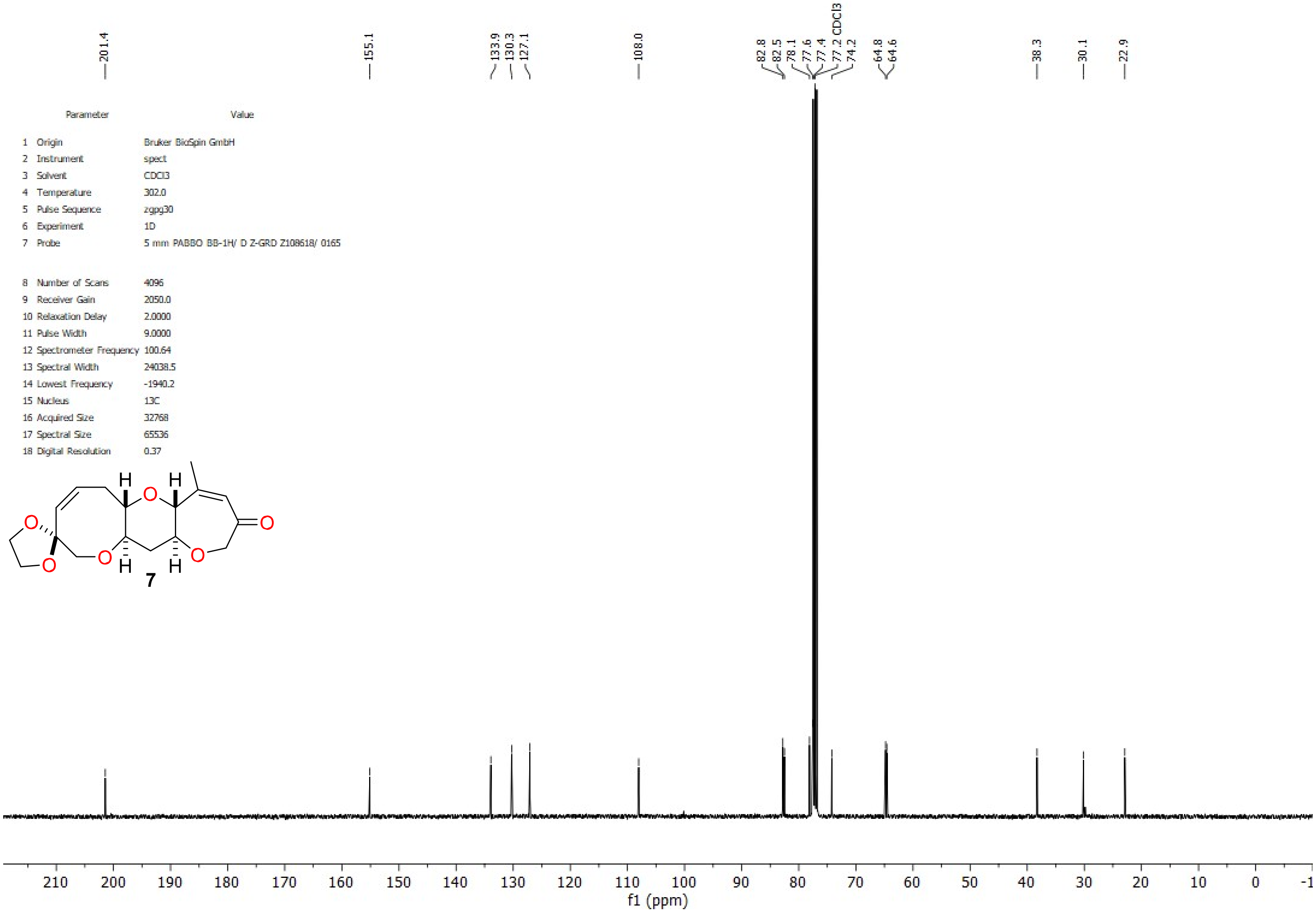
201.4
155.1
133.9
130.3
127.1
108.0
82.8
82.5
78.1
77.6
77.4
77.2 CDCl3
74.2
64.8
64.6
38.3
30.1
22.9
Parameter Value
1 Origin Bruker BioSpin GmbH
2 Instrument spect
3 Solvent CDCl3
4 Temperature 302.0
5 Pulse Sequence zgpg30
6 Experiment 1D
7 Probe 5 mm PABBO BB-1H/ D Z-GRD Z108618/ 0165
8 Number of Scans 4096
9 Receiver Gain 2050.0
10 Relaxation Delay 2.0000
11 Pulse Width 9.0000
12 Spectrometer Frequency 100.64
13 Spectral Width 24038.5
14 Lowest Frequency -1940.2
15 Nucleus 13C
16 Acquired Size 32768
17 Spectral Size 65536
18 Digital Resolution 0.37
7
210 200 190 180 170 160 150 140 130 120 110 100 90 80 70 60 50 40 30 20 10 0 -1
f1 (ppm)

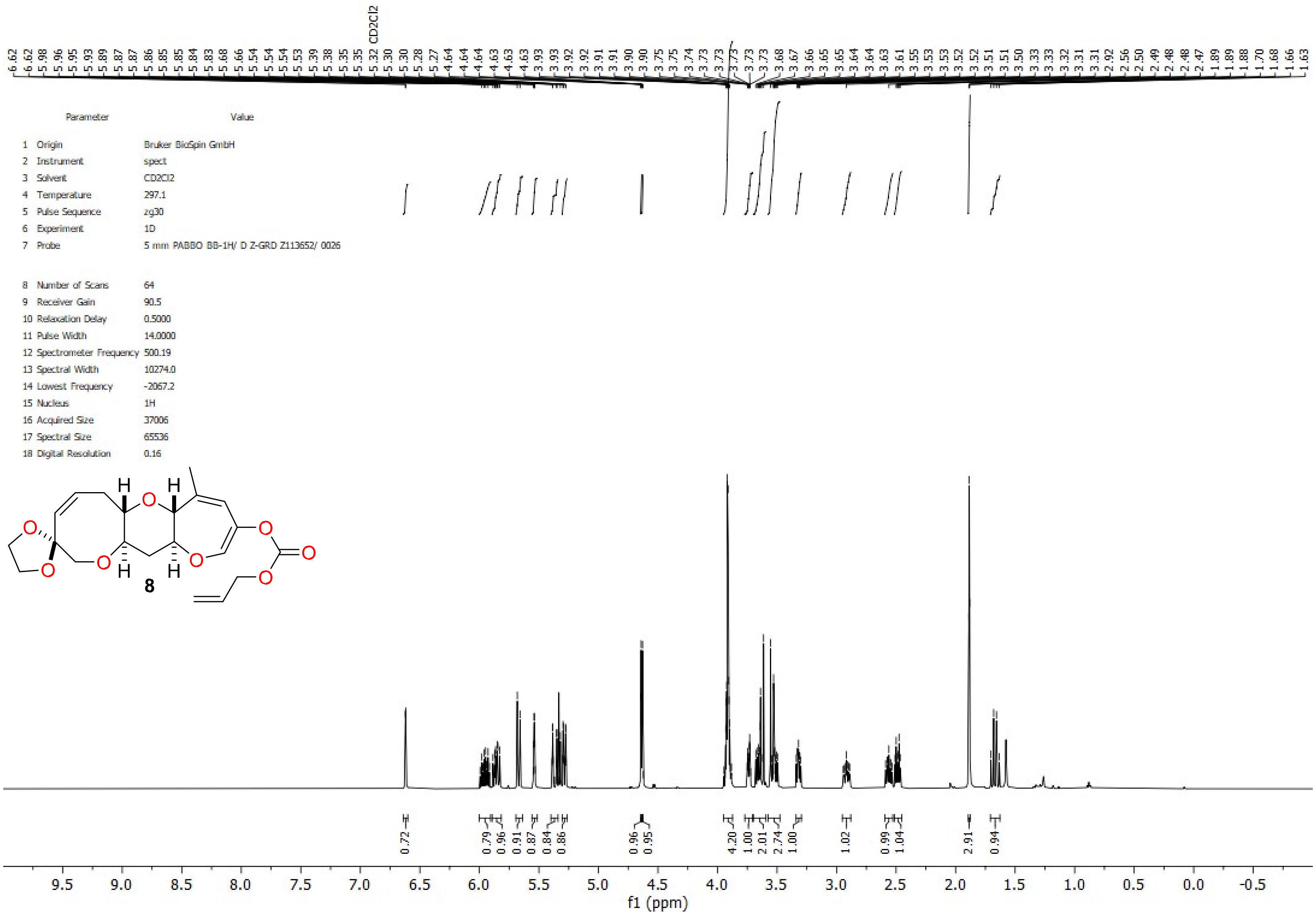
CD2Cl2
6.62 6.62 5.98 5.96 5.95 5.93 5.89 5.87 5.87 5.86 5.85 5.85 5.84 5.83 5.68 5.66 5.54 5.54 5.54 5.53 5.39 5.38 5.35 5.35 5.32 5.30 5.30 5.28 5.27 4.64 4.64 4.64 4.63 4.63 4.63 3.93 3.93 3.92 3.92 3.91 3.91 3.90 3.90 3.75 3.75 3.74 3.73 3.73 3.73 3.73 3.73 3.68 3.67 3.66 3.65 3.65 3.64 3.64 3.63 3.61 3.55 3.53 3.53 3.52 3.52 3.51 3.51 3.50 3.33 3.33 3.32 3.31 3.31 2.92 2.56 2.50 2.49 2.48 2.48 2.47 1.89 1.89 1.88 1.70 1.68 1.66 1.63
Parameter Value
1 Origin Bruker BioSpin GmbH
2 Instrument spect
3 Solvent CD2Cl2
4 Temperature 297.1
5 Pulse Sequence zg30
6 Experiment 1D
7 Probe 5 mm PABBO BB-1H/ D Z-GRD Z113652/ 0026
8 Number of Scans 64
9 Receiver Gain 90.5
10 Relaxation Delay 0.5000
11 Pulse Width 14.0000
12 Spectrometer Frequency 500.19
13 Spectral Width 10274.0
14 Lowest Frequency -2067.2
15 Nucleus 1H
16 Acquired Size 37006
17 Spectral Size 65536
18 Digital Resolution 0.16
8
0.72 0.79 0.96 0.91 0.87 0.84 0.86 0.96 0.95 4.20 1.00 2.01 2.74 1.00 1.02 0.99 1.04 2.91 0.94
9.5 9.0 8.5 8.0 7.5 7.0 6.5 6.0 5.5 5.0 4.5 4.0 3.5 3.0 2.5 2.0 1.5 1.0 0.5 0.0 -0.5
f1 (ppm)

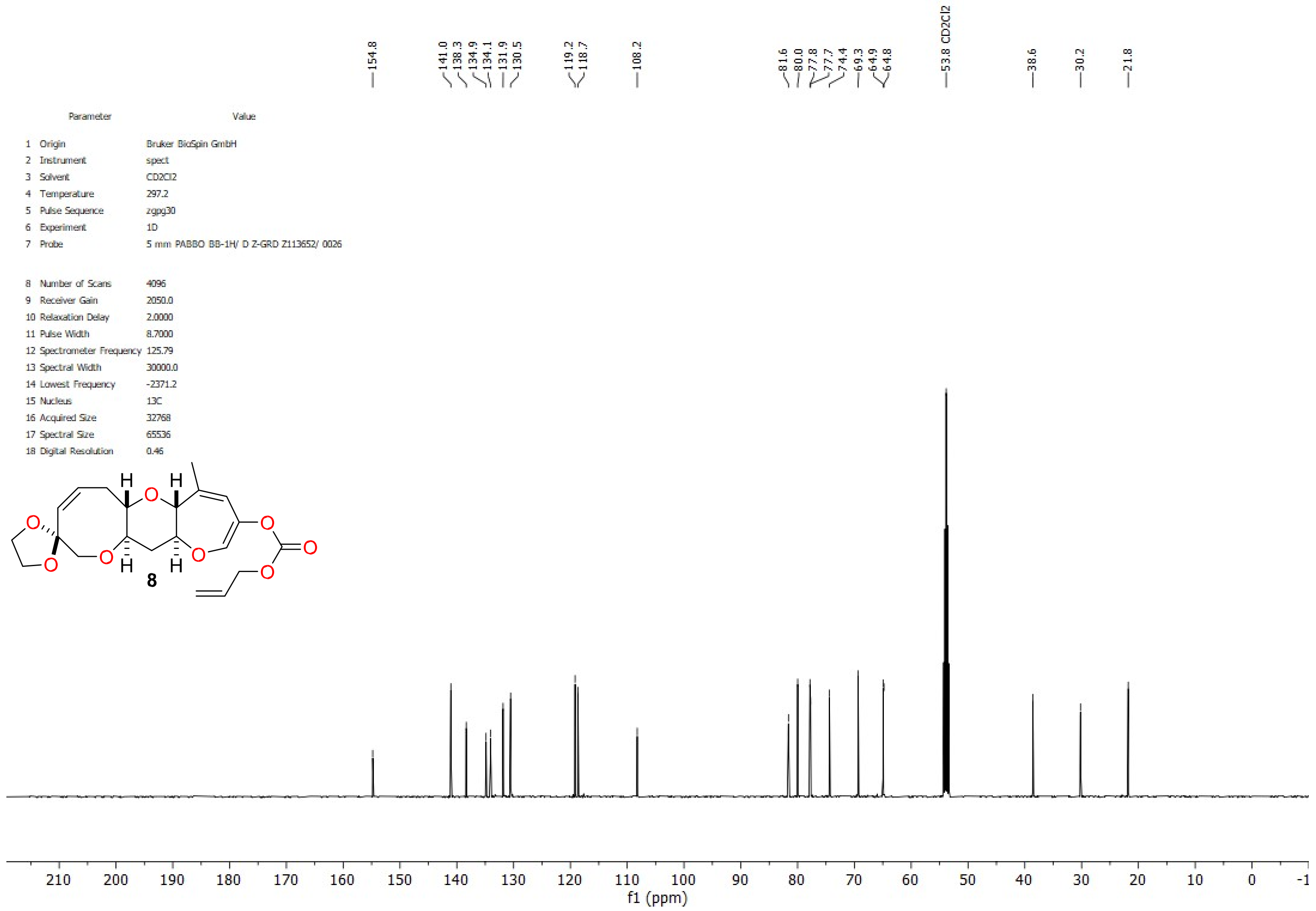

	Parameter	Value
1	Origin	Bruker BioSpin GmbH
2	Instrument	spect
3	Solvent	CD2Cl2
4	Temperature	297.2
5	Pulse Sequence	zgpg30
6	Experiment	1D
7	Probe	5 mm PABBO BB-1H/ D Z-GRD Z113652/ 0026
8	Number of Scans	4096
9	Receiver Gain	2050.0
10	Relaxation Delay	2.0000
11	Pulse Width	8.7000
12	Spectrometer Frequency	125.79
13	Spectral Width	30000.0
14	Lowest Frequency	-2371.2
15	Nucleus	13C
16	Acquired Size	32768
17	Spectral Size	65536
18	Digital Resolution	0.46

8

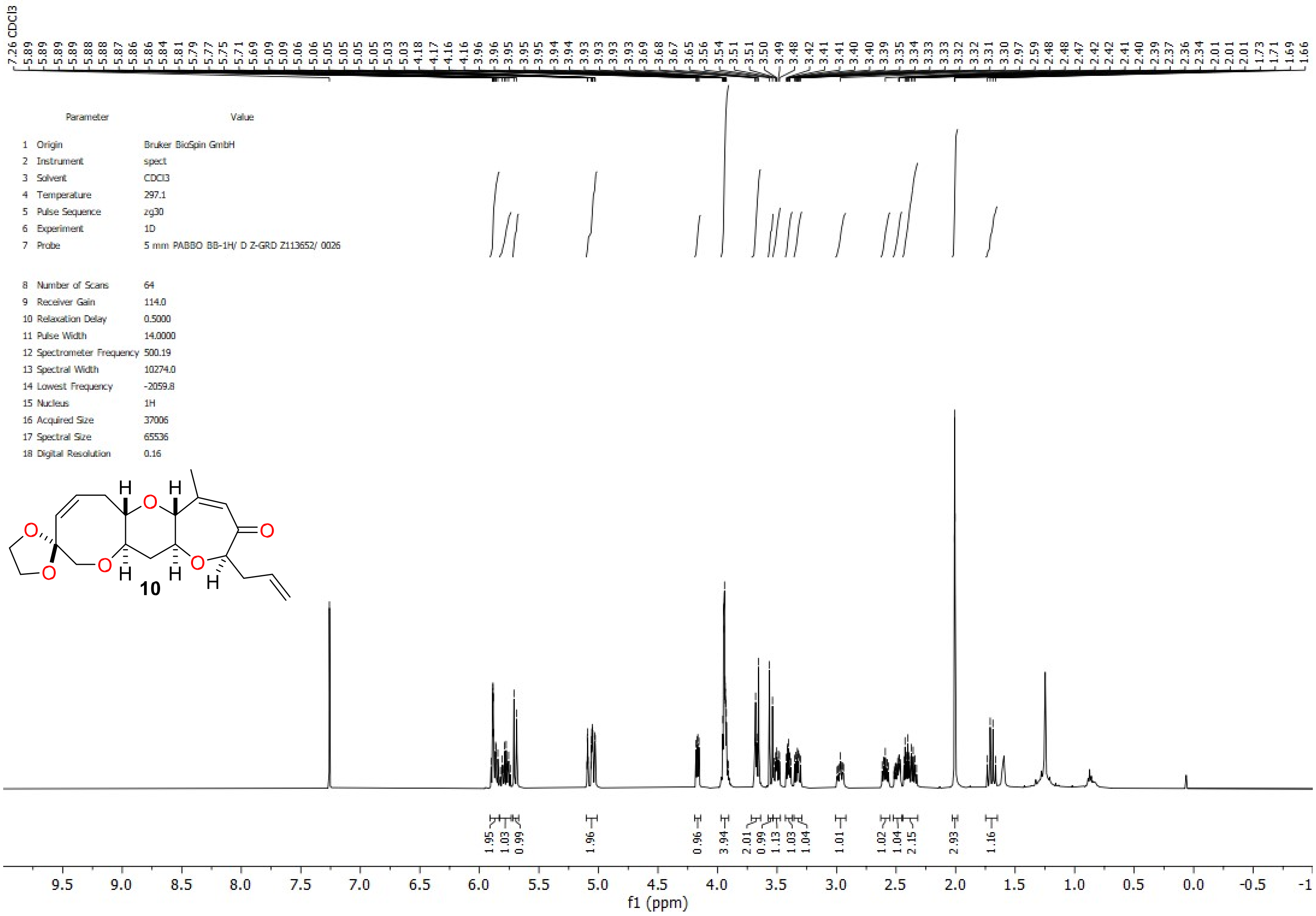
Parameter Value
1 Origin Bruker BioSpin GmbH
2 Instrument spect
3 Solvent CDCl3
4 Temperature 297.1
5 Pulse Sequence zg30
6 Experiment 1D
7 Probe 5 mm PABBO BB-1H/ D Z-GRD Z113652/ 0026
8 Number of Scans 64
9 Receiver Gain 114.0
10 Relaxation Delay 0.5000
11 Pulse Width 14.0000
12 Spectrometer Frequency 500.19
13 Spectral Width 10274.0
14 Lowest Frequency -2059.8
15 Nucleus 1H
16 Acquired Size 37006
17 Spectral Size 65536
18 Digital Resolution 0.16
10
f1 (ppm)

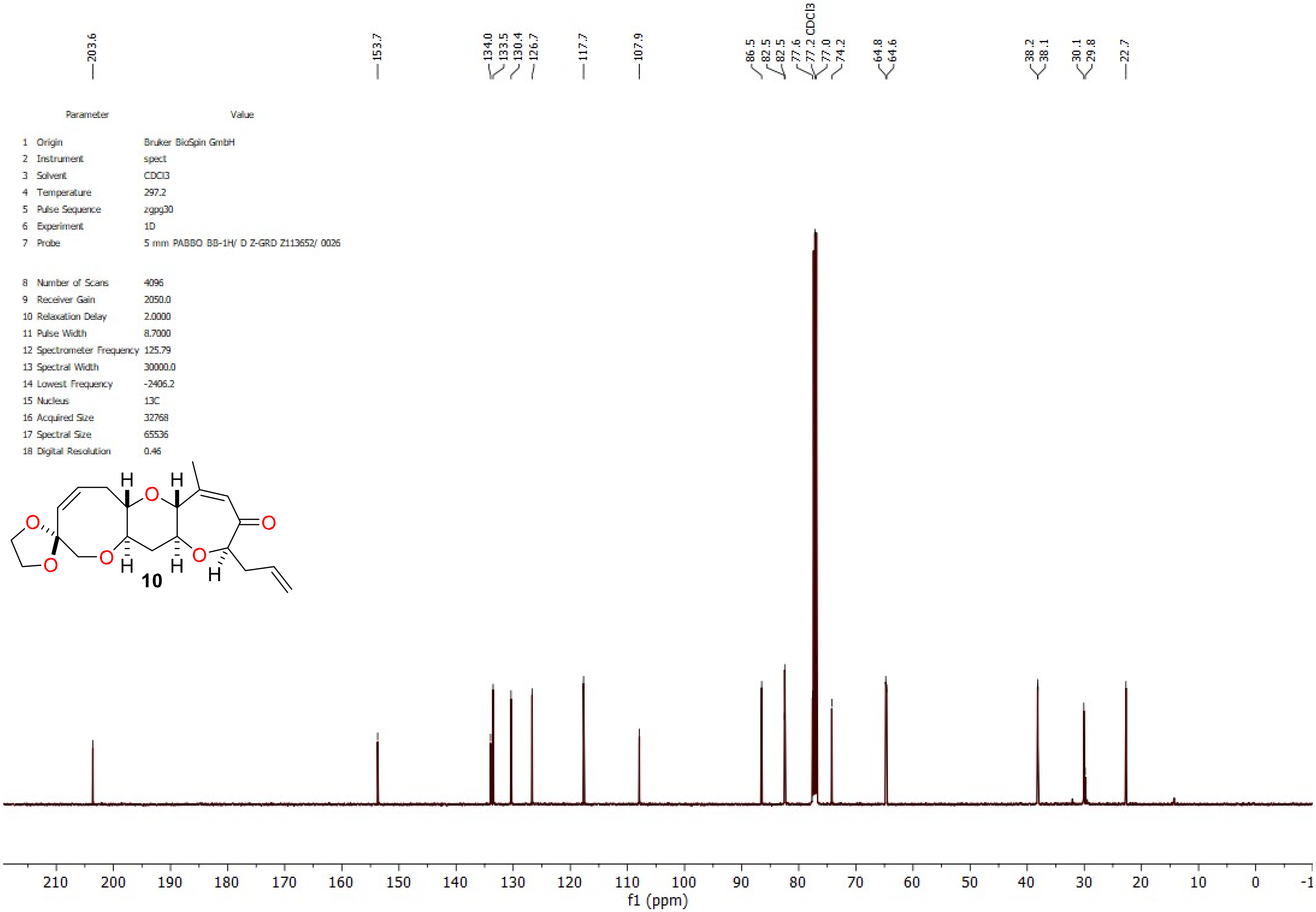
203.6
153.7
134.0
133.5
130.4
126.7
117.7
107.9
86.5
82.5
82.5
77.6
77.2 CDCl3
77.0
74.2
64.8
64.6
38.2
38.1
30.1
29.8
22.7
Parameter Value
1 Origin Bruker BioSpin GmbH
2 Instrument spect
3 Solvent CDCl3
4 Temperature 297.2
5 Pulse Sequence zgpg30
6 Experiment 1D
7 Probe 5 mm PABBO BB-1H/ D Z-GRD Z113652/ 0026
8 Number of Scans 4096
9 Receiver Gain 2050.0
10 Relaxation Delay 2.0000
11 Pulse Width 8.7000
12 Spectrometer Frequency 125.79
13 Spectral Width 30000.0
14 Lowest Frequency -2406.2
15 Nucleus 13C
16 Acquired Size 32768
17 Spectral Size 65536
18 Digital Resolution 0.46
10
210 200 190 180 170 160 150 140 130 120 110 100 90 80 70 60 50 40 30 20 10 0 -1
f1 (ppm)

Parameter	Value
1 Origin	Bruker BioSpin GmbH
2 Instrument	spect
3 Solvent	CDCl3
4 Temperature	297.2
5 Pulse Sequence	selnogp
6 Experiment	1D
7 Probe	5 mm PABBO BB-1H/ D Z-GRD Z113652/ 0026
8 Number of Scans	128
9 Receiver Gain	406.0
10 Relaxation Delay	2.0000
11 Pulse Width	14.0000
12 Spectrometer Frequency	500.19
13 Spectral Width	10000.0
14 Lowest Frequency	-2923.7
15 Nucleus	1H
16 Acquired Size	16384
17 Spectral Size	65536
18 Digital Resolution	0.15

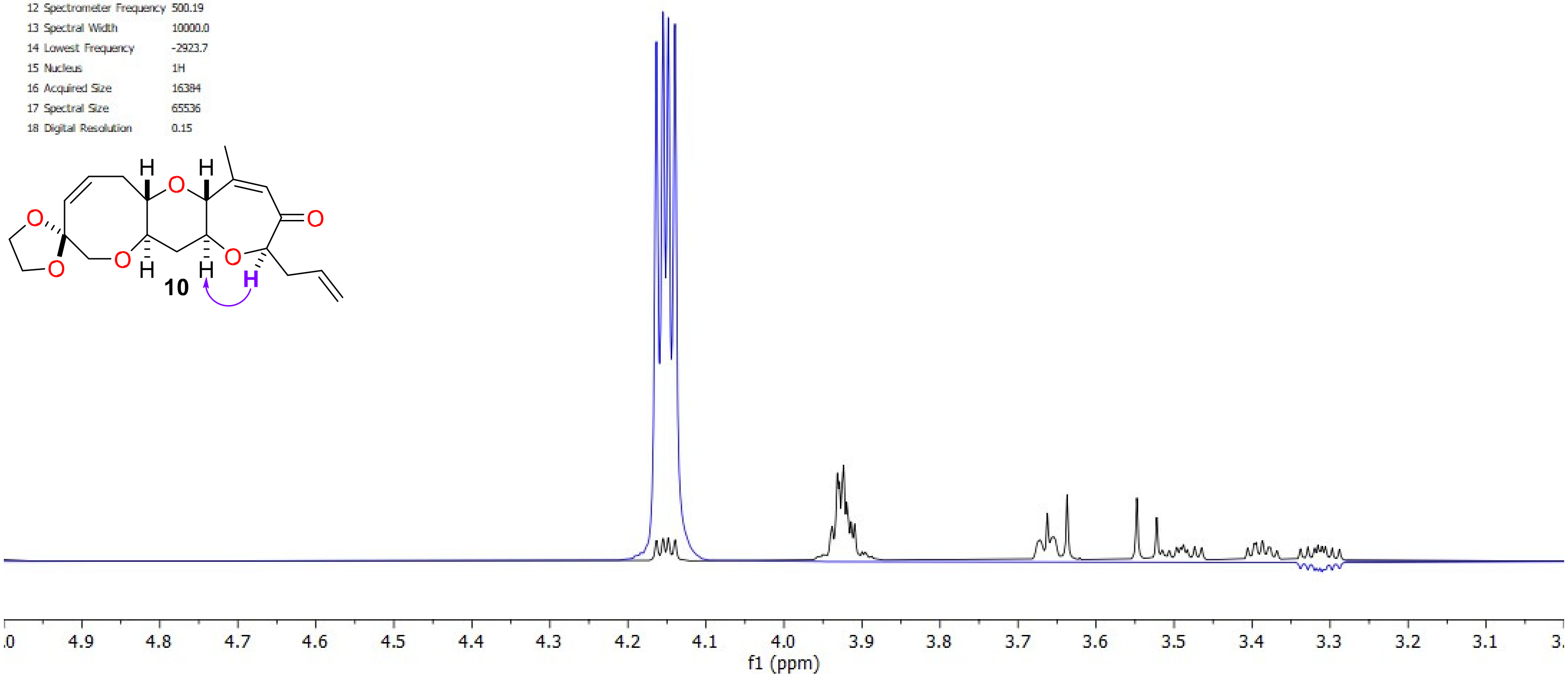

	Parameter	Value
1	Origin	Bruker BioSpin GmbH
2	Instrument	spect
3	Solvent	CDCl3
4	Temperature	297.2
5	Pulse Sequence	selnogp
6	Experiment	1D
7	Probe	5 mm PABBO BB-1H/ D Z-GRD Z113652/ 0026
8	Number of Scans	128
9	Receiver Gain	406.0
10	Relaxation Delay	2.0000
11	Pulse Width	14.0000
12	Spectrometer Frequency	500.19
13	Spectral Width	10000.0
14	Lowest Frequency	-2923.7
15	Nucleus	1H
16	Acquired Size	16384
17	Spectral Size	65536
18	Digital Resolution	0.15

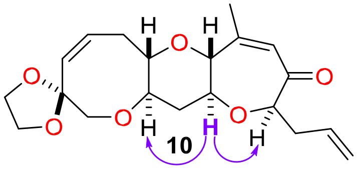

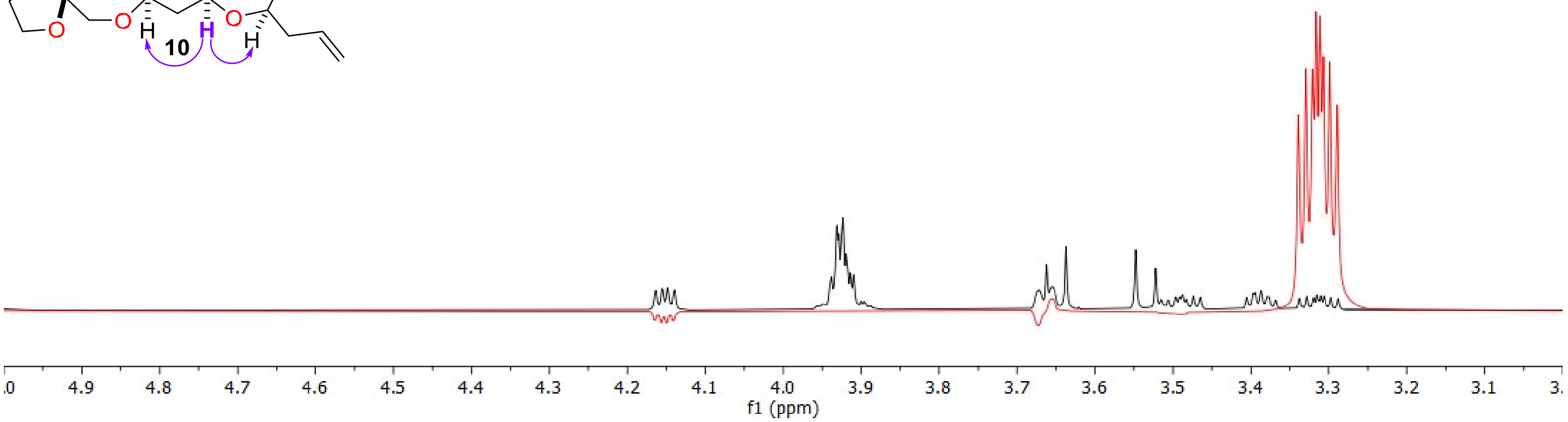

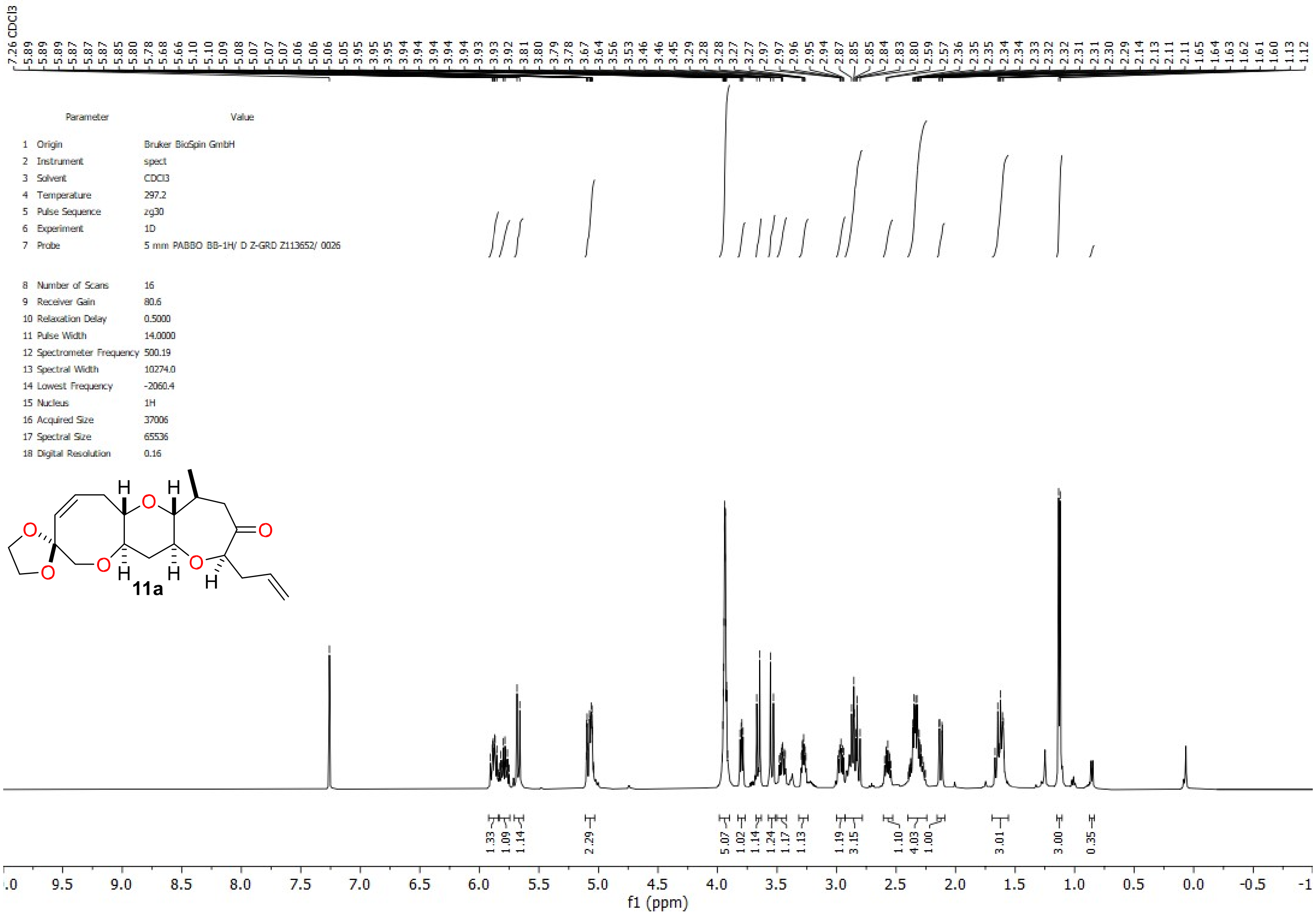
Parameter Value
1 Origin Bruker BioSpin GmbH
2 Instrument spect
3 Solvent CDCl3
4 Temperature 297.2
5 Pulse Sequence zg30
6 Experiment 1D
7 Probe 5 mm PABBO BB-1H/ D Z-GRD Z113652/ 0026
8 Number of Scans 16
9 Receiver Gain 80.6
10 Relaxation Delay 0.5000
11 Pulse Width 14.0000
12 Spectrometer Frequency 500.19
13 Spectral Width 10274.0
14 Lowest Frequency -2060.4
15 Nucleus 1H
16 Acquired Size 37006
17 Spectral Size 65536
18 Digital Resolution 0.16
11a
f1 (ppm)

	Parameter	Value
1	Origin	Bruker BioSpin GmbH
2	Instrument	spect
3	Solvent	CDCl3
4	Temperature	297.2
5	Pulse Sequence	zgpg30
6	Experiment	1D
7	Probe	5 mm PABBO BB-1H/ D Z-GRD Z113652/ 0026
8	Number of Scans	6144
9	Receiver Gain	2050.0
10	Relaxation Delay	2.0000
11	Pulse Width	8.7000
12	Spectrometer Frequency	125.79
13	Spectral Width	30000.0
14	Lowest Frequency	-2408.0
15	Nucleus	13C
16	Acquired Size	32768
17	Spectral Size	65536
18	Digital Resolution	0.46

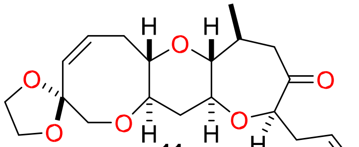

11a

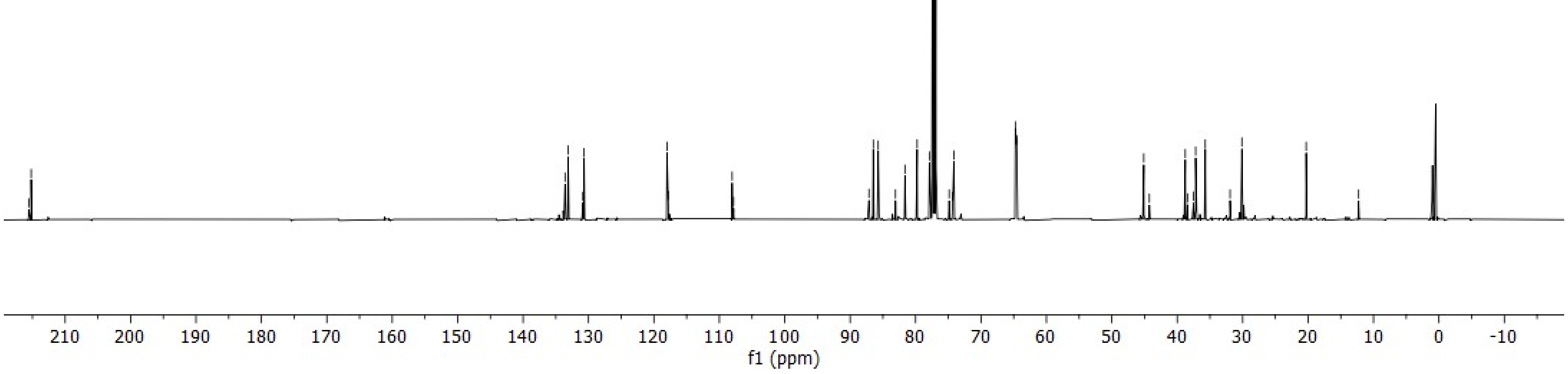

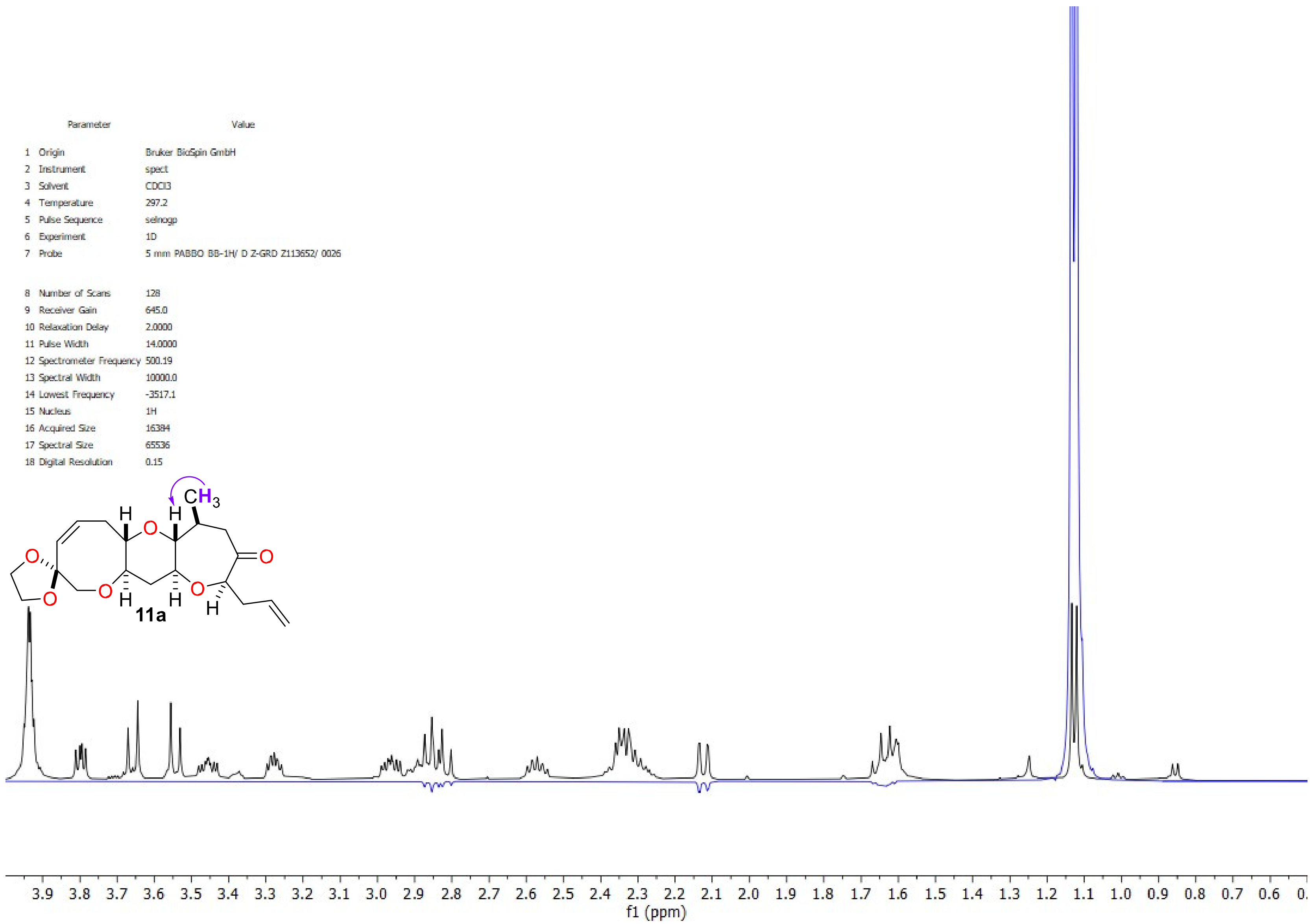
Parameter
Value
1 Origin Bruker BioSpin GmbH
2 Instrument spect
3 Solvent CDCl3
4 Temperature 297.2
5 Pulse Sequence selnogp
6 Experiment 1D
7 Probe 5 mm PABBO BB-1H/ D Z-GRD Z113652/ 0026
8 Number of Scans 128
9 Receiver Gain 645.0
10 Relaxation Delay 2.0000
11 Pulse Width 14.0000
12 Spectrometer Frequency 500.19
13 Spectral Width 10000.0
14 Lowest Frequency -3517.1
15 Nucleus 1H
16 Acquired Size 16384
17 Spectral Size 65536
18 Digital Resolution 0.15
CH3
11a
3.9 3.8 3.7 3.6 3.5 3.4 3.3 3.2 3.1 3.0 2.9 2.8 2.7 2.6 2.5 2.4 2.3 2.2 2.1 2.0 1.9 1.8 1.7 1.6 1.5 1.4 1.3 1.2 1.1 1.0 0.9 0.8 0.7 0.6 0.
f1 (ppm)

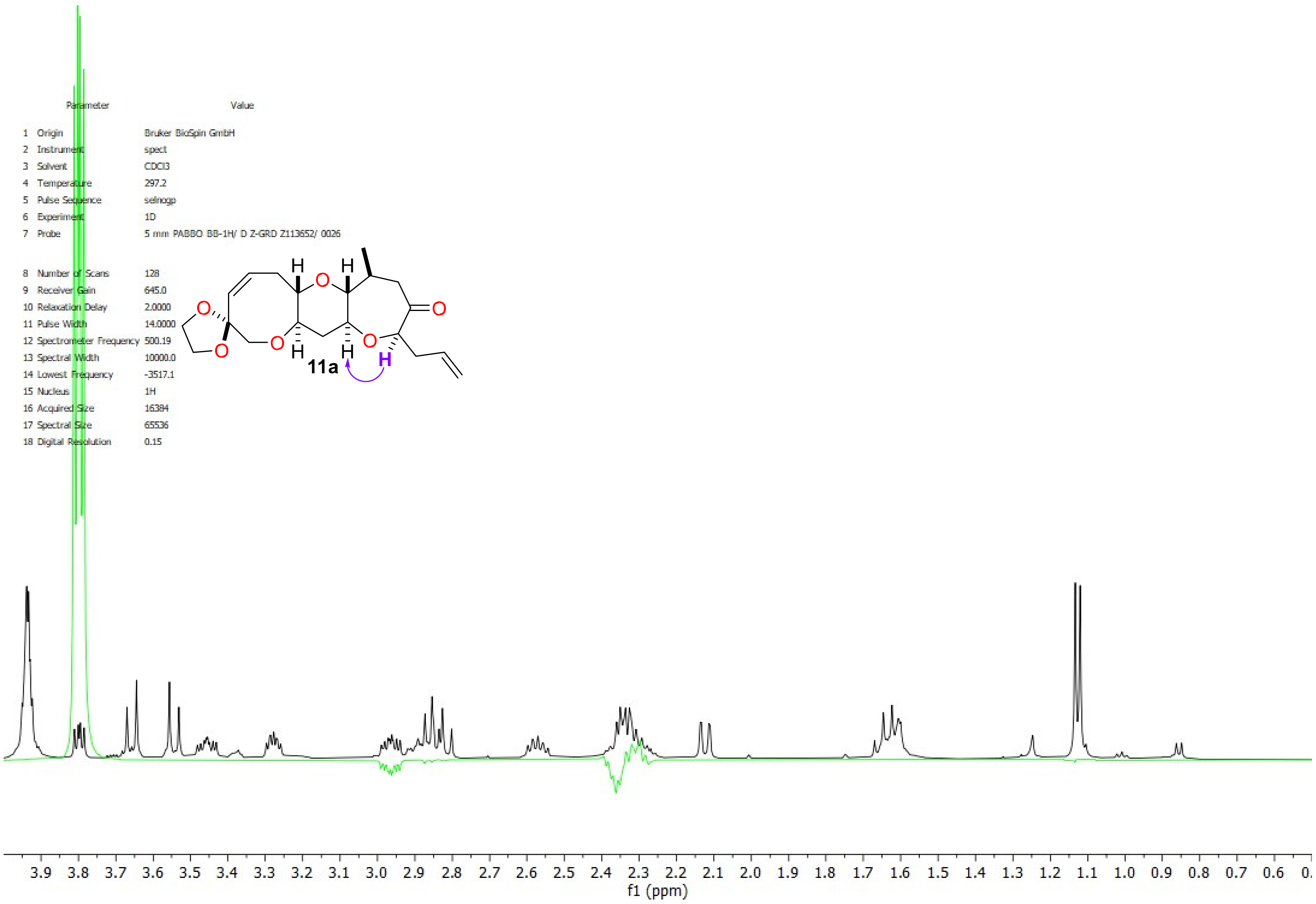
Parameter Value
1 Origin Bruker BioSpin GmbH
2 Instrument spect
3 Solvent CDCl3
4 Temperature 297.2
5 Pulse Sequence selnogp
6 Experiment 1D
7 Probe 5 mm PABBO BB-1H/ D Z-GRD Z113652/ 0026
8 Number of Scans 128
9 Receiver Gain 645.0
10 Relaxation Delay 2.0000
11 Pulse Width 14.0000
12 Spectrometer Frequency 500.19
13 Spectral Width 10000.0
14 Lowest Frequency -3517.1
15 Nucleus 1H
16 Acquired Size 16384
17 Spectral Size 65536
18 Digital Resolution 0.15
11a
f1 (ppm)

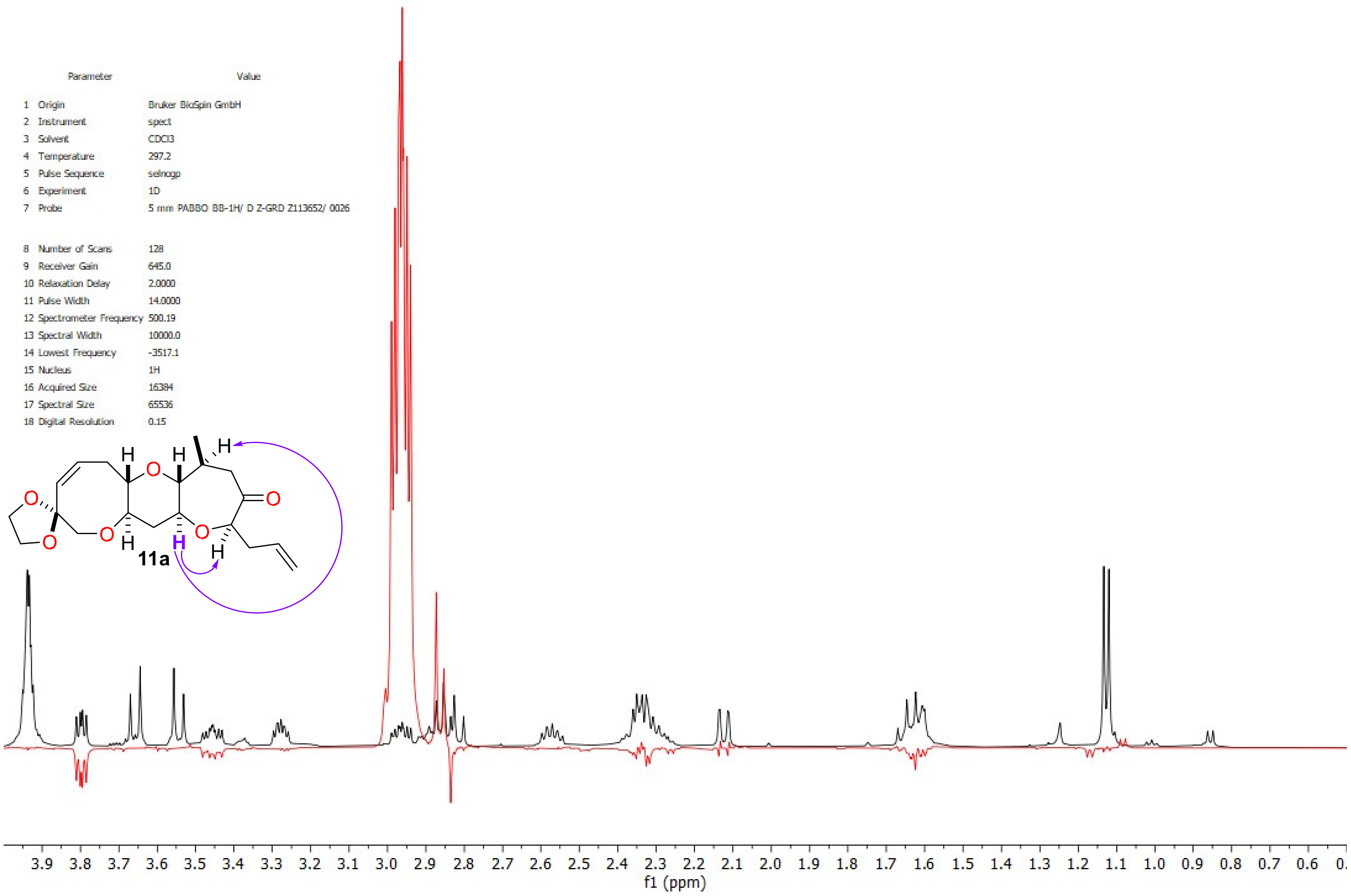

Parameter Value
1 Origin Bruker BioSpin GmbH
2 Instrument spect
3 Solvent CDCl3
4 Temperature 297.2
5 Pulse Sequence selnogp
6 Experiment 1D
7 Probe 5 mm PABBO BB-1H/ D Z-GRD Z113652/ 0026
8 Number of Scans 128
9 Receiver Gain 645.0
10 Relaxation Delay 2.0000
11 Pulse Width 14.0000
12 Spectrometer Frequency 500.19
13 Spectral Width 10000.0
14 Lowest Frequency -3517.1
15 Nucleus 1H
16 Acquired Size 16384
17 Spectral Size 65536
18 Digital Resolution 0.15
11a
3.9 3.8 3.7 3.6 3.5 3.4 3.3 3.2 3.1 3.0 2.9 2.8 2.7 2.6 2.5 2.4 2.3 2.2 2.1 2.0 1.9 1.8 1.7 1.6 1.5 1.4 1.3 1.2 1.1 1.0 0.9 0.8 0.7 0.6
f1 (ppm)

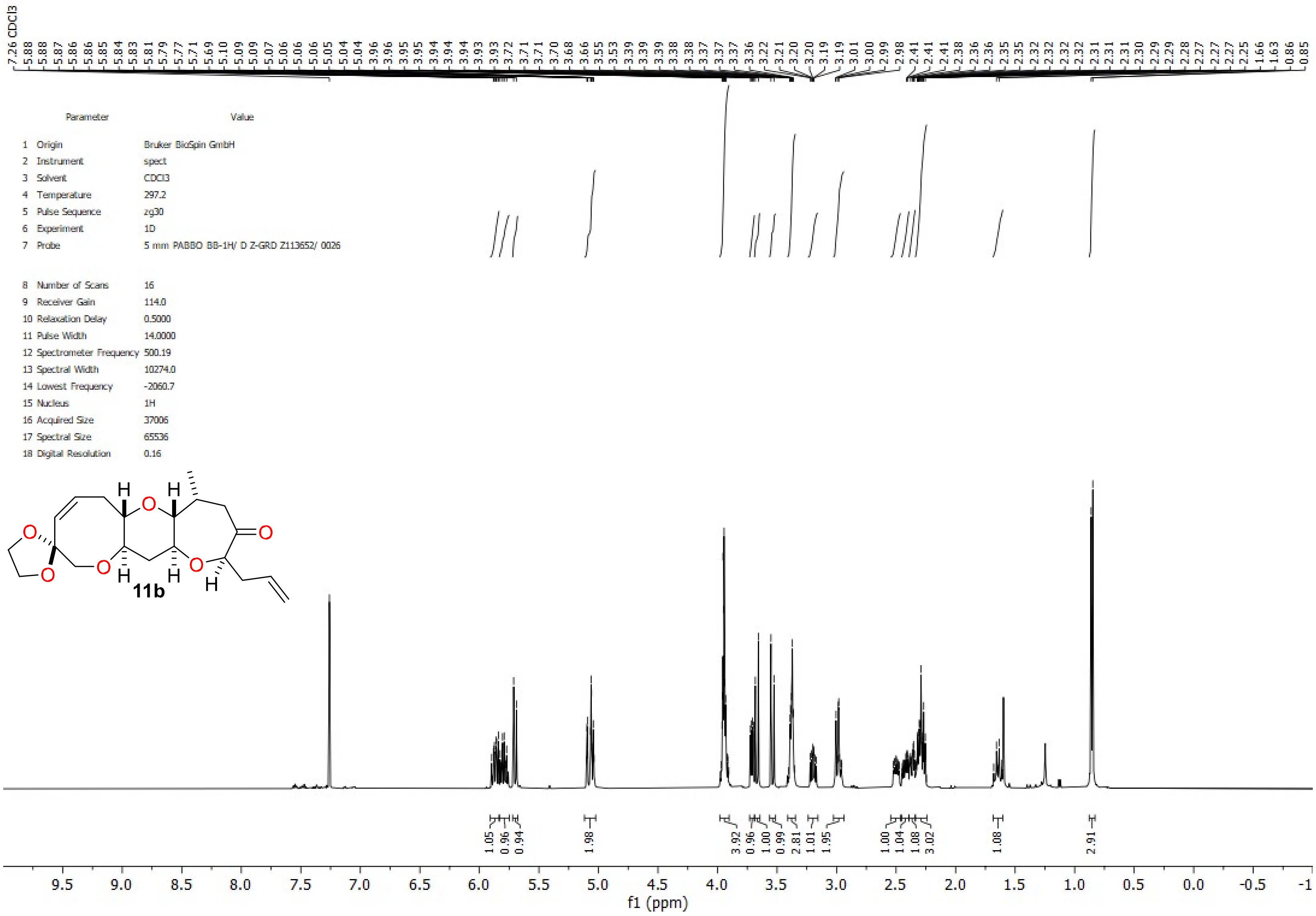
7.26 CDCl3
5.88 5.88 5.87 5.86 5.86 5.85 5.84 5.83 5.81 5.79 5.77 5.71 5.69 5.10 5.09 5.09 5.07 5.06 5.06 5.05 5.04 5.04 3.96 3.96 3.95 3.95 3.94 3.94 3.93 3.93 3.72 3.71 3.71 3.70 3.68 3.66 3.55 3.53 3.39 3.39 3.39 3.38 3.38 3.37 3.37 3.37 3.36 3.22 3.21 3.20 3.20 3.19 3.19 3.01 3.00 2.99 2.98 2.41 2.41 2.41 2.38 2.36 2.36 2.35 2.35 2.32 2.32 2.32 2.32 2.31 2.31 2.31 2.30 2.29 2.29 2.28 2.27 2.27 2.27 2.25 1.66 1.63 0.86 0.85
Parameter Value
1 Origin Bruker BioSpin GmbH
2 Instrument spect
3 Solvent CDCl3
4 Temperature 297.2
5 Pulse Sequence zg30
6 Experiment 1D
7 Probe 5 mm PABBO BB-1H/ D Z-GRD Z113652/ 0026
8 Number of Scans 16
9 Receiver Gain 114.0
10 Relaxation Delay 0.5000
11 Pulse Width 14.0000
12 Spectrometer Frequency 500.19
13 Spectral Width 10274.0
14 Lowest Frequency -2060.7
15 Nucleus 1H
16 Acquired Size 37006
17 Spectral Size 65536
18 Digital Resolution 0.16
11b
1.05 0.96 0.94 1.98 3.92 0.96 1.00 0.99 2.81 1.01 1.95 1.00 1.04 1.08 3.02 1.08 2.91
9.5 9.0 8.5 8.0 7.5 7.0 6.5 6.0 5.5 5.0 4.5 4.0 3.5 3.0 2.5 2.0 1.5 1.0 0.5 0.0 -0.5 -1
f1 (ppm)

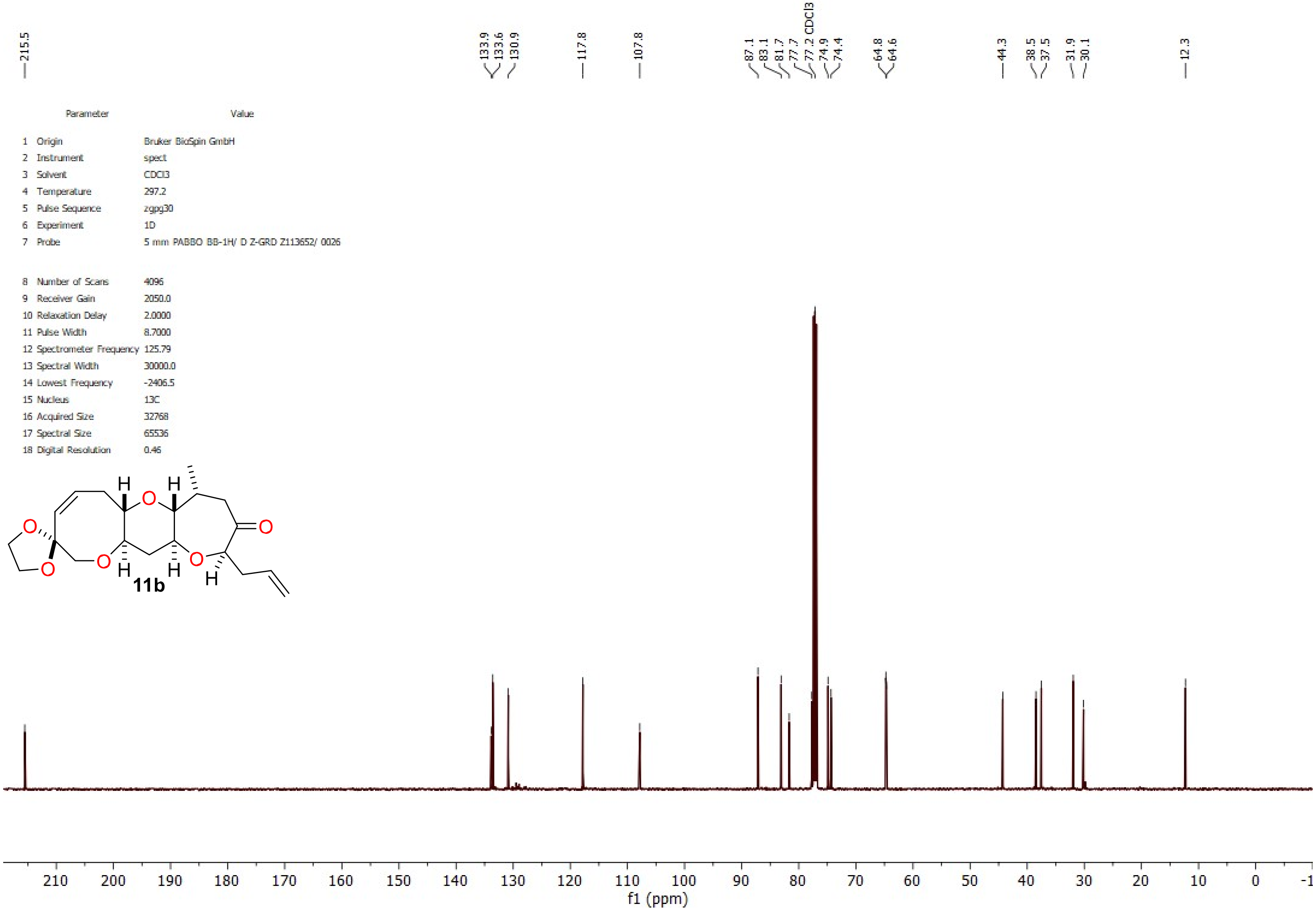

	Parameter	Value
1	Origin	Bruker BioSpin GmbH
2	Instrument	spect
3	Solvent	CDCl3
4	Temperature	297.2
5	Pulse Sequence	zgpg30
6	Experiment	1D
7	Probe	5 mm PABBO BB-1H/ D Z-GRD Z113652/ 0026
8	Number of Scans	4096
9	Receiver Gain	2050.0
10	Relaxation Delay	2.0000
11	Pulse Width	8.7000
12	Spectrometer Frequency	125.79
13	Spectral Width	30000.0
14	Lowest Frequency	-2406.5
15	Nucleus	13C
16	Acquired Size	32768
17	Spectral Size	65536
18	Digital Resolution	0.46

11b

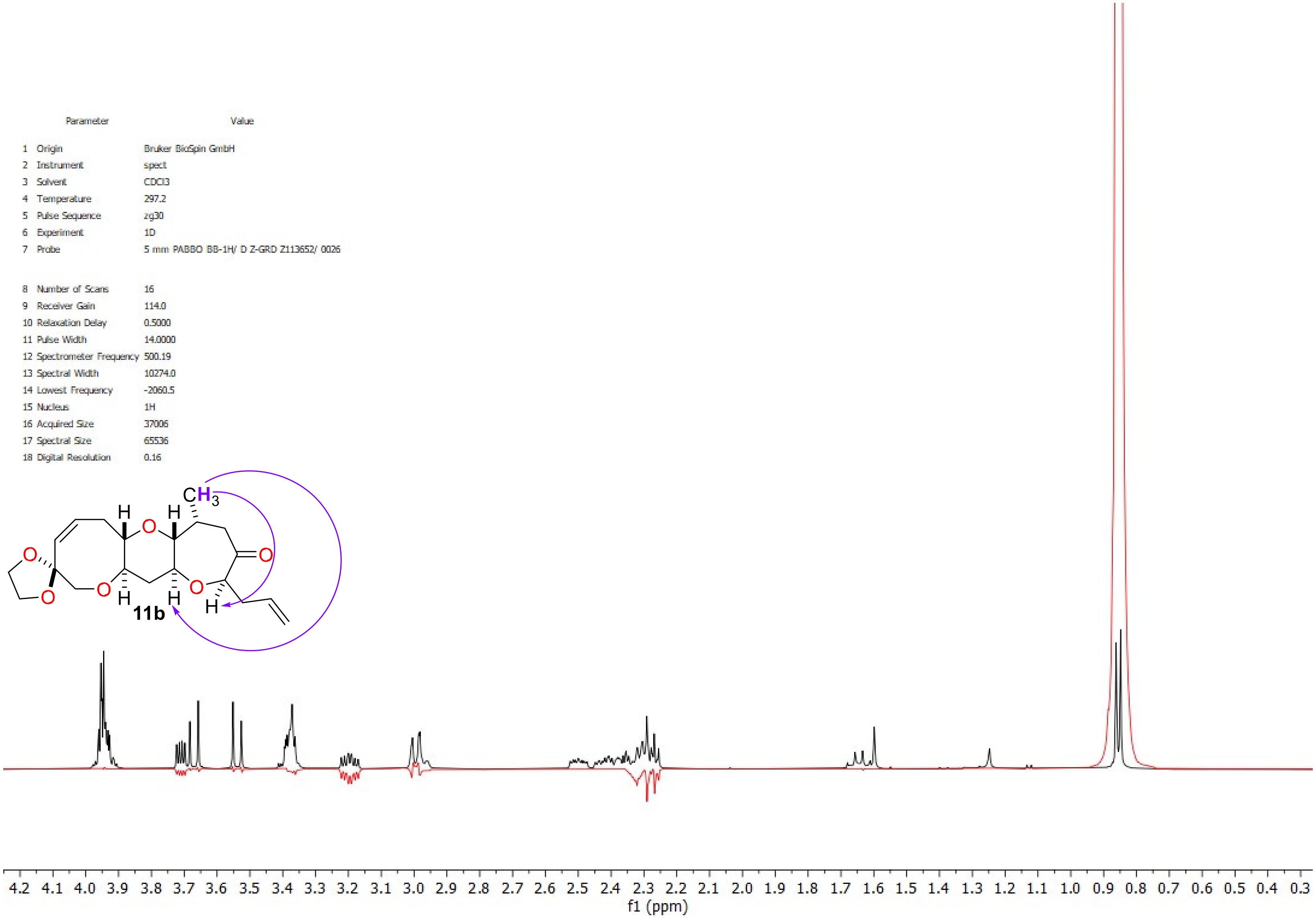
Parameter Value
1 Origin Bruker BioSpin GmbH
2 Instrument spect
3 Solvent CDCl3
4 Temperature 297.2
5 Pulse Sequence zg30
6 Experiment 1D
7 Probe 5 mm PABBO BB-1H/ D Z-GRD Z113652/ 0026
8 Number of Scans 16
9 Receiver Gain 114.0
10 Relaxation Delay 0.5000
11 Pulse Width 14.0000
12 Spectrometer Frequency 500.19
13 Spectral Width 10274.0
14 Lowest Frequency -2060.5
15 Nucleus 1H
16 Acquired Size 37006
17 Spectral Size 65536
18 Digital Resolution 0.16
11b
4.2 4.1 4.0 3.9 3.8 3.7 3.6 3.5 3.4 3.3 3.2 3.1 3.0 2.9 2.8 2.7 2.6 2.5 2.4 2.3 2.2 2.1 2.0 1.9 1.8 1.7 1.6 1.5 1.4 1.3 1.2 1.1 1.0 0.9 0.8 0.7 0.6 0.5 0.4 0.3
f1 (ppm)

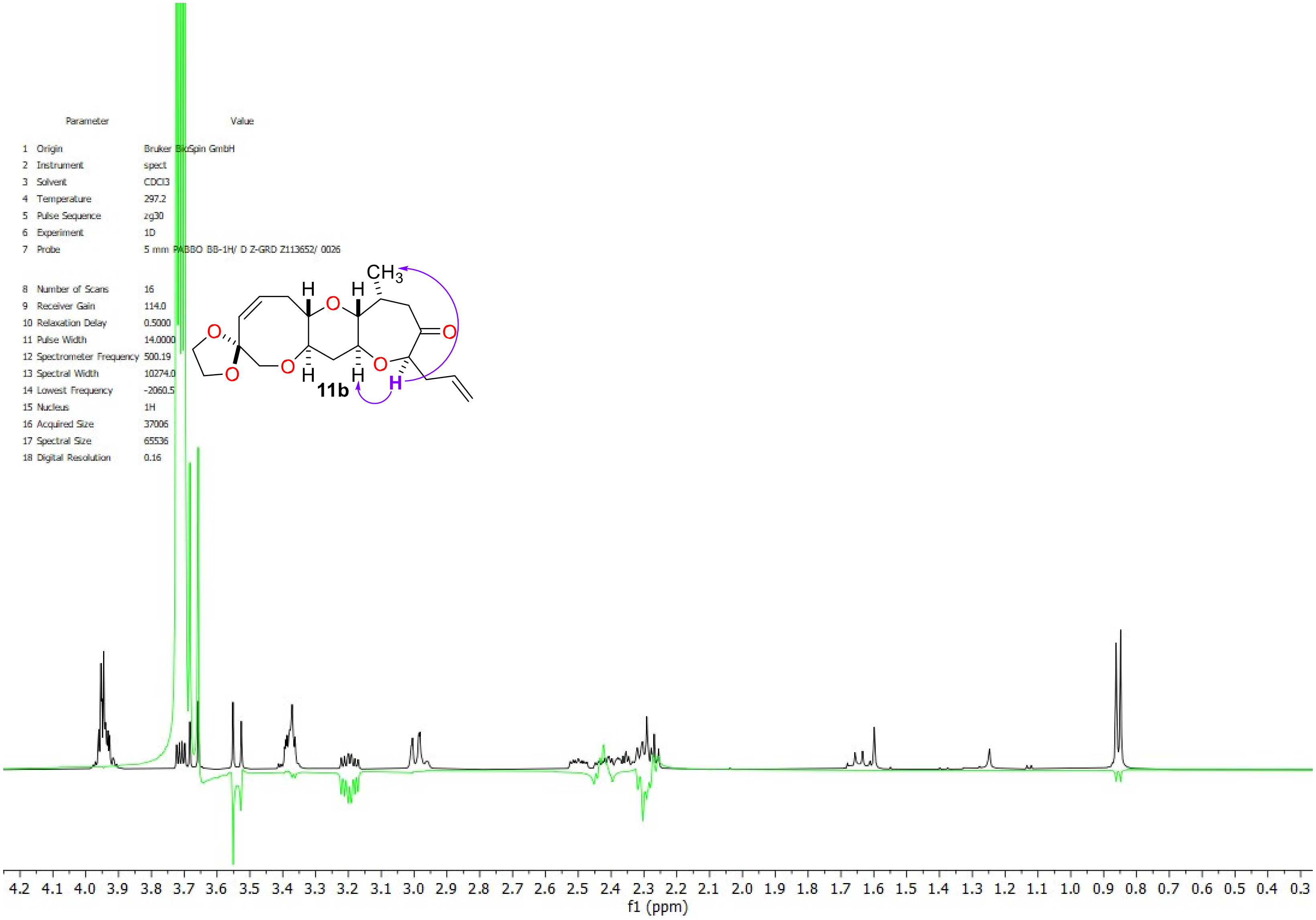

Parameter
Value
1 Origin
Bruker BioSpin GmbH
2 Instrument
spect
3 Solvent
CDCl3
4 Temperature
297.2
5 Pulse Sequence
zg30
6 Experiment
1D
7 Probe
5 mm PABBO BB-1H/ D Z-GRD Z113652/ 0026
8 Number of Scans
16
9 Receiver Gain
114.0
10 Relaxation Delay
0.5000
11 Pulse Width
14.0000
12 Spectrometer Frequency
500.19
13 Spectral Width
10274.0
14 Lowest Frequency
-2060.5
15 Nucleus
1H
16 Acquired Size
37006
17 Spectral Size
65536
18 Digital Resolution
0.16
CH3
H
O
11b
4.2 4.1 4.0 3.9 3.8 3.7 3.6 3.5 3.4 3.3 3.2 3.1 3.0 2.9 2.8 2.7 2.6 2.5 2.4 2.3 2.2 2.1 2.0 1.9 1.8 1.7 1.6 1.5 1.4 1.3 1.2 1.1 1.0 0.9 0.8 0.7 0.6 0.5 0.4 0.3
f1 (ppm)

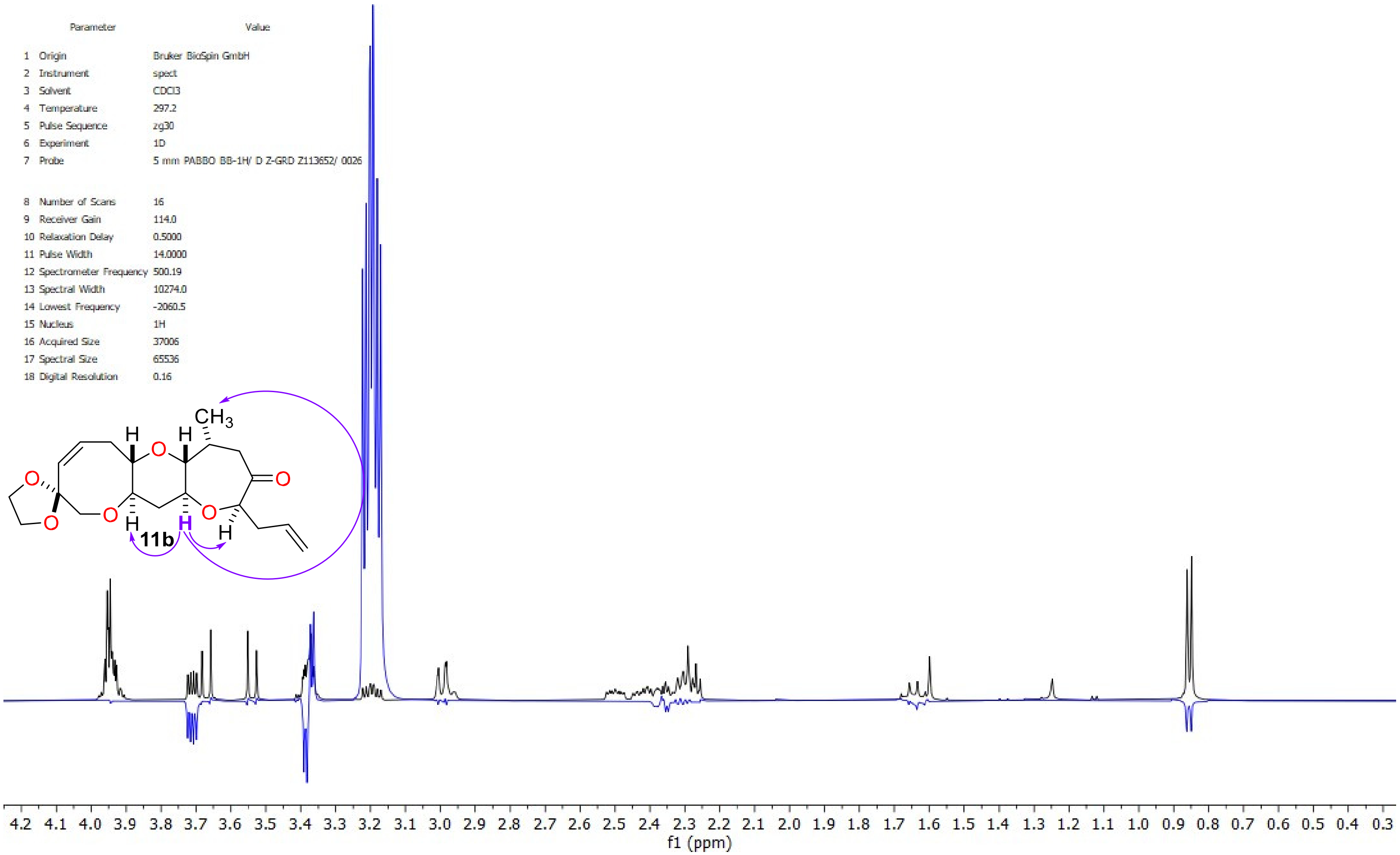
Parameter Value
1 Origin Bruker BioSpin GmbH
2 Instrument spect
3 Solvent CDCl3
4 Temperature 297.2
5 Pulse Sequence zg30
6 Experiment 1D
7 Probe 5 mm PABBO BB-1H/ D Z-GRD Z113652/ 0026
8 Number of Scans 16
9 Receiver Gain 114.0
10 Relaxation Delay 0.5000
11 Pulse Width 14.0000
12 Spectrometer Frequency 500.19
13 Spectral Width 10274.0
14 Lowest Frequency -2060.5
15 Nucleus 1H
16 Acquired Size 37006
17 Spectral Size 65536
18 Digital Resolution 0.16
11b
4.2 4.1 4.0 3.9 3.8 3.7 3.6 3.5 3.4 3.3 3.2 3.1 3.0 2.9 2.8 2.7 2.6 2.5 2.4 2.3 2.2 2.1 2.0 1.9 1.8 1.7 1.6 1.5 1.4 1.3 1.2 1.1 1.0 0.9 0.8 0.7 0.6 0.5 0.4 0.3
f1 (ppm)

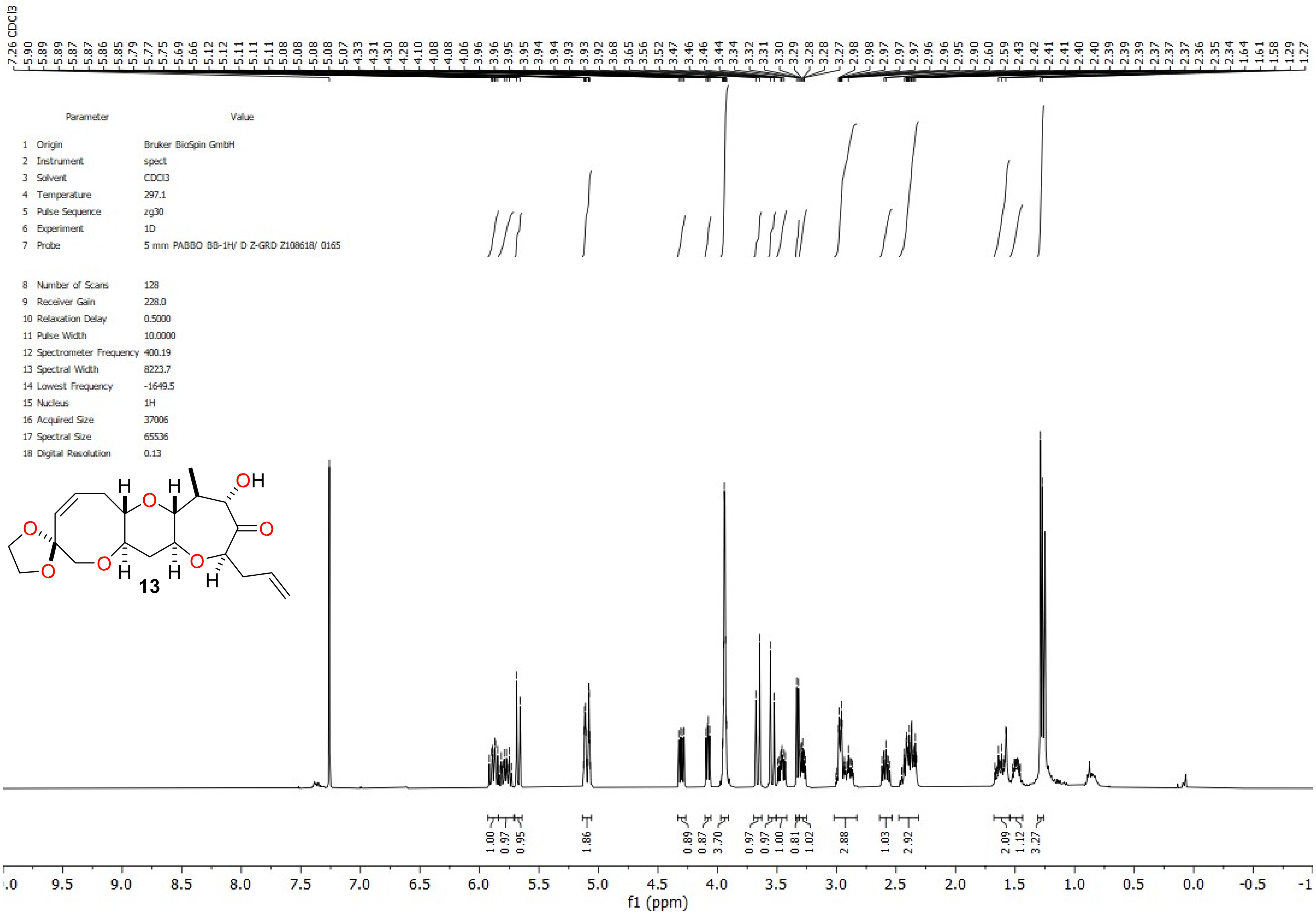
Parameter Value
1 Origin Bruker BioSpin GmbH
2 Instrument spect
3 Solvent CDCl3
4 Temperature 297.1
5 Pulse Sequence zg30
6 Experiment 1D
7 Probe 5 mm PABBO BB-1H/ D Z-GRD Z108618/ 0165
8 Number of Scans 128
9 Receiver Gain 228.0
10 Relaxation Delay 0.5000
11 Pulse Width 10.0000
12 Spectrometer Frequency 400.19
13 Spectral Width 8223.7
14 Lowest Frequency -1649.5
15 Nucleus 1H
16 Acquired Size 37006
17 Spectral Size 65536
18 Digital Resolution 0.13
13
f1 (ppm)

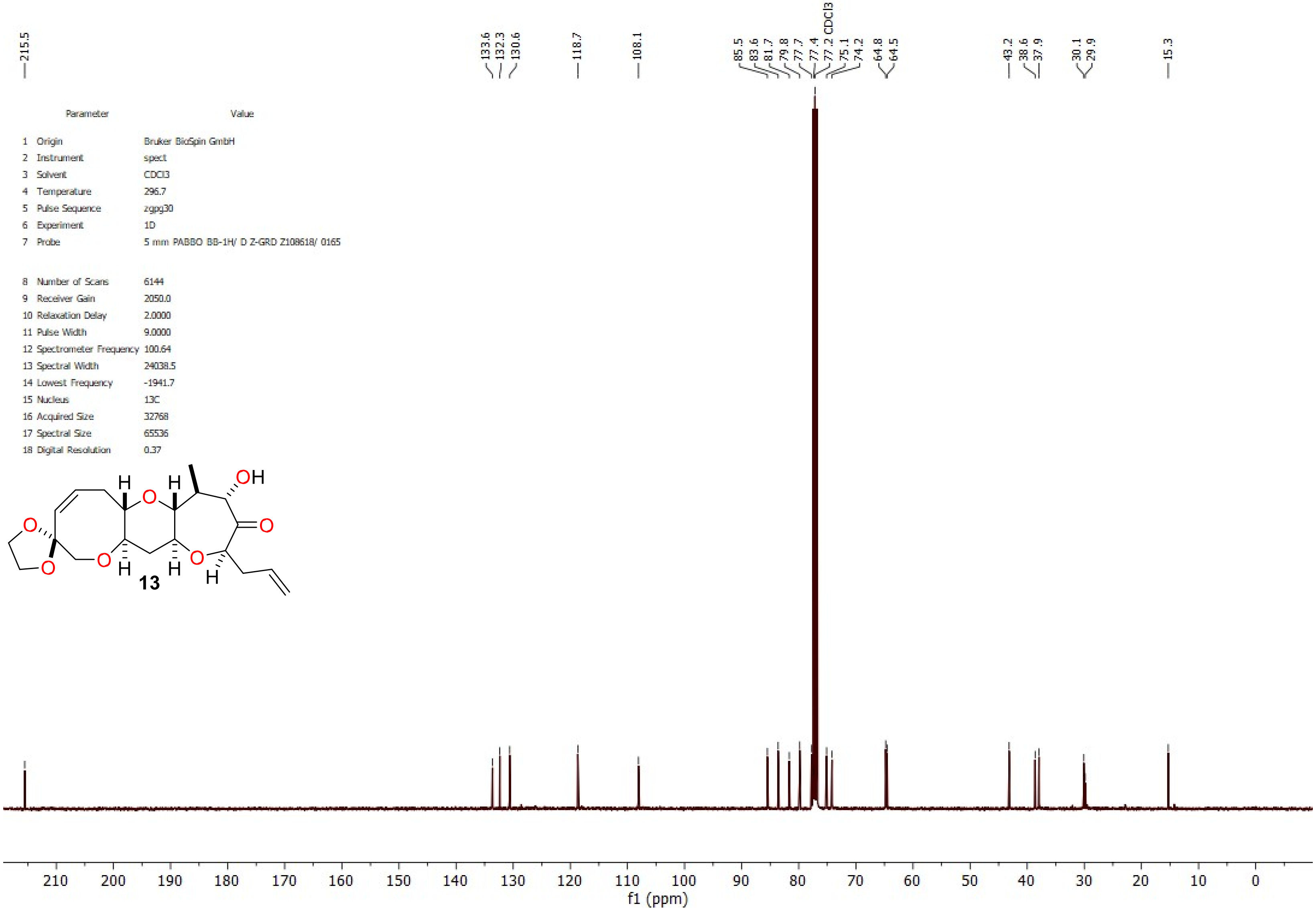
215.5
133.6
132.3
130.6
118.7
108.1
85.5
83.6
81.7
79.8
77.7
77.4
77.2 CDCl3
75.1
74.2
64.8
64.5
43.2
38.6
37.9
30.1
29.9
15.3
Parameter Value
1 Origin Bruker BioSpin GmbH
2 Instrument spect
3 Solvent CDCl3
4 Temperature 296.7
5 Pulse Sequence zgpg30
6 Experiment 1D
7 Probe 5 mm PABBO BB-1H/ D Z-GRD Z108618/ 0165
8 Number of Scans 6144
9 Receiver Gain 2050.0
10 Relaxation Delay 2.0000
11 Pulse Width 9.0000
12 Spectrometer Frequency 100.64
13 Spectral Width 24038.5
14 Lowest Frequency -1941.7
15 Nucleus 13C
16 Acquired Size 32768
17 Spectral Size 65536
18 Digital Resolution 0.37
13
210 200 190 180 170 160 150 140 130 120 110 100 90 80 70 60 50 40 30 20 10 0
f1 (ppm)

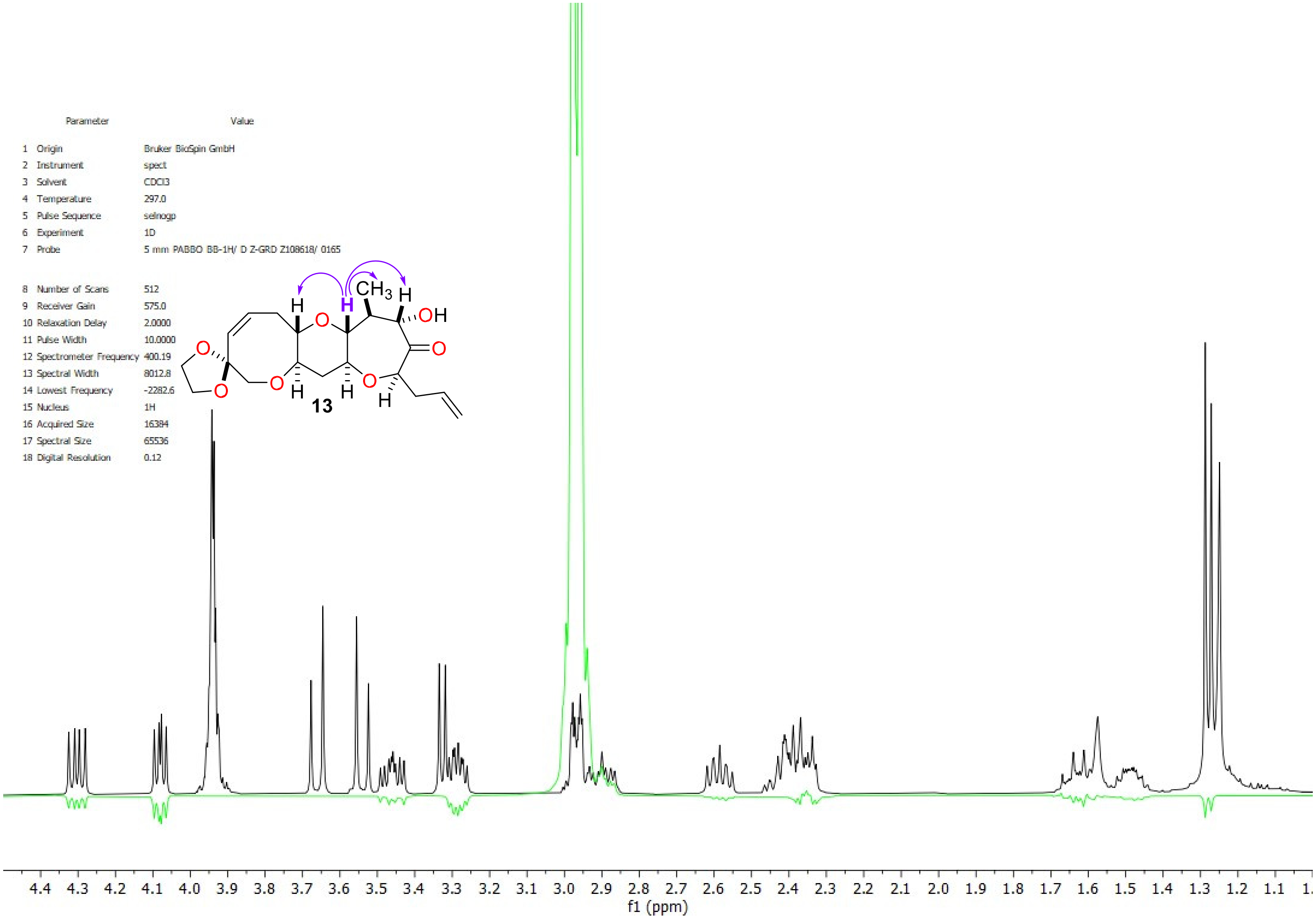
Parameter Value
1 Origin Bruker BioSpin GmbH
2 Instrument spect
3 Solvent CDCl3
4 Temperature 297.0
5 Pulse Sequence selnogp
6 Experiment 1D
7 Probe 5 mm PABBO BB-1H/ D Z-GRD Z108618/ 0165
8 Number of Scans 512
9 Receiver Gain 575.0
10 Relaxation Delay 2.0000
11 Pulse Width 10.0000
12 Spectrometer Frequency 400.19
13 Spectral Width 8012.8
14 Lowest Frequency -2282.6
15 Nucleus 1H
16 Acquired Size 16384
17 Spectral Size 65536
18 Digital Resolution 0.12
13
f1 (ppm)

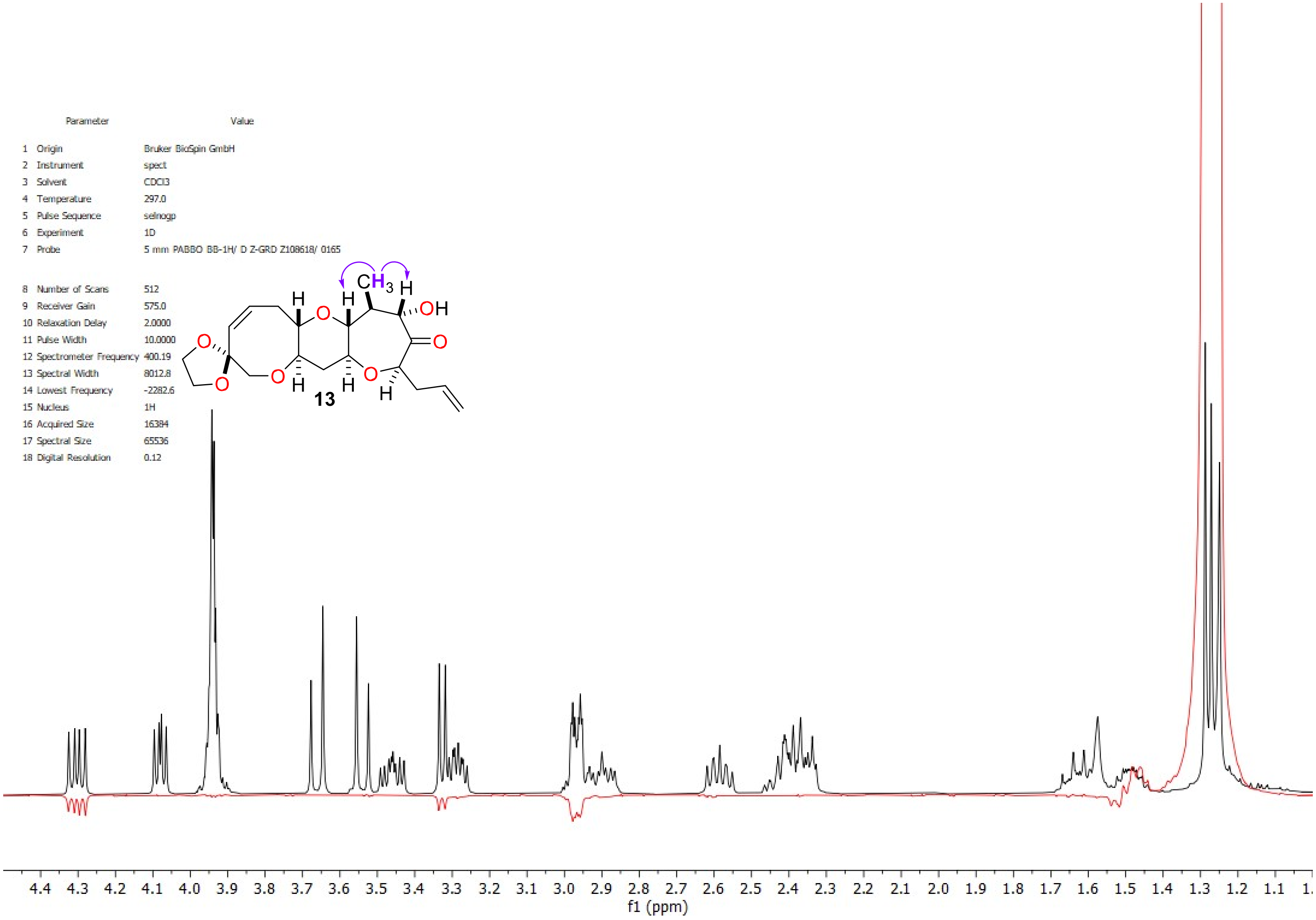

Parameter Value
1 Origin Bruker BioSpin GmbH
2 Instrument spect
3 Solvent CDCl3
4 Temperature 297.0
5 Pulse Sequence selnogp
6 Experiment 1D
7 Probe 5 mm PABBO BB-1H/ D Z-GRD Z108618/ 0165
8 Number of Scans 512
9 Receiver Gain 575.0
10 Relaxation Delay 2.0000
11 Pulse Width 10.0000
12 Spectrometer Frequency 400.19
13 Spectral Width 8012.8
14 Lowest Frequency -2282.6
15 Nucleus 1H
16 Acquired Size 16384
17 Spectral Size 65536
18 Digital Resolution 0.12
13
f1 (ppm)

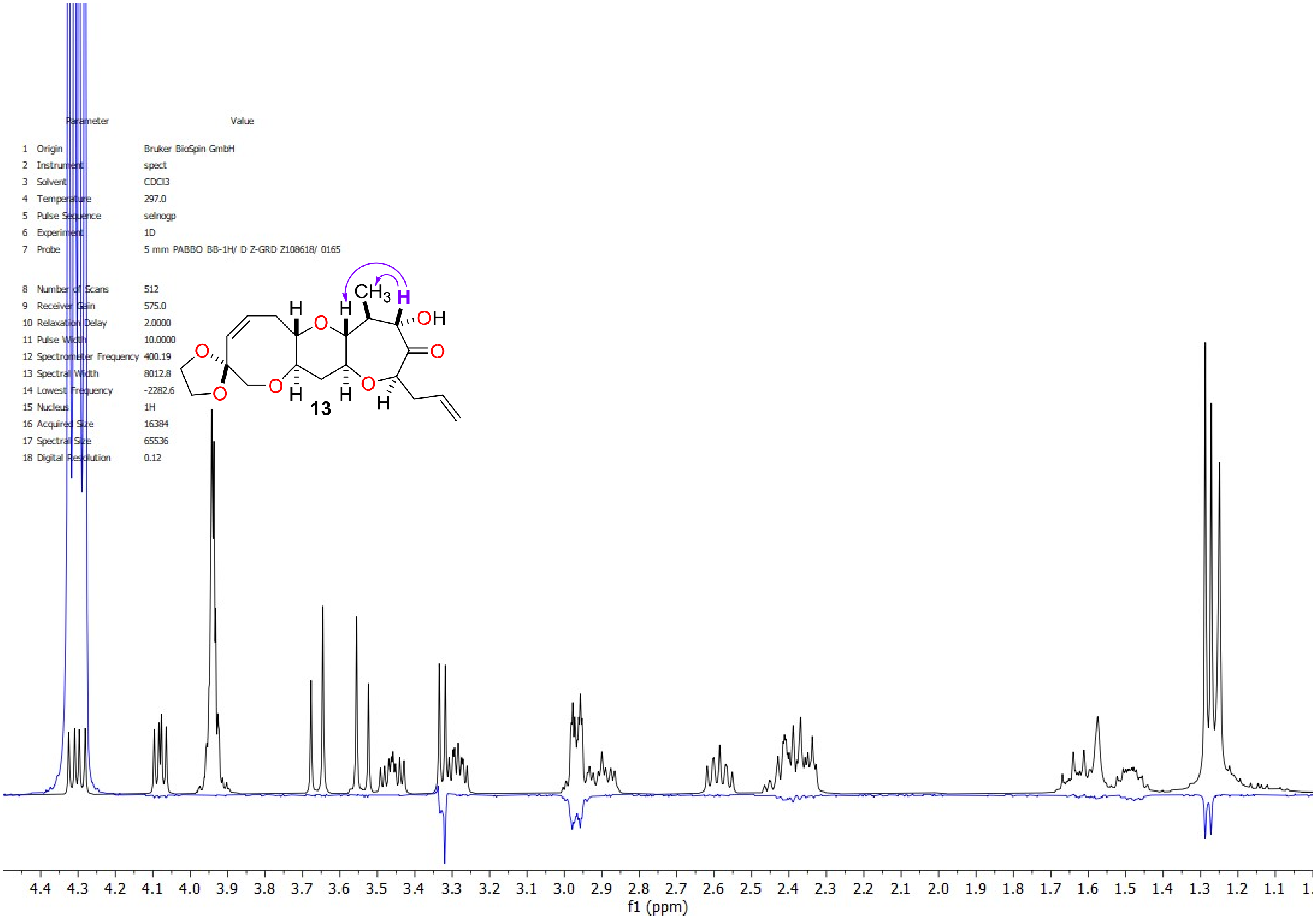
Parameter Value
1 Origin Bruker BioSpin GmbH
2 Instrument spect
3 Solvent CDCl3
4 Temperature 297.0
5 Pulse Sequence selnogp
6 Experiment 1D
7 Probe 5 mm PABBO BB-1H/ D Z-GRD Z108618/ 0165
8 Number of Scans 512
9 Receiver Gain 575.0
10 Relaxation Delay 2.0000
11 Pulse Width 10.0000
12 Spectrometer Frequency 400.19
13 Spectral Width 8012.8
14 Lowest Frequency -2282.6
15 Nucleus 1H
16 Acquired Size 16384
17 Spectral Size 65536
18 Digital Resolution 0.12
13
f1 (ppm)

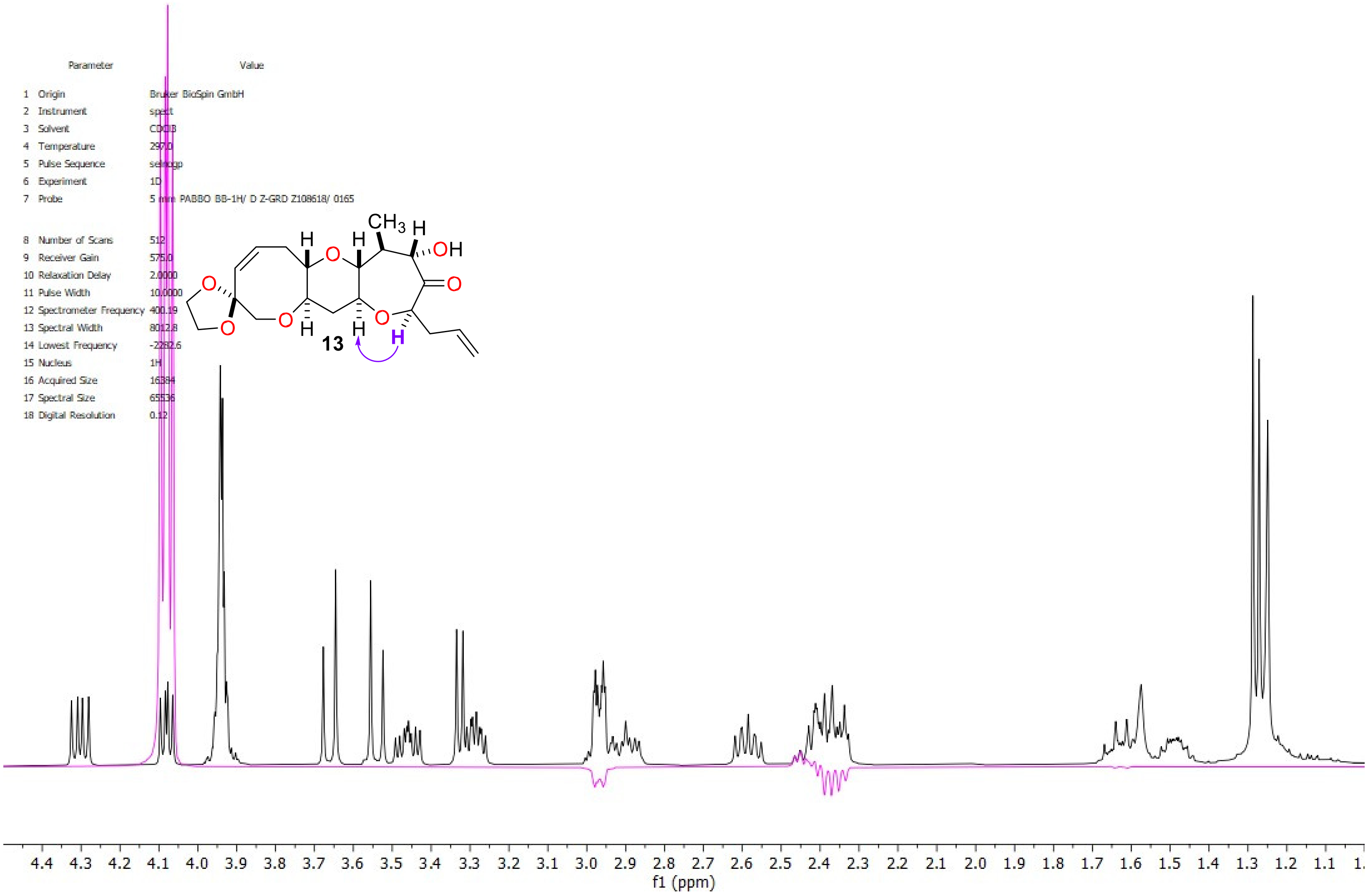
Parameter Value
1 Origin Bruker BioSpin GmbH
2 Instrument spect
3 Solvent CDCl3
4 Temperature 297.0
5 Pulse Sequence [illegible]
6 Experiment 1D
7 Probe 5 [illegible] PABBO BB-1H/ D Z-GRD Z108618/ 0165
8 Number of Scans 512
9 Receiver Gain 575.0
10 Relaxation Delay 2.0000
11 Pulse Width 10.0000
12 Spectrometer Frequency 400.19
13 Spectral Width 8012.8
14 Lowest Frequency -2282.6
15 Nucleus 1H
16 Acquired Size 16384
17 Spectral Size 65536
18 Digital Resolution 0.12
CH3
OH
13
4.4 4.3 4.2 4.1 4.0 3.9 3.8 3.7 3.6 3.5 3.4 3.3 3.2 3.1 3.0 2.9 2.8 2.7 2.6 2.5 2.4 2.3 2.2 2.1 2.0 1.9 1.8 1.7 1.6 1.5 1.4 1.3 1.2 1.1 1.
f1 (ppm)

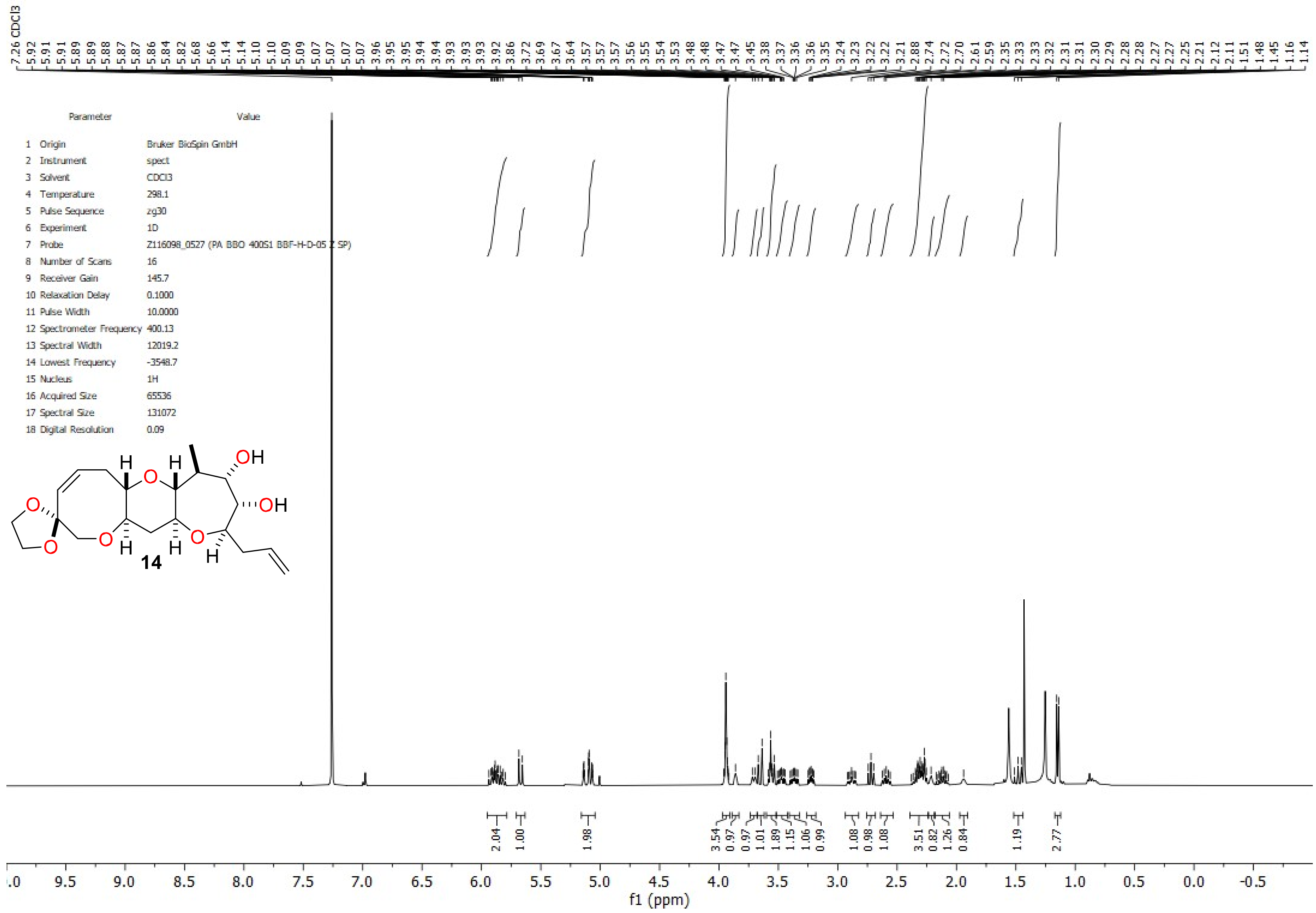
7.26 CDCl3
5.92 5.91 5.91 5.89 5.88 5.87 5.87 5.86 5.84 5.82 5.68 5.66 5.14 5.10 5.10 5.09 5.09 5.07 5.07 5.07 5.07 3.96 3.95 3.95 3.94 3.94 3.93 3.93 3.93 3.92 3.86 3.72 3.69 3.67 3.64 3.57 3.57 3.57 3.56 3.55 3.54 3.53 3.48 3.48 3.47 3.47 3.45 3.38 3.37 3.36 3.36 3.35 3.24 3.23 3.22 3.22 3.22 3.21 2.88 2.74 2.72 2.70 2.61 2.59 2.35 2.33 2.33 2.32 2.31 2.31 2.30 2.29 2.28 2.28 2.27 2.27 2.25 2.21 2.12 2.11 1.51 1.48 1.45 1.16 1.14
Parameter Value
1 Origin Bruker BioSpin GmbH
2 Instrument spect
3 Solvent CDCl3
4 Temperature 298.1
5 Pulse Sequence zg30
6 Experiment 1D
7 Probe Z116098_0527 (PA BBO 400S1 BBF-H-D-05 Z SP)
8 Number of Scans 16
9 Receiver Gain 145.7
10 Relaxation Delay 0.1000
11 Pulse Width 10.0000
12 Spectrometer Frequency 400.13
13 Spectral Width 12019.2
14 Lowest Frequency -3548.7
15 Nucleus 1H
16 Acquired Size 65536
17 Spectral Size 131072
18 Digital Resolution 0.09
14
2.04 1.00 1.98 3.54 0.97 0.97 1.01 1.89 1.15 1.06 0.99 1.08 0.98 1.08 3.51 0.82 1.26 0.84 1.19 2.77
.0 9.5 9.0 8.5 8.0 7.5 7.0 6.5 6.0 5.5 5.0 4.5 4.0 3.5 3.0 2.5 2.0 1.5 1.0 0.5 0.0 -0.5
f1 (ppm)

	Parameter	Value
1	Origin	Bruker BioSpin GmbH
2	Instrument	spect
3	Solvent	CDCl3
4	Temperature	298.1
5	Pulse Sequence	zgpg30
6	Experiment	1D
7	Probe	Z116098_0527 (PA BBO 400S1 BBF-H-D-05 Z SP)
8	Number of Scans	8192
9	Receiver Gain	212.9
10	Relaxation Delay	1.0000
11	Pulse Width	10.0000
12	Spectrometer Frequency	100.62
13	Spectral Width	25000.0
14	Lowest Frequency	-1419.3
15	Nucleus	13C
16	Acquired Size	59522
17	Spectral Size	131072
18	Digital Resolution	0.19

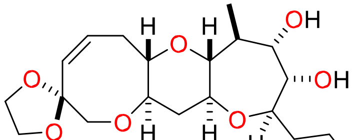

14

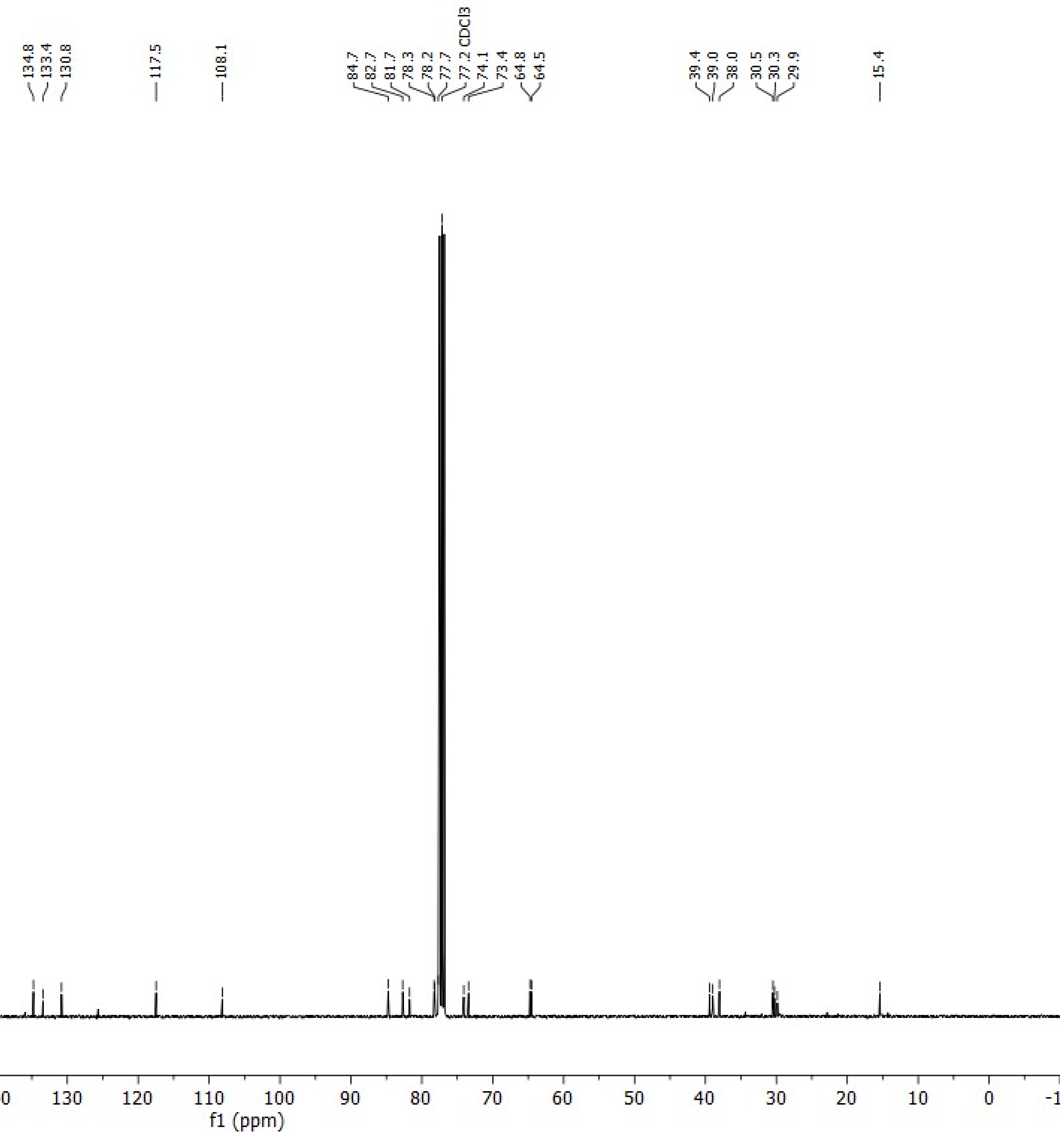

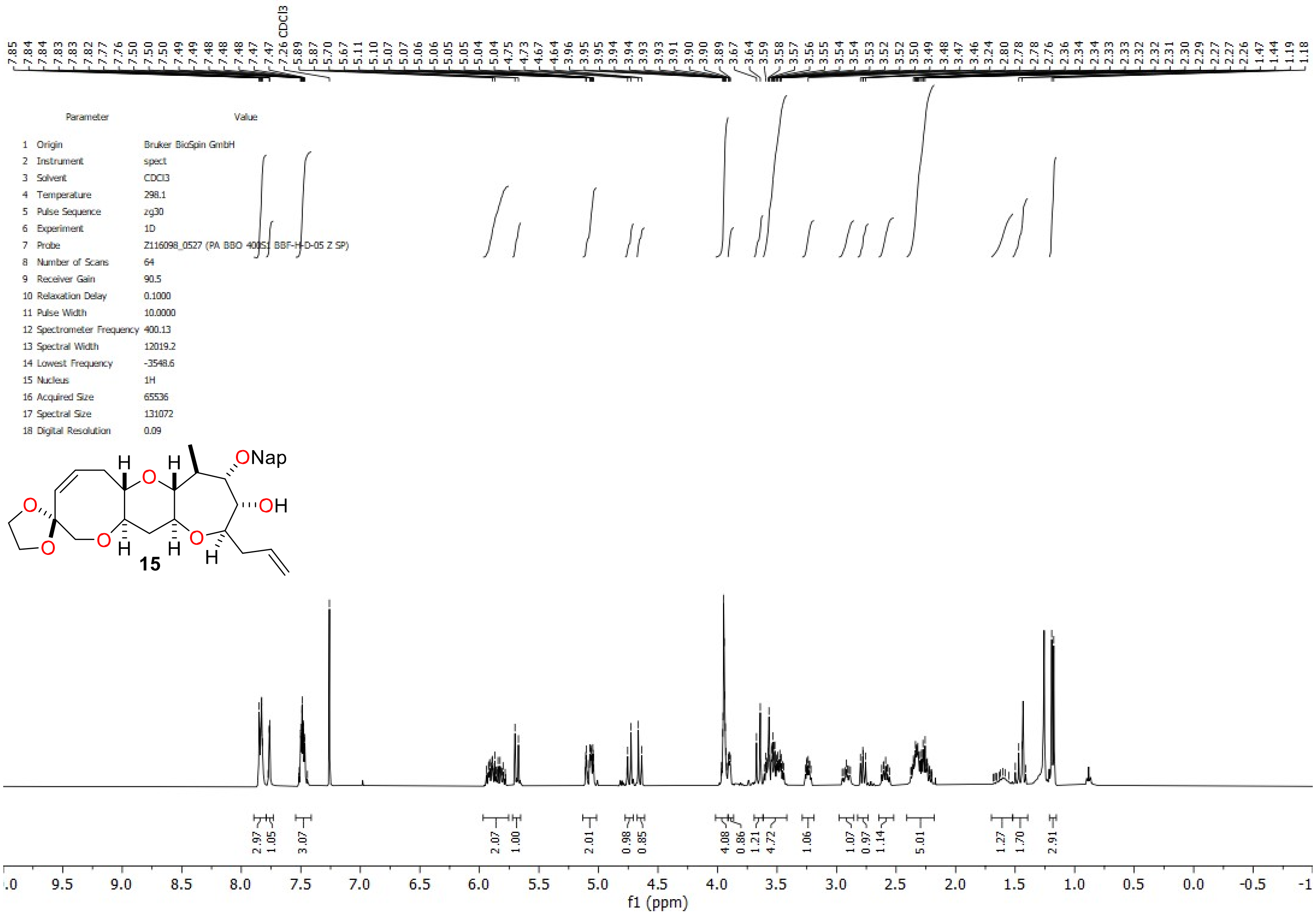
CDCl3
Parameter Value
1 Origin Bruker BioSpin GmbH
2 Instrument spect
3 Solvent CDCl3
4 Temperature 298.1
5 Pulse Sequence zg30
6 Experiment 1D
7 Probe Z116098_0527 (PA BBO 400S1 BBF-H-D-05 Z SP)
8 Number of Scans 64
9 Receiver Gain 90.5
10 Relaxation Delay 0.1000
11 Pulse Width 10.0000
12 Spectrometer Frequency 400.13
13 Spectral Width 12019.2
14 Lowest Frequency -3548.6
15 Nucleus 1H
16 Acquired Size 65536
17 Spectral Size 131072
18 Digital Resolution 0.09
ONap
OH
15
f1 (ppm)

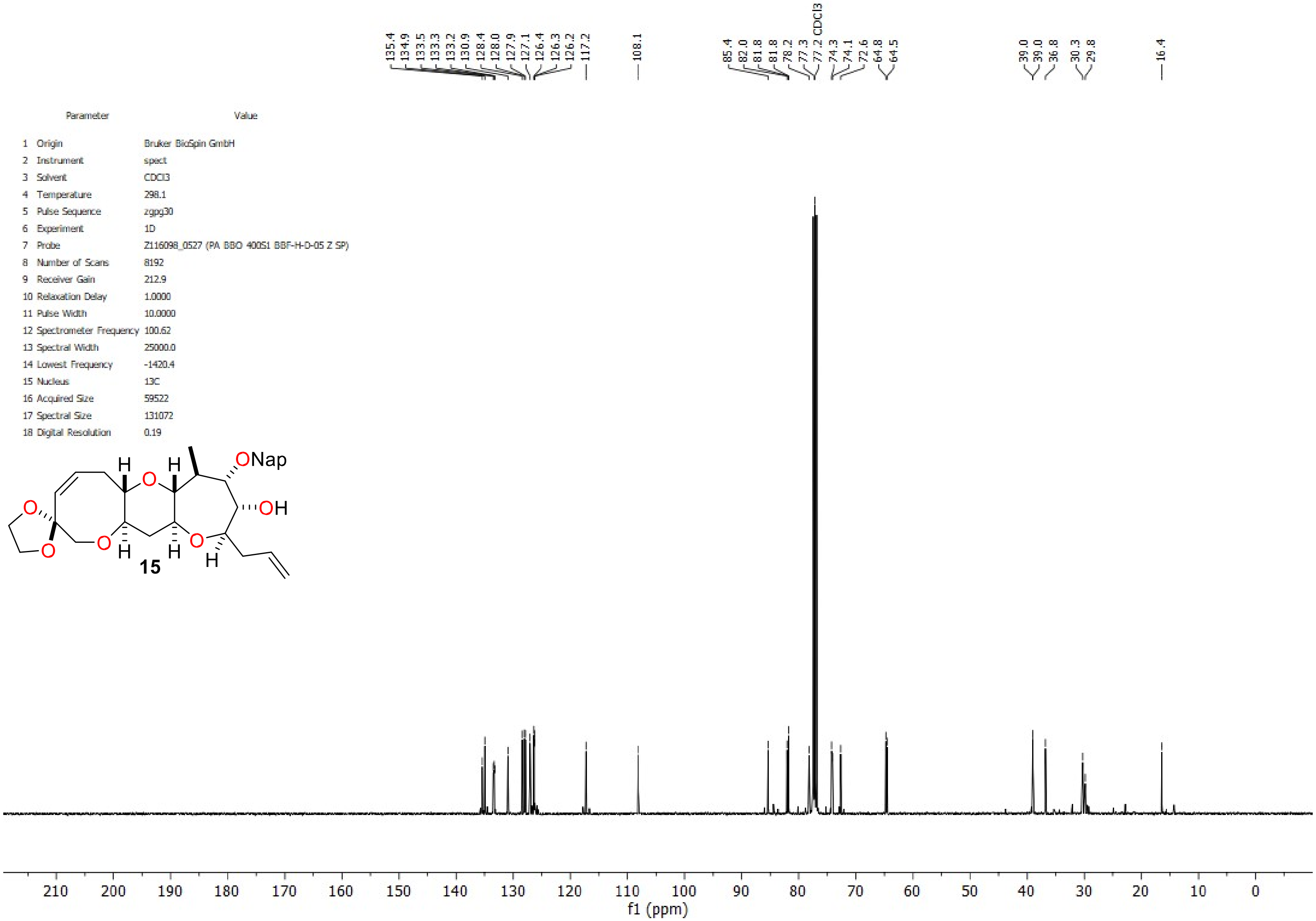
135.4
134.9
133.5
133.3
133.2
130.9
128.4
128.0
127.9
127.1
126.4
126.3
126.2
117.2
108.1
85.4
82.0
81.8
81.8
78.2
77.3
77.2 CDCl3
74.3
74.1
72.6
64.8
64.5
39.0
39.0
36.8
30.3
29.8
16.4
Parameter Value
1 Origin Bruker BioSpin GmbH
2 Instrument spect
3 Solvent CDCl3
4 Temperature 298.1
5 Pulse Sequence zgpg30
6 Experiment 1D
7 Probe Z116098_0527 (PA BBO 400S1 BBF-H-D-05 Z SP)
8 Number of Scans 8192
9 Receiver Gain 212.9
10 Relaxation Delay 1.0000
11 Pulse Width 10.0000
12 Spectrometer Frequency 100.62
13 Spectral Width 25000.0
14 Lowest Frequency -1420.4
15 Nucleus 13C
16 Acquired Size 59522
17 Spectral Size 131072
18 Digital Resolution 0.19
ONap
OH
15
210 200 190 180 170 160 150 140 130 120 110 100 90 80 70 60 50 40 30 20 10 0
f1 (ppm)

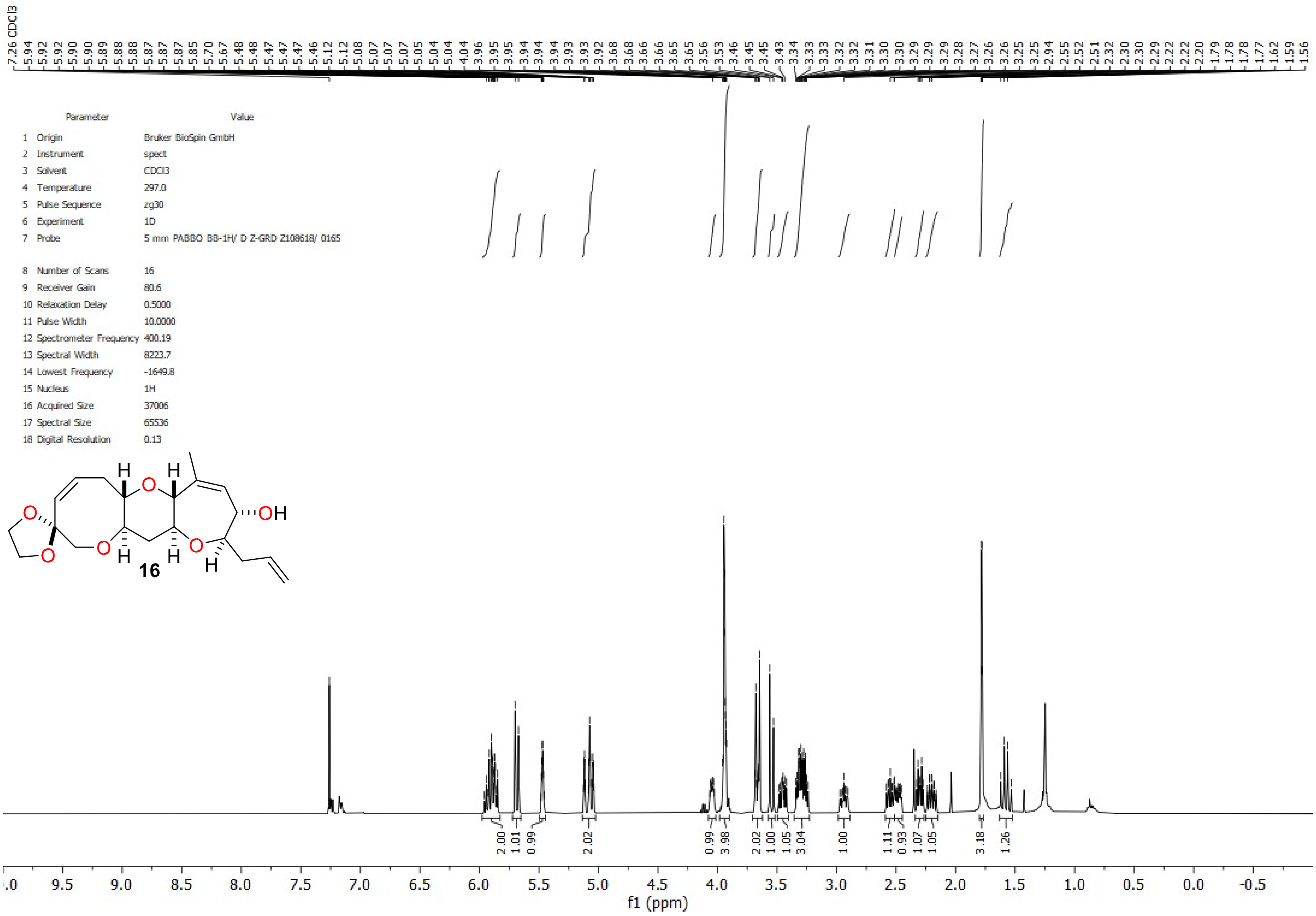

Parameter Value
1 Origin Bruker BioSpin GmbH
2 Instrument spect
3 Solvent CDCl3
4 Temperature 297.0
5 Pulse Sequence zg30
6 Experiment 1D
7 Probe 5 mm PABBO BB-1H/ D Z-GRD Z108618/ 0165
8 Number of Scans 16
9 Receiver Gain 80.6
10 Relaxation Delay 0.5000
11 Pulse Width 10.0000
12 Spectrometer Frequency 400.19
13 Spectral Width 8223.7
14 Lowest Frequency -1649.8
15 Nucleus 1H
16 Acquired Size 37006
17 Spectral Size 65536
18 Digital Resolution 0.13
16
f1 (ppm)

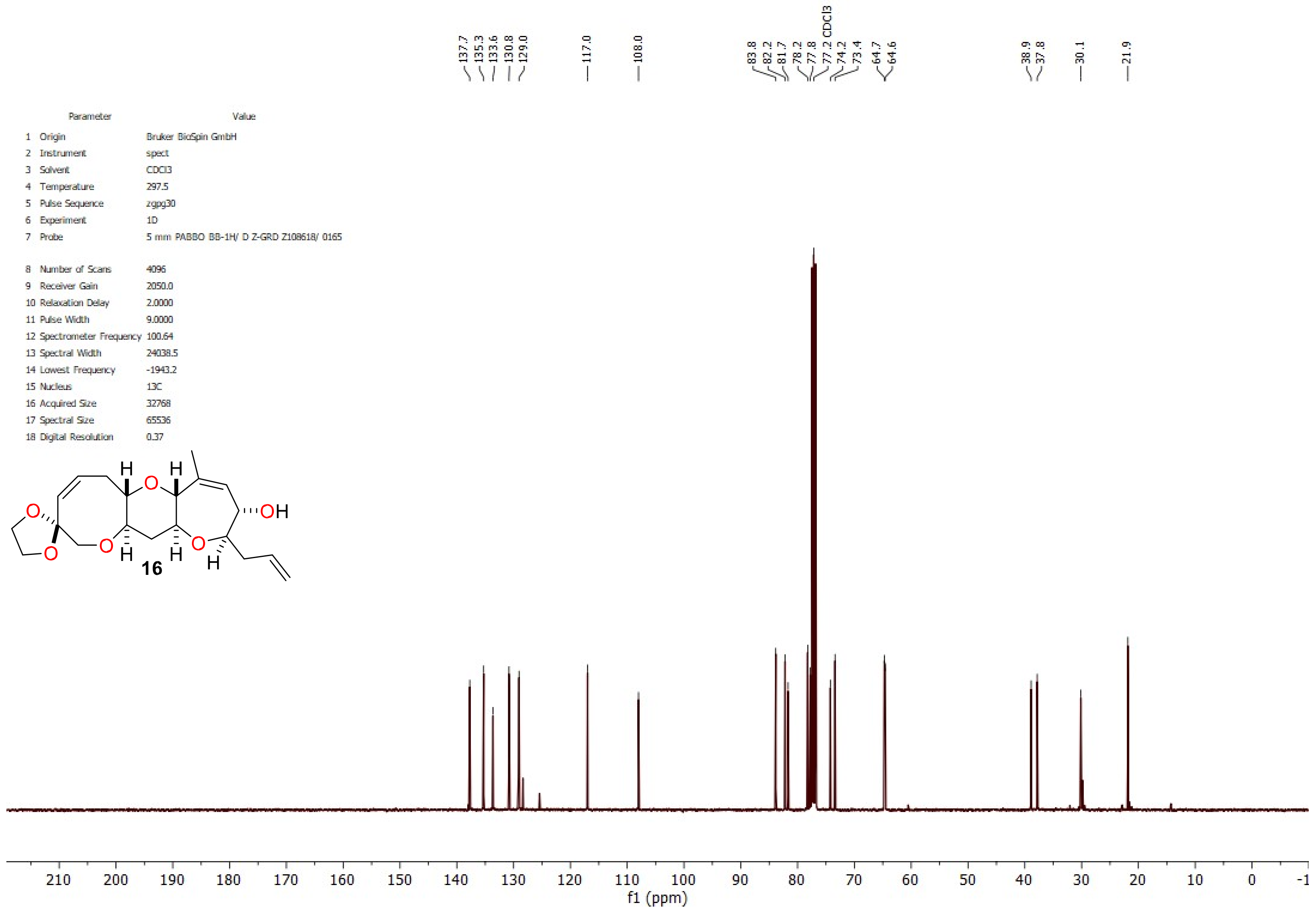
137.7
135.3
133.6
130.8
129.0
117.0
108.0
83.8
82.2
81.7
78.2
77.8
77.2 CDCl3
74.2
73.4
64.7
64.6
38.9
37.8
30.1
21.9
Parameter Value
1 Origin Bruker BioSpin GmbH
2 Instrument spect
3 Solvent CDCl3
4 Temperature 297.5
5 Pulse Sequence zgpg30
6 Experiment 1D
7 Probe 5 mm PABBO BB-1H/ D Z-GRD Z108618/ 0165
8 Number of Scans 4096
9 Receiver Gain 2050.0
10 Relaxation Delay 2.0000
11 Pulse Width 9.0000
12 Spectrometer Frequency 100.64
13 Spectral Width 24038.5
14 Lowest Frequency -1943.2
15 Nucleus 13C
16 Acquired Size 32768
17 Spectral Size 65536
18 Digital Resolution 0.37
16
210 200 190 180 170 160 150 140 130 120 110 100 90 80 70 60 50 40 30 20 10 0 -1
f1 (ppm)

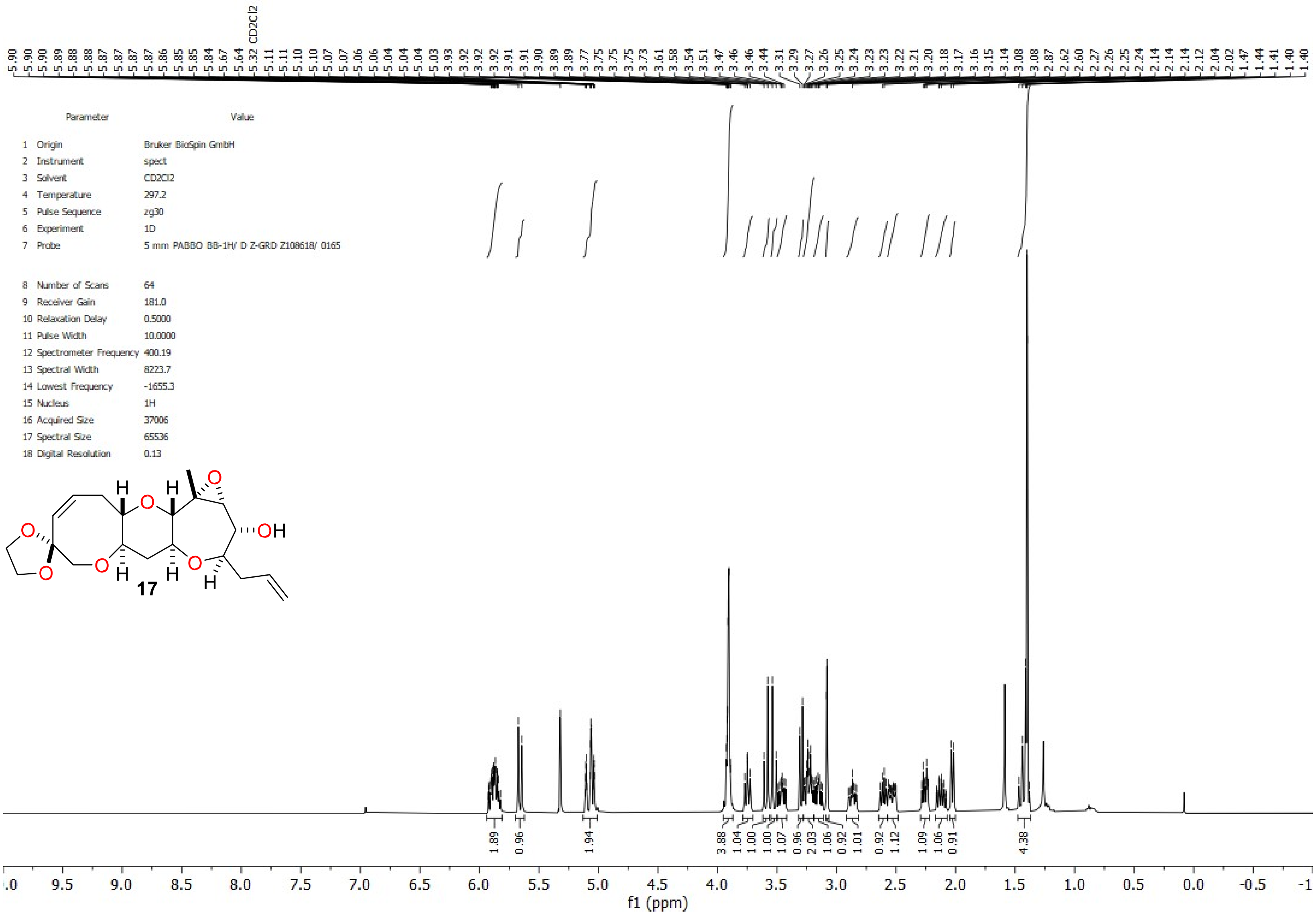

Parameter		Value
1	Origin	Bruker BioSpin GmbH
2	Instrument	spect
3	Solvent	CD2Cl2
4	Temperature	297.2
5	Pulse Sequence	zg30
6	Experiment	1D
7	Probe	5 mm PABBO BB-1H/ D Z-GRD Z108618/ 0165
8	Number of Scans	64
9	Receiver Gain	181.0
10	Relaxation Delay	0.5000
11	Pulse Width	10.0000
12	Spectrometer Frequency	400.19
13	Spectral Width	8223.7
14	Lowest Frequency	-1655.3
15	Nucleus	1H
16	Acquired Size	37006
17	Spectral Size	65536
18	Digital Resolution	0.13

Parameter	Value
1 Origin	Bruker BioSpin GmbH
2 Instrument	spect
3 Solvent	CD2Cl2
4 Temperature	297.2
5 Pulse Sequence	zgpg30
6 Experiment	1D
7 Probe	5 mm PABBO BB-1H/ D Z-GRD Z108618/ 0165
8 Number of Scans	5120
9 Receiver Gain	2050.0
10 Relaxation Delay	2.0000
11 Pulse Width	9.0000
12 Spectrometer Frequency	100.64
13 Spectral Width	24038.5
14 Lowest Frequency	-1913.5
15 Nucleus	13C
16 Acquired Size	32768
17 Spectral Size	65536
18 Digital Resolution	0.37

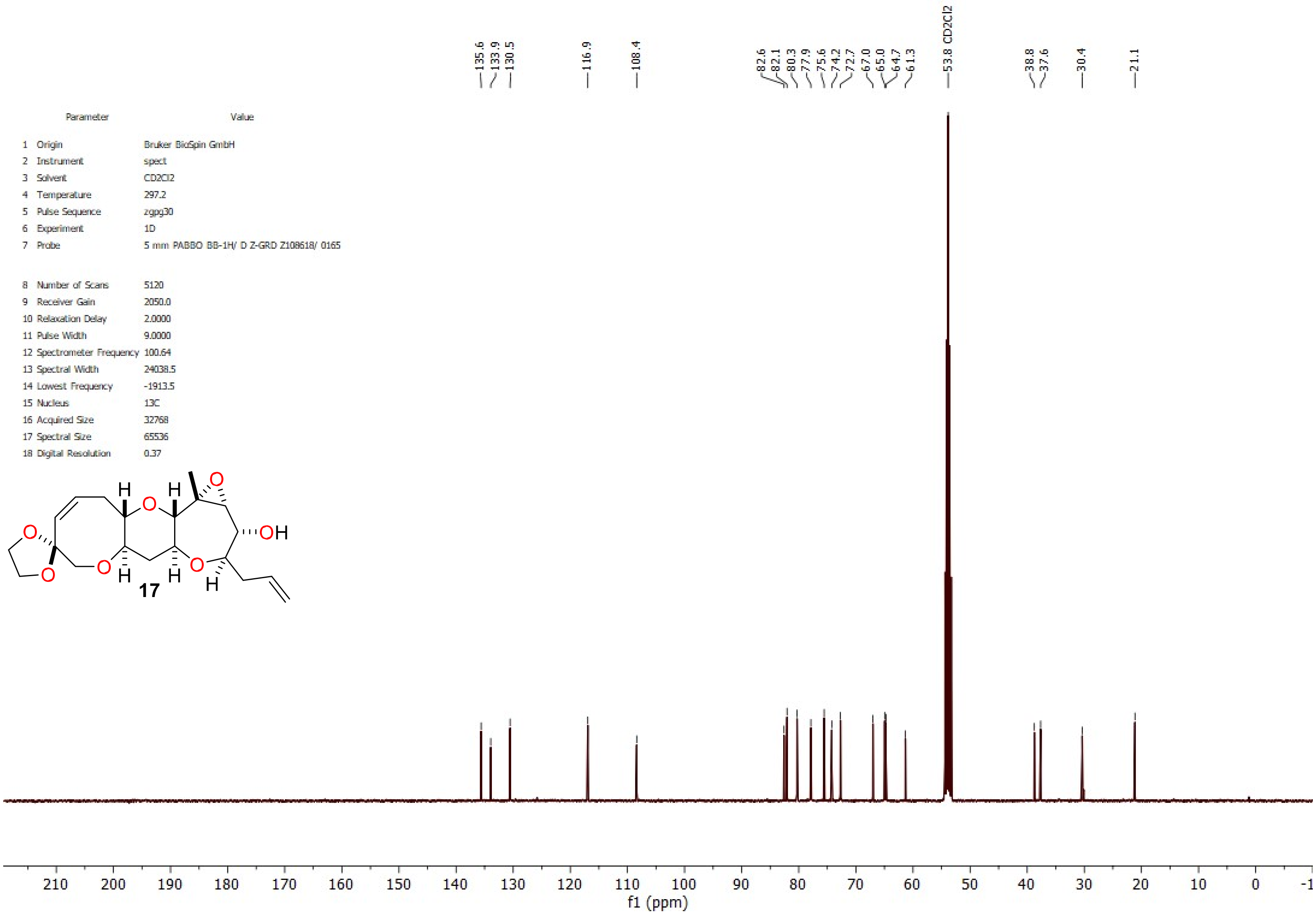

17

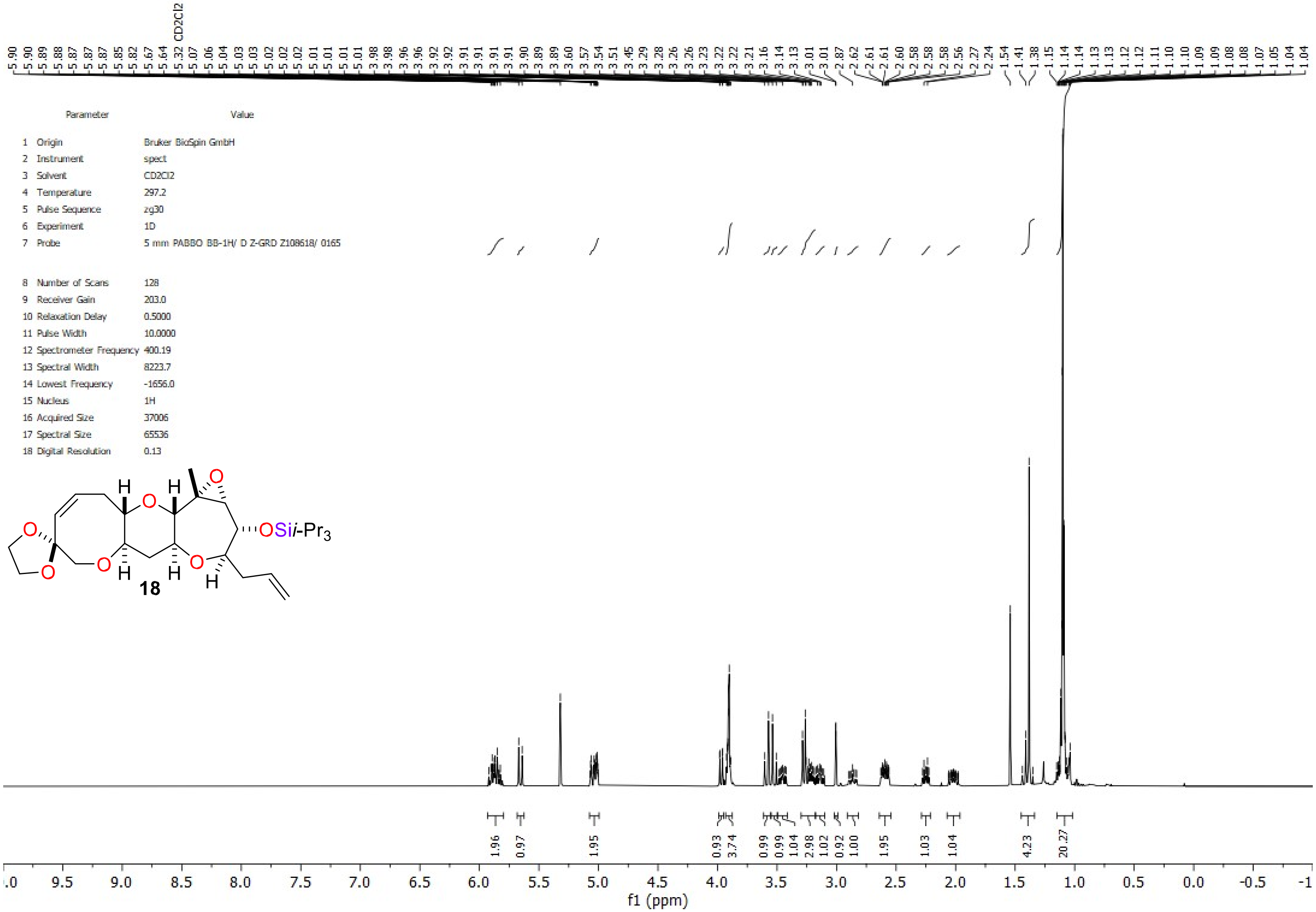

CD2Cl2
Parameter Value
1 Origin Bruker BioSpin GmbH
2 Instrument spect
3 Solvent CD2Cl2
4 Temperature 297.2
5 Pulse Sequence zg30
6 Experiment 1D
7 Probe 5 mm PABBO BB-1H/ D Z-GRD Z108618/ 0165
8 Number of Scans 128
9 Receiver Gain 203.0
10 Relaxation Delay 0.5000
11 Pulse Width 10.0000
12 Spectrometer Frequency 400.19
13 Spectral Width 8223.7
14 Lowest Frequency -1656.0
15 Nucleus 1H
16 Acquired Size 37006
17 Spectral Size 65536
18 Digital Resolution 0.13
OSi*i*-Pr3
18
f1 (ppm)

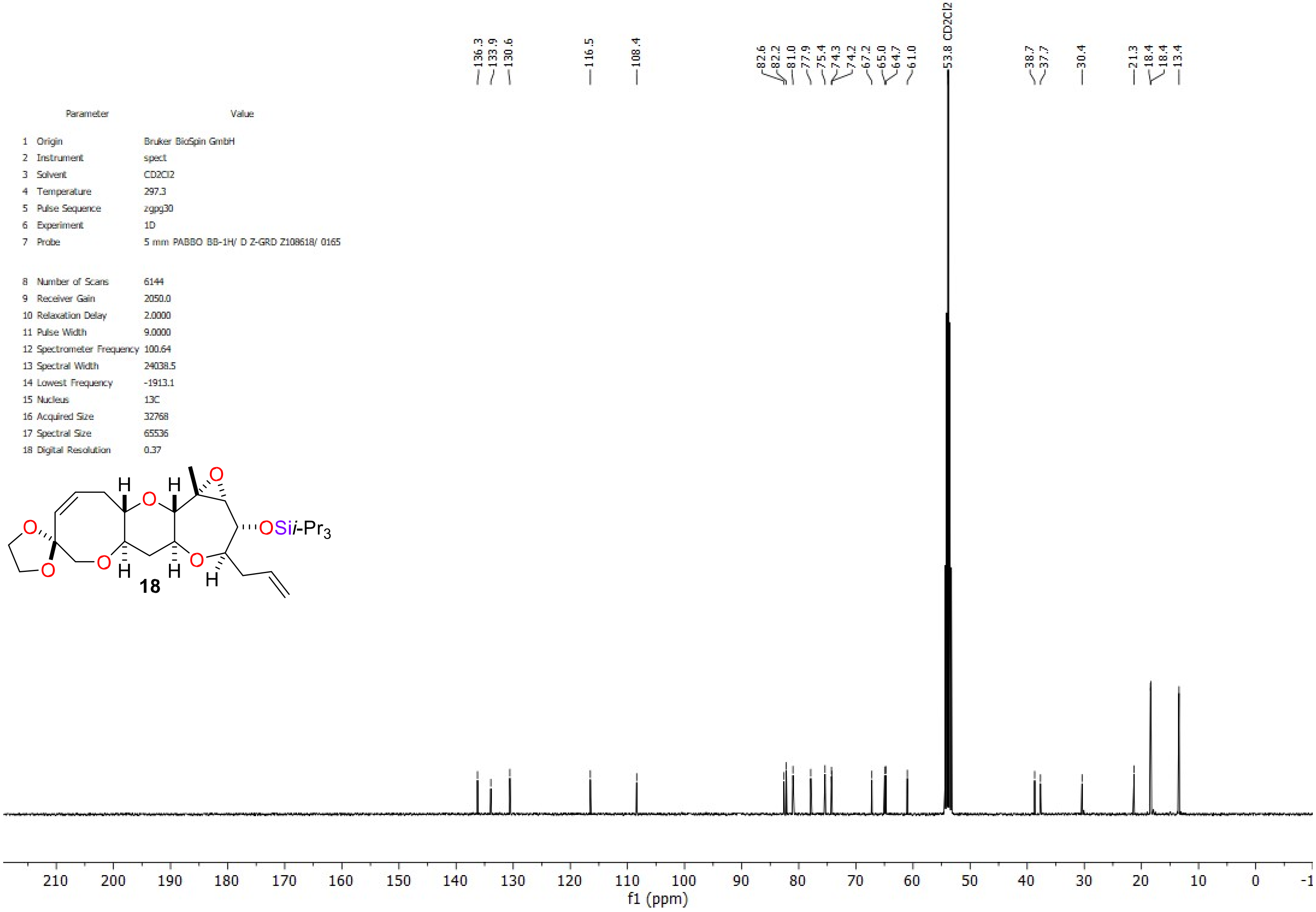

	Parameter	Value
1	Origin	Bruker BioSpin GmbH
2	Instrument	spect
3	Solvent	CD2Cl2
4	Temperature	297.3
5	Pulse Sequence	zgpg30
6	Experiment	1D
7	Probe	5 mm PABBO BB-1H/ D Z-GRD Z108618/ 0165
8	Number of Scans	6144
9	Receiver Gain	2050.0
10	Relaxation Delay	2.0000
11	Pulse Width	9.0000
12	Spectrometer Frequency	100.64
13	Spectral Width	24038.5
14	Lowest Frequency	-1913.1
15	Nucleus	13C
16	Acquired Size	32768
17	Spectral Size	65536
18	Digital Resolution	0.37

18

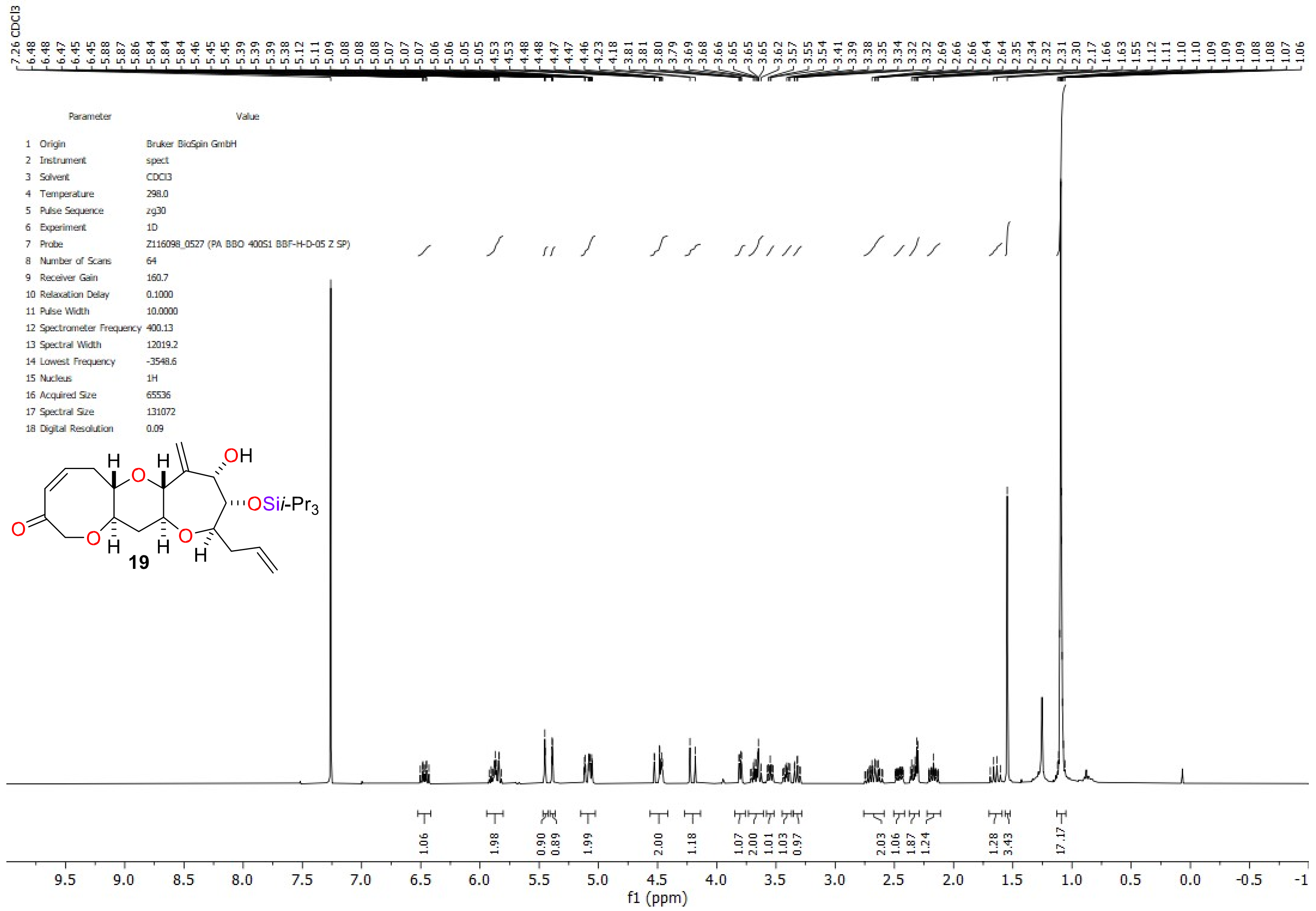
Parameter
Value
1 Origin Bruker BioSpin GmbH
2 Instrument spect
3 Solvent CDCl3
4 Temperature 298.0
5 Pulse Sequence zg30
6 Experiment 1D
7 Probe Z116098_0527 (PA BBO 400S1 BBF-H-D-05 Z SP)
8 Number of Scans 64
9 Receiver Gain 160.7
10 Relaxation Delay 0.1000
11 Pulse Width 10.0000
12 Spectrometer Frequency 400.13
13 Spectral Width 12019.2
14 Lowest Frequency -3548.6
15 Nucleus 1H
16 Acquired Size 65536
17 Spectral Size 131072
18 Digital Resolution 0.09
OH
OSi*i*-Pr3
19
f1 (ppm)

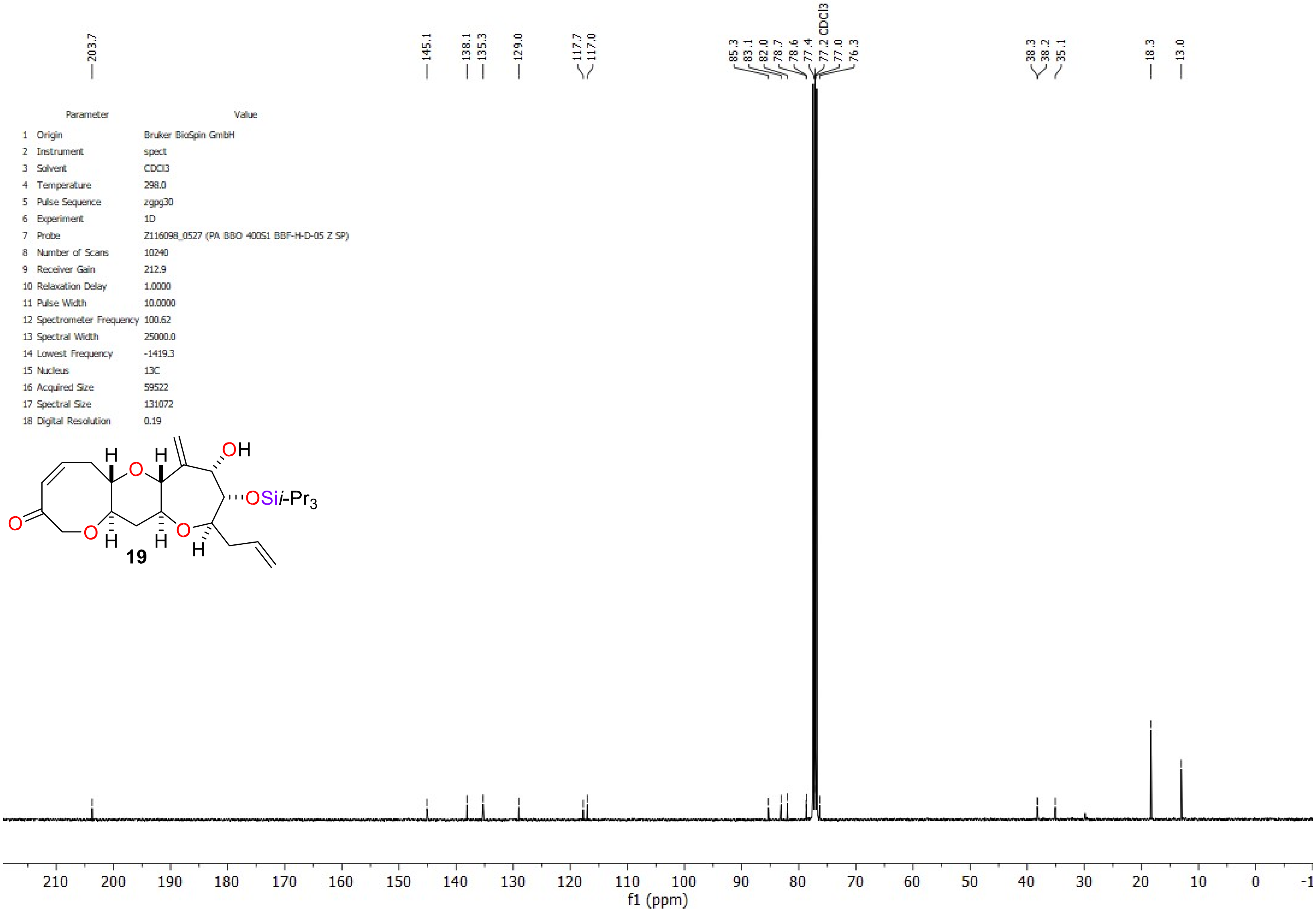
203.7
145.1
138.1
135.3
129.0
117.7
117.0
85.3
83.1
82.0
78.7
78.6
77.4
77.2 CDCl3
77.0
76.3
38.3
38.2
35.1
18.3
13.0
Parameter Value
1 Origin Bruker BioSpin GmbH
2 Instrument spect
3 Solvent CDCl3
4 Temperature 298.0
5 Pulse Sequence zgpg30
6 Experiment 1D
7 Probe Z116098_0527 (PA BBO 400S1 BBF-H-D-05 Z SP)
8 Number of Scans 10240
9 Receiver Gain 212.9
10 Relaxation Delay 1.0000
11 Pulse Width 10.0000
12 Spectrometer Frequency 100.62
13 Spectral Width 25000.0
14 Lowest Frequency -1419.3
15 Nucleus 13C
16 Acquired Size 59522
17 Spectral Size 131072
18 Digital Resolution 0.19
H
O
H
OH
OSii-Pr3
O
O
H
H
O
H
19
210 200 190 180 170 160 150 140 130 120 110 100 90 80 70 60 50 40 30 20 10 0 -1
f1 (ppm)

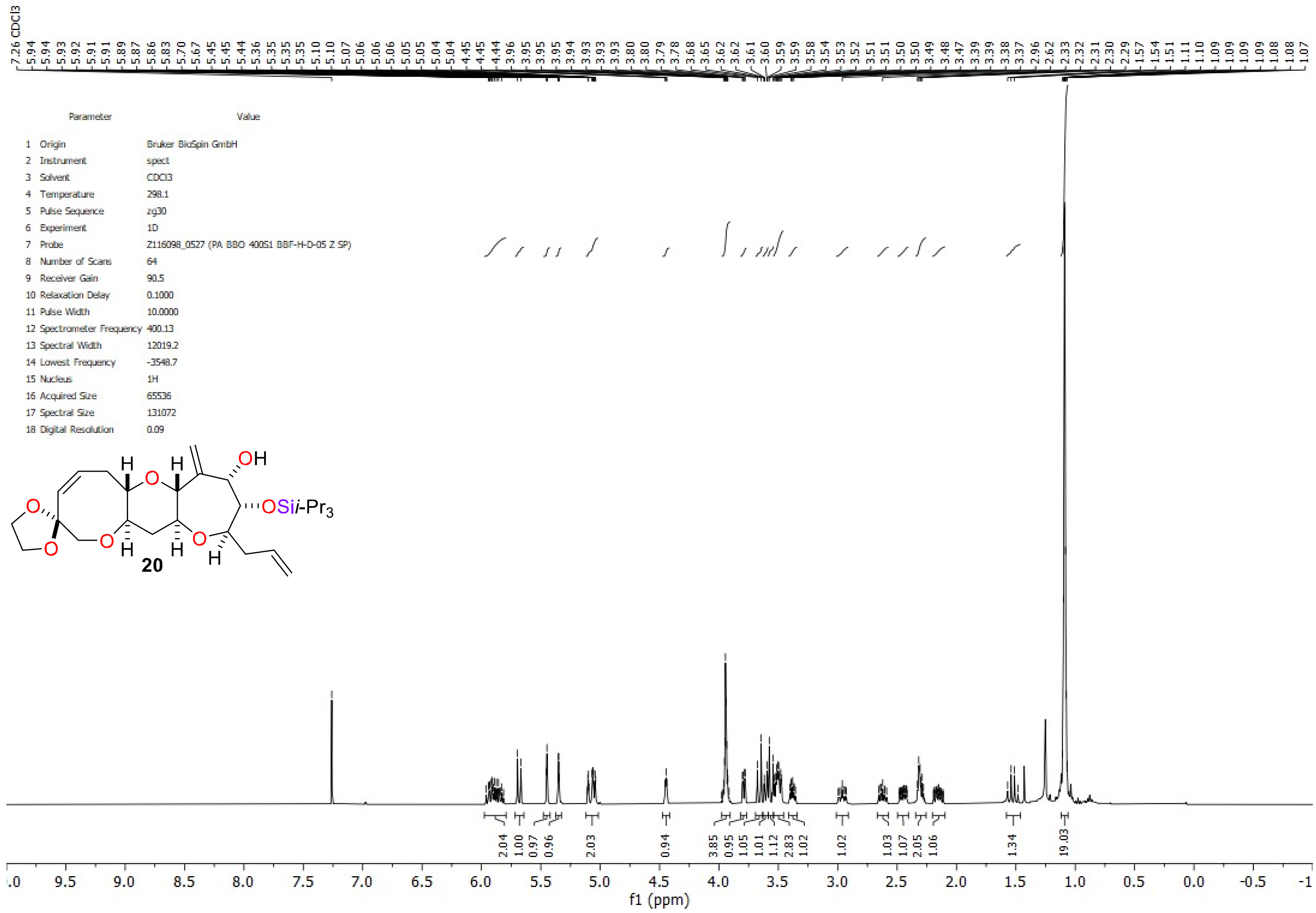

Parameter Value
1 Origin Bruker BioSpin GmbH
2 Instrument spect
3 Solvent CDCl3
4 Temperature 298.1
5 Pulse Sequence zg30
6 Experiment 1D
7 Probe Z116098_0527 (PA BBO 400S1 BBF-H-D-05 Z SP)
8 Number of Scans 64
9 Receiver Gain 90.5
10 Relaxation Delay 0.1000
11 Pulse Width 10.0000
12 Spectrometer Frequency 400.13
13 Spectral Width 12019.2
14 Lowest Frequency -3548.7
15 Nucleus 1H
16 Acquired Size 65536
17 Spectral Size 131072
18 Digital Resolution 0.09
OH
OSi*i*-Pr3
20
f1 (ppm)

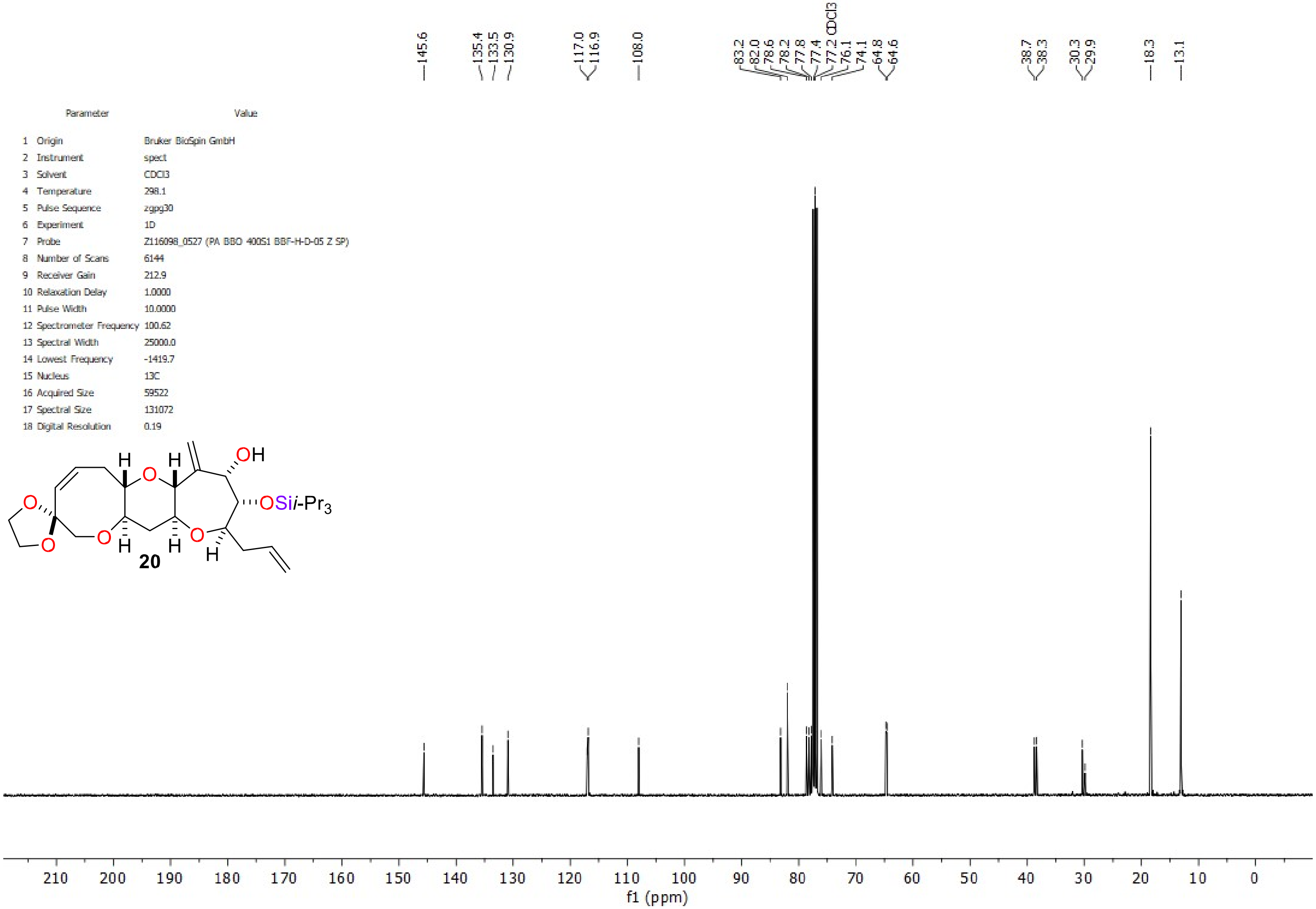

	Parameter	Value
1	Origin	Bruker BioSpin GmbH
2	Instrument	spect
3	Solvent	CDCl3
4	Temperature	298.1
5	Pulse Sequence	zgpg30
6	Experiment	1D
7	Probe	Z116098_0527 (PA BBO 400S1 BBF-H-D-05 Z SP)
8	Number of Scans	6144
9	Receiver Gain	212.9
10	Relaxation Delay	1.0000
11	Pulse Width	10.0000
12	Spectrometer Frequency	100.62
13	Spectral Width	25000.0
14	Lowest Frequency	-1419.7
15	Nucleus	13C
16	Acquired Size	59522
17	Spectral Size	131072
18	Digital Resolution	0.19

20

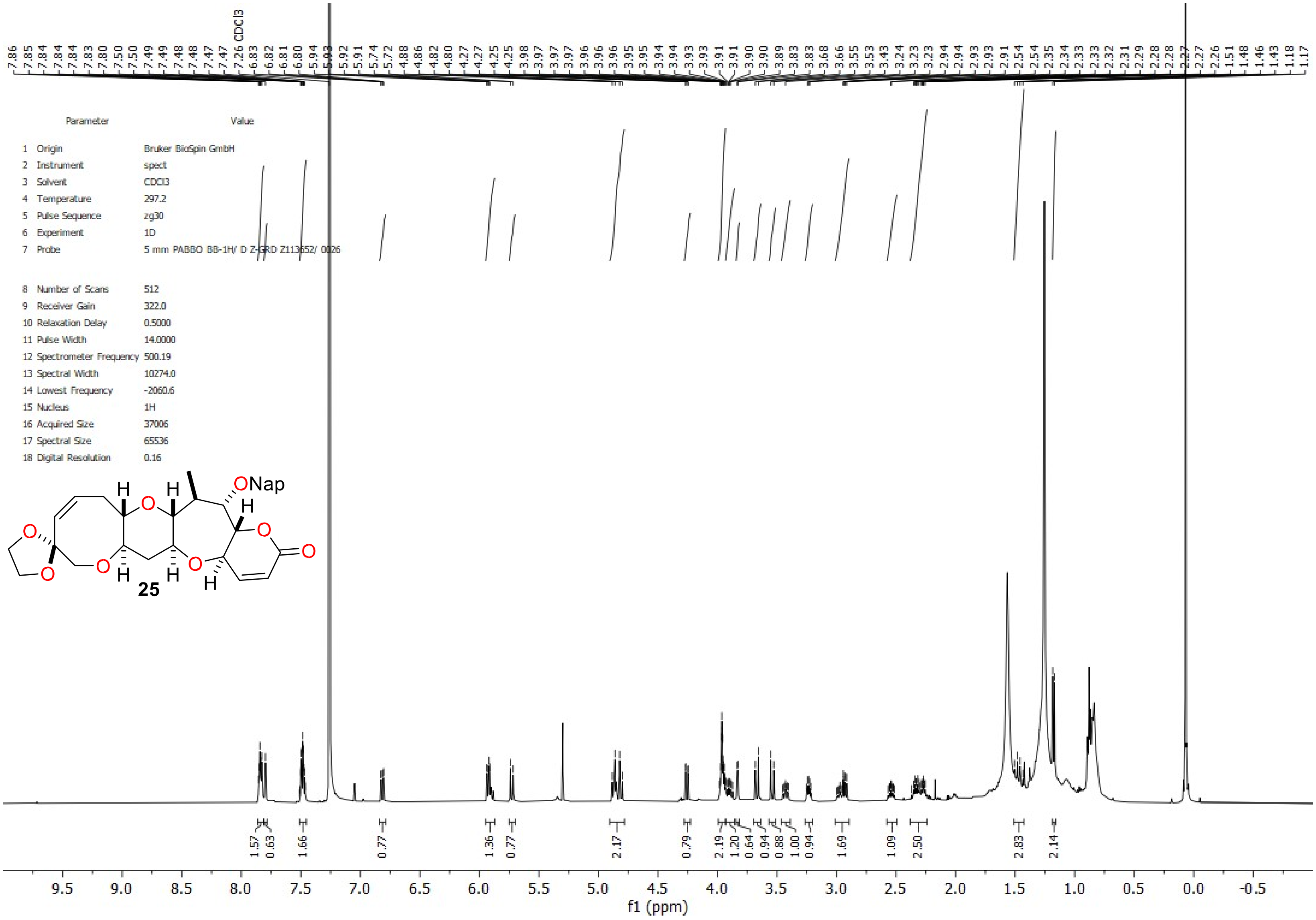
Parameter Value
1 Origin Bruker BioSpin GmbH
2 Instrument spect
3 Solvent CDCl3
4 Temperature 297.2
5 Pulse Sequence zg30
6 Experiment 1D
7 Probe 5 mm PABBO BB-1H/ D Z-GRD Z113652/ 0026
8 Number of Scans 512
9 Receiver Gain 322.0
10 Relaxation Delay 0.5000
11 Pulse Width 14.0000
12 Spectrometer Frequency 500.19
13 Spectral Width 10274.0
14 Lowest Frequency -2060.6
15 Nucleus 1H
16 Acquired Size 37006
17 Spectral Size 65536
18 Digital Resolution 0.16
ONap
25
CDCl3
f1 (ppm)

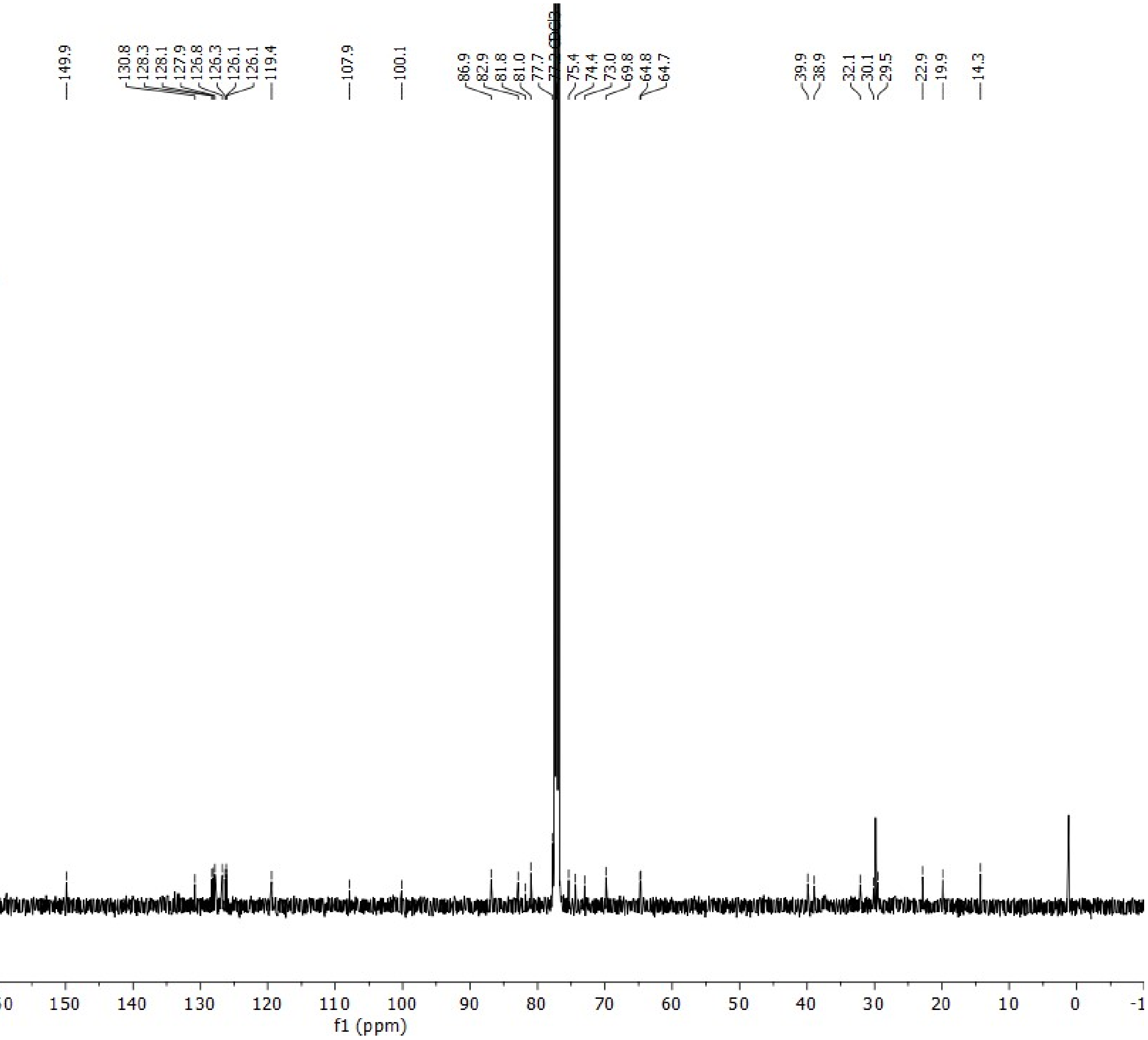

	Parameter	Value
1	Origin	Bruker BioSpin GmbH
2	Instrument	spect
3	Solvent	CDCl3
4	Temperature	297.2
5	Pulse Sequence	zgpg30
6	Experiment	1D
7	Probe	5 mm PABBO BB-1H/ D Z-GRD Z113652/ 0026
8	Number of Scans	25000
9	Receiver Gain	2050.0
10	Relaxation Delay	2.0000
11	Pulse Width	8.7000
12	Spectrometer Frequency	125.79
13	Spectral Width	30000.0
14	Lowest Frequency	-2404.2
15	Nucleus	13C
16	Acquired Size	32768
17	Spectral Size	65536
18	Digital Resolution	0.46

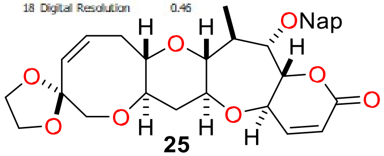

25
